# Finding Correspondence between Metabolomic Features in Untargeted Liquid Chromatography–Mass Spectrometry Metabolomics Datasets

**DOI:** 10.1021/acs.analchem.1c03592

**Published:** 2022-03-31

**Authors:** Rui Climaco Pinto, Ibrahim Karaman, Matthew R. Lewis, Jenny Hällqvist, Manuja Kaluarachchi, Gonçalo Graça, Elena Chekmeneva, Brenan Durainayagam, Mohsen Ghanbari, M. Arfan Ikram, Henrik Zetterberg, Julian Griffin, Paul Elliott, Ioanna Tzoulaki, Abbas Dehghan, David Herrington, Timothy Ebbels

**Affiliations:** † Department of Epidemiology and Biostatistics, MRC-PHE Centre for Environment and Health, School of Public Health, Imperial College London, London W12 0BZ, U.K.; ‡ UK Dementia Research Institute, Imperial College London, London W12 0BZ, U.K.; § MRC-NIHR National Phenome Centre, Department of Metabolism, Digestion and Reproduction, Imperial College London, London SW7 2AZ, U.K.; ∥ Section of Bioanalytical Chemistry, Department of Metabolism, Digestion and Reproduction, Imperial College London, London SW7 2AZ, U.K.; ⊥ Centre for Translational Omics, Great Ormond Street Hospital, University College London, London WC1N 1EH, U.K.; # Department of Clinical and Movement Neurosciences, Queen Square Institute of Neurology, University College London, London WC1N 3BG, U.K.; ∇ Section of Bioinformatics, Division of Systems Medicine, Department of Metabolism, Digestion and Reproduction, Imperial College London, London SW7 2AZ, U.K.; ○ Department of Epidemiology, Erasmus University Medical Center, 3015 GD Rotterdam, The Netherlands; ◆ Department of Psychiatry and Neurochemistry, Institute of Neuroscience and Physiology, The Sahlgrenska Academy at University of Gothenburg, 431 41 Mölndal, Sweden; ¶ Clinical Neurochemistry Laboratory, Sahlgrenska University Hospital, 413 45 Mölndal, Sweden; ⋈ Department of Neurodegenerative Disease, University College London, Queen Square, London WC1N 3BG, U.K.; ⧓ UK Dementia Research Institute, University College London, London WC1N 3BG, U.K.; ⧖ Department of Hygiene and Epidemiology, University of Ioannina School of Medicine, 451 10 Ioannina, Greece; ● Department of Internal Medicine, Wake Forest School of Medicine, Winston-Salem, North Carolina 27101, United States

## Abstract

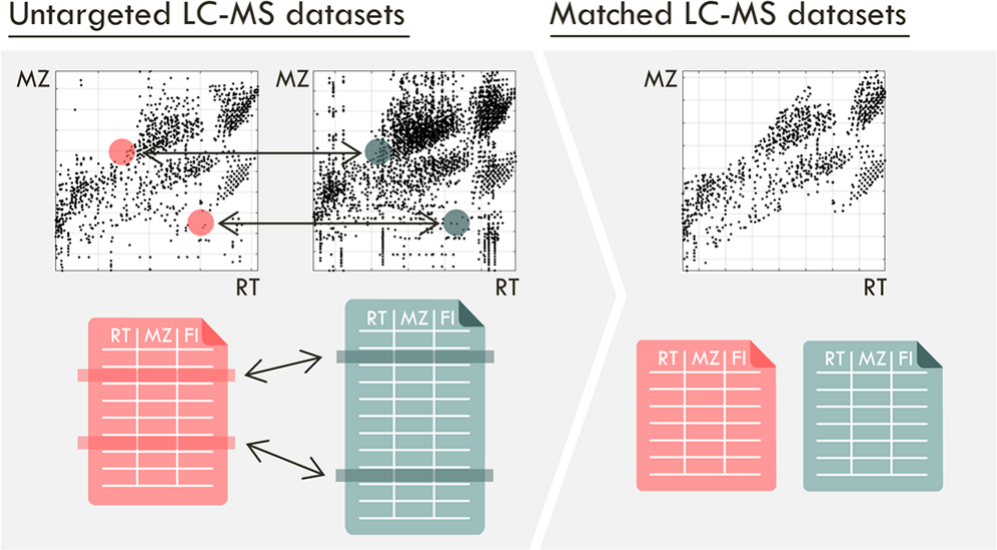

Integration of multiple datasets can greatly enhance bioanalytical studies, for example, by increasing power to discover and validate biomarkers. In liquid chromatography–mass spectrometry (LC–MS) metabolomics, it is especially hard to combine untargeted datasets since the majority of metabolomic features are not annotated and thus cannot be matched by chemical identity. Typically, the information available for each feature is retention time (RT), mass-to-charge ratio (*m*/*z*), and feature intensity (FI). Pairs of features from the same metabolite in separate datasets can exhibit small but significant differences, making matching very challenging. Current methods to address this issue are too simple or rely on assumptions that cannot be met in all cases. We present a method to find feature correspondence between two similar LC–MS metabolomics experiments or batches using only the features’ RT, *m*/*z*, and FI. We demonstrate the method on both real and synthetic datasets, using six orthogonal validation strategies to gauge the matching quality. In our main example, 4953 features were uniquely matched, of which 585 (96.8%) of 604 manually annotated features were correct. In a second example, 2324 features could be uniquely matched, with 79 (90.8%) out of 87 annotated features correctly matched. Most of the missed annotated matches are between features that behave very differently from modeled inter-dataset shifts of RT, MZ, and FI. In a third example with simulated data with 4755 features per dataset, 99.6% of the matches were correct. Finally, the results of matching three other dataset pairs using our method are compared with a published alternative method, metabCombiner, showing the advantages of our approach. The method can be applied using M2S (Match 2 Sets), a free, open-source MATLAB toolbox, available at https://github.com/rjdossan/M2S.

## Introduction

Metabolomics has emerged as a powerful tool in biomedical and biological research.^1^ Recent advances in high-throughput liquid chromatography–mass spectrometry (LC–MS) make it possible to perform high-resolution analysis of small molecules in biofluids (e.g., plasma, urine, etc.) from thousands of participants in large-scale epidemiological and clinical research studies.^2^ Despite advances, there remain challenges in large-scale LC–MS metabolomics that need to be resolved to realize its full potential. One challenge is to combine or compare datasets generated from different analytical methods, instruments, software, as well as from different batches, populations, and sample types.^3^ These factors impact a variety of variables including retention times, *m*/*z* ratios, ionization efficiency, adduct formation, detector sensitivity, number of peaks detected, etc., complicating matching of spectral features from one run to another. This is especially challenging in the setting of untargeted metabolomic profiling where there are thousands of spectral features, even within a single run, whose chemical identity is not known. Many packages in the public domain can be used to align features across samples, but not many do that across datasets.

Work related to this subject has focused on linear or nonlinear retention time adjustments to perform retention time alignment of spectral peaks across samples.^4^ The meta-analysis software “metaXCMS”^5^ combines peak lists from multiple datasets but uses simple thresholds for retention time and *m*/*z*, which may not fully capture nonlinear variation between datasets. In 2016, Ganna and collaborators reported that when trying to match their own large cohorts, they could not find any appropriate method in the literature,^6^ as the closest ones were designed to match samples, not datasets. Since then, information about the peak shape, run order, or clustering of chromatogram(s) has been used to improve retention time alignment or matching of spectral features across different samples in a dataset.^7^ Most of these strategies require access to the raw spectrometric data.^8^ The software “metabCombiner”^9^ robustly models the retention time shift between two feature lists from different batches or datasets acquired from the same biofluid type, depending on the feature intensities of the datasets to be highly correlated. This software does not model the systematic shift in *m*/*z* that may happen from one dataset to another. Additionally, it has limited visualizations, which otherwise could help choose the analysis parameters, guide the analyst, and reveal the quality of the results.

Here, we describe a method to address the problem of finding correspondence between datasets. As it only requires the features’ retention time, *m*/*z*, and (optionally) feature intensity values (RT, MZ, FI) averaged across samples, it is simple to use and potentially applicable in the widest range of situations. It can be deployed, for example, where datasets were processed using different software, validation studies were done at a later time, data were acquired/processed by different labs, or several batches were acquired within the same experiment, among others. We apply it to biological and synthetic datasets, proposing various orthogonal validation schemes as evidence of good matching, despite the inherent difficulty of validating matches without annotations. An accompanying toolbox with examples is available, in the widely used, highly interactive, graphically capable MATLAB environment, which can be deployed for its practical application. The method, as applied with this package, does not have high computer memory requirements and the execution is rapid, taking less than a minute using default settings on a regular desktop computer to match datasets with around 6000 features. The toolbox is flexible to accommodate disparate dataset matching, with detailed visualizations that help guide the analyst through the process and lend confidence to the final matching results. We believe the method and associated software will aid the integration of untargeted LC–MS metabolomics data in a wide variety of applications such as batch combination, discovery validation, and multicohort integration.

## Methods

### Method Assumptions

The method assumes that retention times of the same metabolomic features in both datasets may present a nonlinear inter-dataset shift but are still correlated (Figure S1); the elution order of features in each dataset does not need to be the same. Similarly, the *m*/*z* inter-dataset shift is also modeled and the order of *m*/*z* values may not be the same in both datasets (e.g., in the case of peak swapping due to mass errors). Here, we use the median to summarize the average RT, MZ, and FI of each peak across all of the samples of a dataset, although any consistent summary statistic (e.g., mean) could be used. In general, the median value for RT and *m*/*z* is supplied by the processing software, while FI can be calculated from the sample intensity data. If the median feature intensities (as log_10_FI) in both sets are correlated (e.g., for samples from similar populations and biofluids), then log_10_FI can also be used for matching (see Supporting Information—Use of FI for Matching). For best results (see Supporting Information—Procedure Notes), the same adduct and isotopic species should be expected, features known to belong to the same metabolite (e.g., isotopologues, adducts) should not have been aggregated (the aggregate feature may not be the same in both sets), and appropriate quality control of features should have been performed.^10^ The larger the number of metabolites present in both datasets the better, thus ideally the sample material, extraction, and analytical methods should be the same. More matching issues will arise with increased inter-dataset (RT, MZ, FI) dissimilarity.

### Workflow

**Figure 1:**
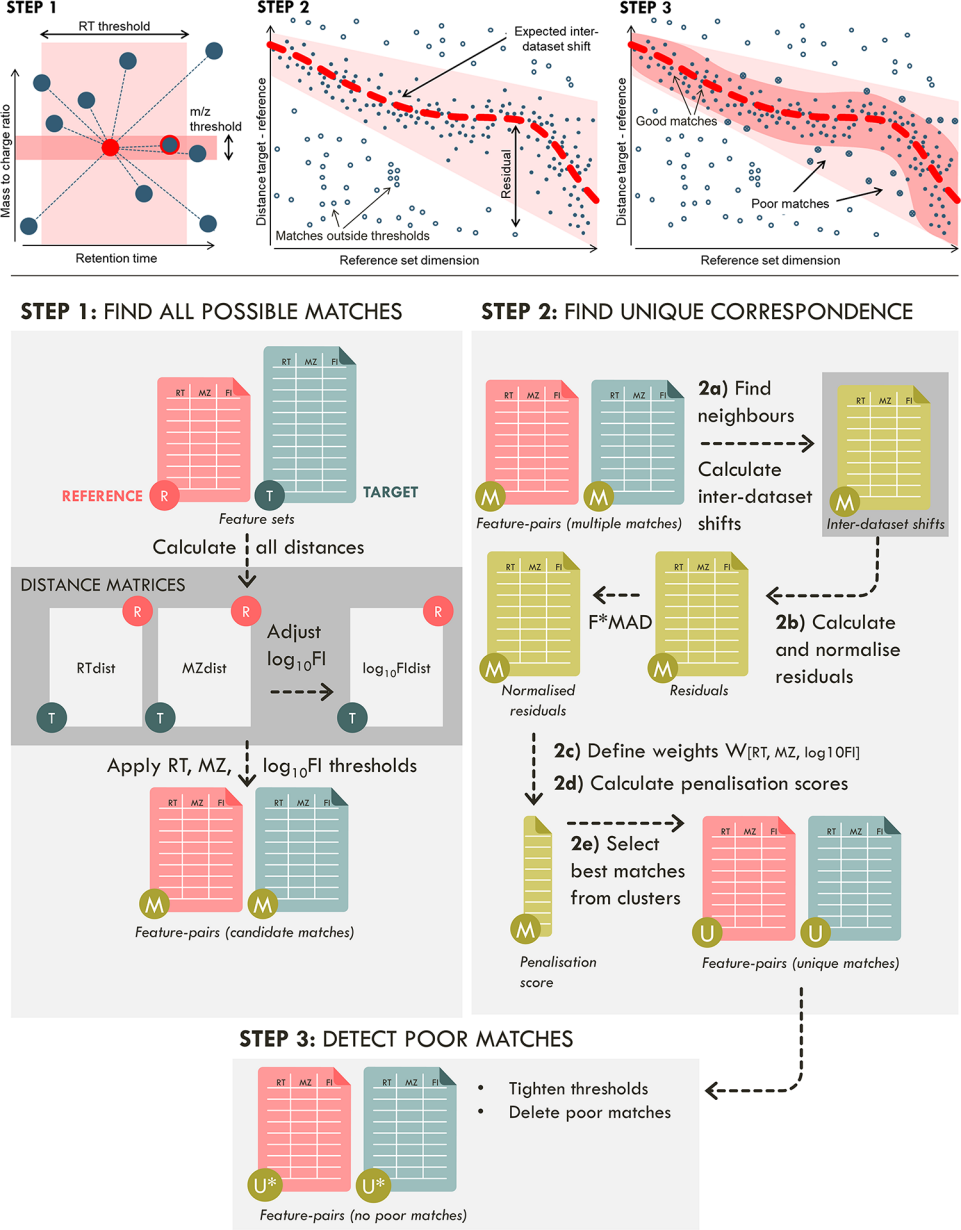
Method workflow: (top) overview of the approach; (bottom) matrices calculated at each step; Step 1: Distances between all features are calculated (RTdist, MZdist, log_10_FIdist) and linear thresholds set in all dimensions, finding “M” candidate matches between feature sets; Step 2: Find one-to-one feature correspondence: 2a: The expected inter-dataset shifts are modeled using neighbor consensus; 2b: residuals can then be obtained for each candidate match, normalized; 2c and 2d: transformed into single-value penalization scores; 2e: these are used to define feature-pair matrices containing only “U” unique matches. Step 3. A nonlinear tightening of thresholds is applied to filter out poor matches far from the inter-dataset shifts, yielding “U*” unique matches.

**Figure 2:**
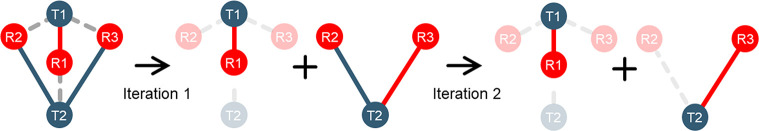
Selection of best matches from multiple candidates, showing decomposition of a cluster with three reference (R) and two target (T) features, as well as connecting lines representing six candidate matches. Red matches (edges) have the lowest penalization score for each cluster at each iteration and are selected. Dashed lines are conflicting matches also containing the best-matched feature and thus are discarded. Blue lines are matches that initially are not the best but are not conflicting with the best match; thus, they can still be chosen in later iterations. In this case, two matches are formed from the original cluster after two iterations (R1–T1 and R3–T2).

**Figure 3:**
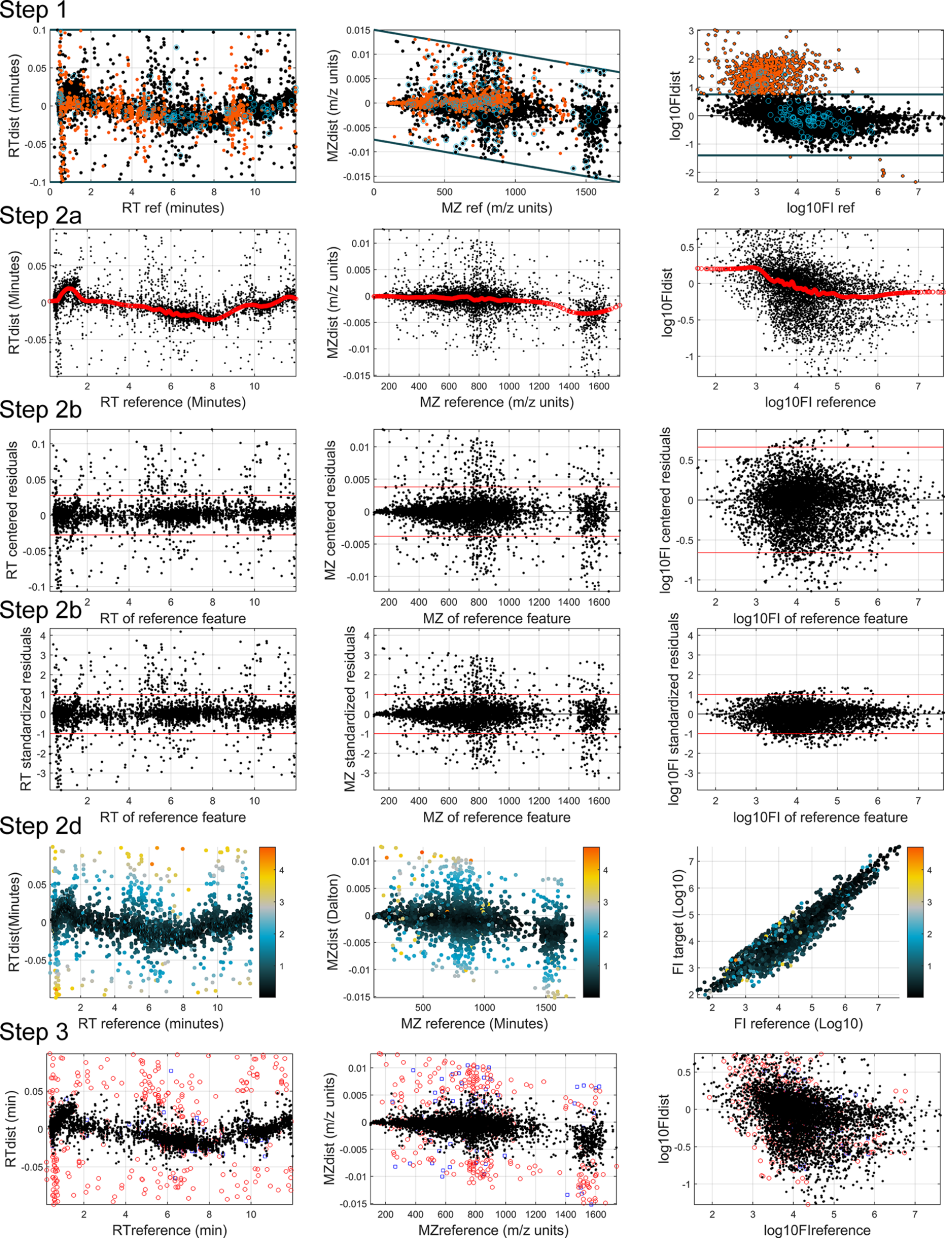
Summary of the data at each step of the workflow. Row 1: (Step 1) Inter-dataset distances for matched features in the (RT, MZ, log_10_FI) domains. Black dots are unique matches, blue circles are matches in clusters, and orange dots are matches outside the log_10_FI threshold limits. Row 2: (Step 2a) Black dots are the same as in Row 1, red circles are expected values at the (RT, MZ, log_10_FI) of the reference feature in the match. Row 3: (Step 2b) Residuals of the expected values. Row 4: (additional Step 2b) Normalized residuals obtained by dividing by the threshold point at their median + 3 × MAD. Row 5: (Step 2d) After defining weights *W* = [1,1,0.2] (Step 2c, not shown) penalization scores are obtained and used to color the same plots as in Row 1 (RT and MZ) and the comparison of log_10_FI of target and reference. Penalization scores are used (Step 2e, not shown) to decide the best match in clusters with multiple matches. Row 6: (Step 3) Tightening of thresholds used to define poor matches using the method “scores” at the threshold limit of median + 3 × MAD. Matches (part of clusters) previously discarded in blue, poor matches in red, and good matches in black.

The workflow is presented in Figure 1, with details in Figure 2 and an example in Figure 3. The method requires only two independent feature sets (RT, MZ, FI) as inputs and follows a three-step process. The feature sets are referred to as reference and target, and calculations and plots are made in relation to the reference dataset.

#### Step 1: Find All Possible Matches

The inter-dataset distances RTdist_tr_, MZdist_tr_ (in Daltons), and log_10_FIdist_tr_ (in log_10_ feature intensity units) between two features are obtained simply by subtraction of reference feature *r* from the target feature *t*. Matches are reference-target feature-pairs whose inter-dataset distances are smaller than the respective thresholds (Figure 1, top row). Features can be involved only in one match or in multiple matches. Matches can be unique (between features that only have one match), or part of clusters of features in multiple matches (where features in one set match two or more in the other). To minimize the number of clusters with multiple matches, the initial thresholds should be as small as possible. Thresholds are absolute or relative values (horizontal and diagonal lines respectively) initially user-defined according to (RT, MZ, log_10_FI) difference patterns observed in specific plots, such as in top row center plot of Figure 1 and top row of Figure 3. Initially, *M* candidate matches are found, some unique and some in clusters. The difficulty in matching two datasets depends on the (RT, MZ, log_10_FI) inter-dataset dissimilarity, which has an impact on the thresholds needed.

#### Step 2: Find Unique Correspondence

To achieve useful matching, inter-dataset feature correspondence must be unique. Nonunique correspondence arises when the threshold limits are not tight enough to avoid clusters with multiple matching. The decision of which match to choose among several in a cluster of multiple matches is based on the comparison of a penalization score calculated for each candidate match in the cluster. The penalization score is built using (normalized) residuals in each dimension and increases with the distance between the match and the expected inter-dataset shift in figures such as the top row of Figure 1. Importantly, note that the expected inter-dataset shift is nonlinear with respect to the reference feature’s value (*x*-axis), while the initial thresholds are linear. The best matches, with inter-dataset distance close to the expected inter-dataset shift (small residuals), have lower penalization scores.

#### Step 2a: Define Inter-Dataset Shift Using Feature Neighbors

Consider the dimension RT (the same applies for MZ and FI). For a specific match *m*, the expected inter-dataset shift RTdist_expected(m)_ at the RT of its reference feature (RT_ref_) is defined by the median RT shift of its *k* nearest neighbors in the MZ vs RT dimensions. We propose two methods to determine neighbors (details in Supporting Information Methods: Define Neighbors). The “cross” method in which *k* neighbors are separately calculated in each dimension (neighbors may be different in each of the dimensions) allows the calculation of a smoothed curve yielding the same inter-dataset shift for all matches at the same RT_ref_. The “circle” method, in which *k* neighbors are found using (normalized) Euclidean distance for RT and MZ (but not FI) simultaneously yields different neighbor features for the same RT_ref_, thus allowing matches with the same RT_ref_ but at different MZ_ref_ values to have distinct shifts (see Figure S6) and vice-versa. This may be advantageous, e.g., if MZ differentiates metabolites with different physicochemical properties eluting at different RTs in the second dataset.

#### Step 2b: Calculate and Normalize Residuals

Consider a match *m* and the inter-dataset distance between its two features in the RT domain (RTdist_m_). The residual distance for that match is the difference (ΔRTdist_(m)_) between RTdist_m_ and the expected inter-dataset shift RTdist_expected(m)_ at the RT_ref_ of the match. The residuals for each dimension have different units (minutes, Daltons, log_10_FI units) and are therefore normalized, dividing by a threshold defined as the median of the residuals plus a factor (*F* = 3) times their median absolute deviation (MAD), as in eq 1 (here, *x* represents the residuals in one of the dimensions, e.g., *x* = ΔRTdist).
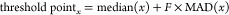
1After this adjustment, the residual value for all dimensions is 1 at the defined residuals’ threshold values, allowing combination into a single score.

#### Step 2c: Define Weights for Each Dimension’s Residuals

The penalization score uses a weighted combination of the normalized residuals. In the simplest case, the weights *W*_RT,MZ,FI_ can be the same in all dimensions ([1, 1, 1]). However, in many datasets, the FI values are not comparable; thus, the FI weight can be manually adjusted by inspection of residual plots such as in row 4 of Figure 3. For cases where FI is not relevant for matching, set *W*_FI_ = 0.

#### Step 2d: Calculate Penalization Scores

The joint penalization score for each candidate match *m* is simply defined as the square root of the weighted sum of squares of the normalized residuals, as in eq 2
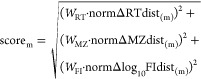
2

#### Step 2e: Select Best Matches in Multiple Match Clusters

Consider the candidate matches as a network (such as in Example S1, Figure S13), with features as nodes and matches as edges. Features with unique matching constitute clusters with two nodes but features in multiple matches form clusters with more than two nodes, necessarily containing wrong matches. To find the best match (minimum penalization score) within a cluster with more than two nodes, an algorithm recursively selects the best match (see Figure 2) until unique matching is achieved for all its features.

#### Step 3: Detect Poor Matches (Tighten Thresholds)

Poor matches are the ones that although being unique (not in a multiple match cluster) are very far away from the expected inter-dataset shift and may have been accidentally matched due to the use of large initial thresholds. Notice that the larger the thresholds, the more spurious poor matches will happen. The detection of poor matches (an optional step) can be done by redefining nonlinear RT, MZ, and log_10_FI thresholds (see Supporting Information Methods/Detect Poor Matches) in a similar manner to the residuals’ normalization described in Step 2b.

### Method Validation

There is no absolute way of validating all results of matching since annotations are not available for all features. Nevertheless, multiple different, orthogonal strategies provide evidence of matching quality. We propose (1) comparing manual annotations in the two datasets where they are available, (2) comparing the correlation of FI values in the two datasets, (3) comparing the correlation of matched features to known covariates, (4) in clusters, comparing the number of samples in which the matched features were detected, (5) evaluating the number of multiple match clusters vs unique matches, and (6) for the features in a match, evaluating the number of common highly correlated features. These strategies can also be employed by users to evaluate their matching results.

## Experimental Section

### Data

#### Dataset 1

See Supporting Information—LC–MS datasets details, and complete analysis in Example S1. Our primary example comprises data from the MESA^11^ and Rotterdam^12^ studies acquired on the same instrument, utilizing reversed-phase ultraperformance LC–MS with electrospray ionization in positive mode (RP UPLC MS ESI+). The MESA cohort was used as a reference (in total, 2656 samples, of which 1969 were biological samples, 10909 features). The Rotterdam dataset was used as the target (totaling 1057 samples, of which 739 were biological samples, 15 267 features). In each dataset, the remaining samples consisted of calibration and quality control (QC) samples. Both datasets were (separately) peak-picked using XCMS,^13^ yielding a table of samples (rows) by features (columns). No de-isotoping or adduct clustering was applied. Quality control (QC) of features was applied, in which only features eluting before 12 min were retained, and a QC dilution series^14^ was used to eliminate features whose response to dilution was worse than a threshold of *R*^2^ <0.7. After QC, the MESA dataset contained 10 427 features and the Rotterdam dataset contained 14 097. To characterize each feature, the medians of RT, *m*/*z*, and FI across all samples were used.

#### Dataset 2

See Supporting Information—LC–MS datasets details, and complete analysis in Example S2. The same MESA and Rotterdam samples were analyzed in negative ionization mode (RP UPLC MS ESI−). Initially, there were 15 978 features in MESA and 13 030 in Rotterdam. Similar quality control of features was applied as for dataset 1, with the exception that retention time trimming only selected features between 0.45 and 9.5 min. After QC, the MESA dataset contained 6793 features and the Rotterdam dataset contained 6315.

#### Dataset 3

See analysis in Example S3. Synthetic data were produced from Dataset 2 by adding systematic and random variability to the samples as detailed in Example S3.

#### Datasets 4, 5, and 6

See analysis in Examples S4–S6. Paired datasets with varied characteristics: different experiments; chromatographic columns (reversed-phase, HILIC); instruments; processing software; large RT and MZ differences; different biofluids. These are used to compare the results of M2S and an existing method, metabCombiner.

### Metabolite Annotation

Features, including adducts and isotopologues, were annotated to confidence level 2 according to the Metabolomics Standards Initiative.^15^ This was done matching accurate mass, isotopic distributions, and fragmentation spectra (from MS^E^ all-ion fragmentation scans) to reference data from an in-house standards database and online databases LIPID MAPS,^16^ METLIN,^17^ HMDB,^18^ GNPS,^19^ and MassBank.^20^

## Results

### Matching of Dataset 1

#### Step 1: Match All Variables within Thresholds

Large thresholds (RT: 1 min; MZ: 0.025 Da) were applied for an overview of RT, MZ, log_10_FI inter-dataset shifts, and a set of candidate matches obtained, as shown in Example S1, Figure 2. User-defined thresholds (see Example S1) were manually adjusted for each of the three dimensions guided by visual inspection of the plots in row 1 of Figure 3. This yielded 5426 matches, including those in 61 clusters of multiple matches, as well as 5303 unique matches (see Table S1 and network in Example S1, Figure S13).

#### Step 2: Find Unique Correspondence

After defining the number of neighbors using the “cross” method with 1% of the total number of features in the reference dataset (see Supporting Information “Methods/Define Neighbors”), the expected inter-dataset shift was robustly determined (row 2, Figure 3) and the residuals obtained (row 3, Figure 3).

A threshold of median + 3 × MAD was used to normalize the residuals in each of the dimensions. The weights were then defined as *W*_RT,MZ,FI_ = [1, 1, 0.2] to give RT and MZ equal weight, allowing FI to significantly influence the penalization score of matches only if its difference is very large. The normalized residuals can be seen in row 4 of Figure 3 and all residuals (centered, normalized, and weighted) can be seen in Figures S9–S11. The penalization scores were then calculated as the weighted sum of squares of the residuals according to eq 1 and can be visualized as the color gradient in row 5 of Figure 3. After selection of best matches by comparison of penalty scores, a total of 5365 unique matches are found.

#### Step 3: Find Poor Matches (Tighten Thresholds)

The original linear thresholds do not follow the curve typically observed in the inter-dataset distance plots (e.g., 2nd row of Figure 3). Therefore, new nonlinear thresholds are defined (in this case, using the “scores” method at median + 3 × MAD to remove poor matches located far from the inter-dataset shift trends), as shown in row 6 of Figure 3, ending up with 4953 unique matches.

### Method Validation

#### Comparison of Metabolite Annotations

**Figure 4:**
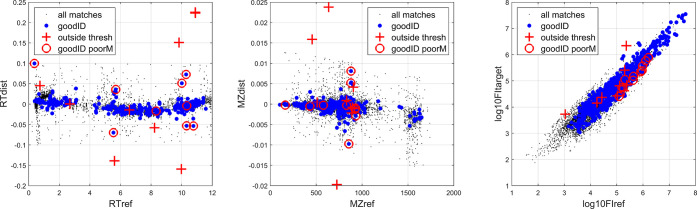
Inter-dataset distances after initial candidate matching (Step 1), with results of validation using annotated features. Black dots are matches within thresholds; blue dots are matches with identical annotations; nine red crosses are annotated matches outside of the initial thresholds; 10 red circles are annotated matches wrongly considered poor matches.

We evaluated if the annotations were the same for matched features, finding very good agreement (Figure 4 and Table 1). There were 604 annotations in common in the initial data of both reference and target datasets. After step 1, we noticed that nine annotations could not be matched across datasets as they were outside the initial thresholds. Otherwise, step 1 found 5426 matches (average of 44% of initial features). After step 2, 5365 unique matches were found, and all of the remaining 595 features were correctly matched. In step 3, when detecting poor matches, 412 matches are deleted, among which 10 annotated ones were correctly matched. As a summary of results, 4953 matches were found, of which 585/604 (96.8%) annotated features were correctly matched and 19 (3.1%) annotated features were not found within thresholds (in steps 1 and 3). Importantly, all of the 585 annotated features within thresholds were matched correctly.

**Table 1:** Matches and Annotations at Each Step s1–s3

stage and results	annotations	matches^a^
initial data	604	(10 427/14 097)
matches outside thresh		9
after initial matches (s1)	595	5426
after unique matches (s2)	595	5365
correct ID matches	595	
wrong ID matches	0	
after poor matches (s3)		4953
final correct ID matches	585	
final wrong^b^ matches	19	
poor matches	10	412
with correct ID	10	
with wrong ID	0	

a Numbers refer to matches, and when in parenthesis refer to features.

b Wrong ID or outside threshold.

#### Comparison of FI

The composition of blood in healthy subjects is highly regulated, and thus the average concentration of metabolites should be highly correlated between datasets. Although from different populations, the sample type, extraction, injection, and peak-picking methods were similar, and we observe that the datasets show good agreement in log_10_FI for most matched features (Figure 4, right).

#### Comparison of Associations to Covariates

We assumed the direction of association of metabolites with specific covariates should be similar in both datasets. We tested this using covariates age, BMI (both by linear regression), and gender (median log_10_ fold change and t-test), for which distributions can be found in Example S1, Figure S18, and all associations in Example S1, Figure S19, and Table S3. We evaluated this approach for: (A) all features; (B) only for matches with statistically significant coefficient/*t*-test at α = 0.05; (C) as B, but controlling for false discovery rate (Benjamini–Hochberg at FDR = 0.05); and (D) as B, but controlling for family-wise error rate (Bonferroni, α = 0.05). Briefly, the three covariates show a level of agreement close to 60% when using all variables (as only a minority of features correlate with these covariates), increasing to close to 100% for more stringent thresholds such as FDR and Bonferroni, suggesting good agreement and correct matching.

#### Evaluation of Match Selection in Multiple Match Clusters

The datasets were processed using XCMS,^13^ which outputs an “npeaks” variable, roughly indicating in how many samples the feature was found. In the following, we assume that features detected in a higher number of samples have larger signal-to-noise ratios and better quality; thus, correctly matched features should be detected in more samples than incorrect matches. For each match in a cluster of multiple matches, we computed the “npeaks” difference of [features in selected matches minus those in discarded matches] (Example S1, Figure S20). The reference dataset contains 1958/2639 biological/total samples, respectively, and the target dataset contains 814/1178 (datasets contain QC samples at time of peak picking). A high proportion of “npeaks” differences are positive (50 in 60, or 83% in both reference and target), suggesting that the correct match was usually selected from each cluster.

#### Evaluation of Number of Multiple Match Clusters

If the number of multiple match clusters is small, this suggests that most features are unique, and therefore well matched at plausible (RT, MZ, FI) distances. After all matches within initial thresholds are found, the network of Example S1, Figure S13, and Table S1 show only 61 (1.1%) clusters, while 5303 (98.9%) are uniquely matched, limiting the probability of errors coming from best-in-cluster decisions.

#### Evaluation of the Number of Features Highly Correlated with the Matched Features

**Figure 5:**
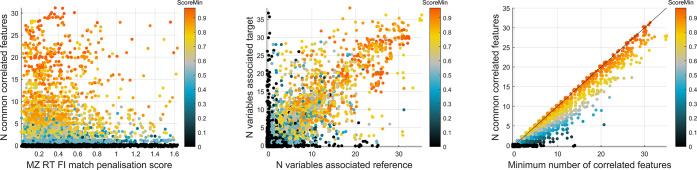
(Left) Number of common features^a^ highly correlated^b^ with each matched feature vs penalty scores used in the matching method. The lower the penalty score, the higher the number of common correlated features. (Center) Number of features highly associated (not necessarily common) with each matched feature in target vs reference. (Right) Number of common correlated features vs the minimum number of correlated features (not necessarily common) between the reference or target datasets. All plots are colored by “patternScore” obtained by the ratio common/(minimum +1). ^a^ Only features surviving removal of poor matches. ^b^ Spearman correlation >0.7 and ΔRT <0.25 s.

As there was no feature aggregation, these datasets should contain a pattern of isotopologues and adducts for each metabolite. These features in each pattern will be highly correlated to each other (across the samples), and we expect to see a similar pattern for matched features in each dataset. For the reference and target feature in each match, we selected all features in the same dataset within a small RT window (<0.25 s) and whose intensities were highly correlated (Spearman correlation >0.7), denoting these sets as *R* and *T* respectively. To express the similarity of the patterns, we then calculated the “patternScore” for each match as the ratio of common features to the minimum set size, adjusted by one to avoid division by zero, as seen in eq 3
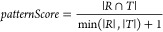
3where |*X*| denotes the size of set *X*. Figure 5 (left) shows that for lower penalty scores, there is a trend to higher number of common correlated features. Figure 5 (center) shows good agreement between the total number of correlated features in the two datasets, while Figure 5 (right) shows that the number of common features is close to the maximum possible. This represents good evidence of the quality of the penalty scores method for choosing the best matches and for the quality of the matches themselves.

### Matching of Other Datasets

The result of matching Dataset 2, comprising sera from the same two cohorts analyzed in negative mode, is presented in Example S2. Even with a larger, more curved inter-dataset shift in RT the method performs very well according to the validation strategies. From an initial total of 6793/6315 unmatched features in reference and target, respectively, 2486 could be uniquely matched, which became 2324 after removing poor matches. From the initial 87 annotated features in both sets, there were 3 that matched outside of the initial thresholds. From the 84 that matched uniquely, 82 were correctly matched (same annotation) while 2 were not. After deleting poor matches, 3 of the correctly matched were removed, ending with 79 (90.8%) annotations correctly and 8 (9.2%) wrongly matched or outside thresholds.

The analysis of simulated data (Example S3) confirmed that the method appropriately finds the number of expected matches. The initial datasets contained 4755 features, and 2717 matches were expected. The method found a total of 2798 candidate matches, which contained 2728 unique matches (100% of the true matches plus 11 false positives). Notice that there are more unique matches than expected ones, as additional matches not accounted for by the dataset design may happen by chance. After deleting poor matches, the number of matches was 2682 (2682/2717 = 98.71%).

Finally, the analysis of three datasets in Examples S4–S6 shows the application of our method to various dataset pairs and compares the results with metabCombiner.

## Discussion

The success of using scores to refine clusters of multiple matches into single matches depends on robust modeling of expected inter-dataset shifts of RT, MZ, and log_10_FI. Notice that while modeling inter-dataset MZ shifts may not be relevant in the context of matching peaks across samples, the same is not true while matching datasets. In case there is a large systematic shift in MZ—observed between many datasets—it is of utmost importance for that shift to be modeled to choose the most probable matches from clusters of multiple matches. As no number fits best all datasets, initially large thresholds should be used to ensure that inter-dataset shifts—and matches near them—are captured. Then, we propose that absolute and relative linear threshold limits are set appropriately, guided by visual inspection of the distance plots resulting from the initial matching. The difficulty of choosing the appropriate initial thresholds is demonstrated in both example datasets, as the number of annotated features that escape matching by being outside those thresholds is 9 (1.5%) and 3 (3.4%) in examples 1 and 2, respectively.

The inter-dataset shifts are in general nonlinear and nonunique for matches at the same, e.g., RT, and hence a robust method to define the shift locally rather than globally is warranted. Our method uses neighbor consensus distances to calculate inter-dataset shift trends in each dimension and only allows single-match features as neighbors to increase robustness. Distances to the inter-dataset shift trends in each dimension can then be obtained (residuals), which after weighting allow the calculation of a penalization score for each match. A recursive methodology selecting at each iteration the best match in each cluster with multiple matches allows the selection of one or more matches from each of the clusters.

For the method to work, there must be some correlation between the retention times of the two experiments, even if nonlinear. Proper quality control of features prior to matching avoids poor matches, while robustness improves when both samples and/or methods are similar. The presence of similar adducts and isotopologues increases both the number of matches and the possibility of mismatches. But aggregating features (e.g., by de-isotoping) may complicate the inter-dataset feature matching as different *m*/*z* values may be chosen to represent the same metabolite in the two datasets. Even when available, the feature intensity may not be comparable in both datasets and cannot always be used to help define correspondence.

The incorrect selection of unique matches from clusters (in step 2) is undesirable, as it prevents true matches between the correct features. But poor matches (in step 3), though undesirable, do not jeopardize a correct matching. Nevertheless, it may be of interest to remove them as they increase the number of features in a matched dataset, inflating multiple testing corrections during statistical analysis, thus reducing the chance of correct discoveries. Due to abnormally large shifts of some metabolomic features, this final/optional step may inadvertently delete some correct matches. In Example 1, there were 10 (1.6%) correctly annotated matches that were considered as poor, with 3 (3.6%) in Example 2. The decision on when to use step 3 to detect and delete poor matches rests with the analyst, as it may be relevant to be more liberal or stricter, depending on the application.

In the main example, a cluster of features at different FI in each set (containing none of the 604 annotated features) could be the result of column bleeding, thus appearing in both sets. This cluster was removed by setting a tighter upper threshold in the FI domain in the initial candidate matching (right plot in row 1, Figure 3), showing the versatility of our method.

Using different validation strategies, we collected evidence of very good performance for the cases presented. The comparison of annotated features is the most accurate way of validating the results, and while being limited by the number of annotations in our datasets, it showed excellent performance. The comparison of log10FI between the matched features also suggested that a good result was reached (Figure 3, row 5, right plot; Figure 4, righthand plot). Evidence of good matching was also obtained from the comparison of association to covariates, with very good agreement for the matched features. The comparison of the “npeaks” allowed us to assess the quality of our refinement of multiple match clusters and suggested a majority of correct choices. The evaluation of multiple match clusters suggests there is not much room for mistakes after initial matching if the ratio of unique matches/clusters is high, as the mismatching error when deciding unique matches is very low, with 595 (100%) and 82 (97.6%) annotated features correctly matched in Examples 1 and 2. Finally, validating using highly correlated features gathered strong evidence of good quality matching, particularly in the main example.

The analysis of three diverse datasets and comparison with an alternative method, metabCombiner (Examples S4–S6), revealed the power of the M2S method to match nonannotated datasets. As expected, metabCombiner produced robust RT modeling between the same biofluid datasets. But Examples S4 and S5 show how important it is to robustly model not only RT but also the systematic shift in MZ, otherwise risking choosing the wrong matches from multiple match clusters. Additionally, the examples showed the practical difficulties of defining thresholds, setting weights, and understanding the quality of the results without proper visualizations in metabCombiner, in contrast to the highly plot-capable M2S. Moreover, we show the ability of M2S to match between different biofluids (serum vs urine) in Example S6. Other aspects of M2S plasticity were also demonstrated in the Supplementary Examples, as they comprised different datasets acquired by different groups, with different instruments, chromatographic columns (reversed-phase, HILIC), processing software (XCMS, Progenesis QI, MassHunter Workstation suite), large RT and MZ differences, and different biofluids (plasma, serum, urine).

This method contains some positive ideas and concepts that work synergistically to reach its objectives. The visualizations on which the method is based are powerful, guiding the analysis (e.g distance plots to model inter-dataset shift trends) and lending confidence to the results (e.g., network plots). Setting initial noncentered and asymmetric relative thresholds in the selected dimensions (RT/MZ/FI) reduces the number of multiple match clusters, thus resulting in a more precise modeling of the inter-dataset shift trends. For inter-dataset shift modeling, the use of an averaged value of each variable, e.g., RTdist, to represent the expected RTdist for matched features is robust. It is remarkable that allowing nonunique values of RTdist for features at the same RT is also a successful strategy (the method “circle”; the same applies for the other dimensions).

In challenging cases it is not straightforward to match the two datasets: it may be difficult to find and model the inter-dataset trends; the (RT, MZ) range of the two datasets may need to be adjusted prior to matching, so they have similar minimum and maximum values. When forced to set large thresholds due to large dataset dissimilarities, there may be too many multiple match clusters. It may not be easy to decide if the matching is acceptable when matching very different datasets (e.g., human plasma and cerebrospinal fluid).

In developing our approach, we deliberately avoided incorporating strategies that would limit its applicability. For example, we did not use raw spectra as these are often unavailable, and the method does not depend primarily on high FI correlation as in ref (9). Two methods were used for validation rather than as part of the matching: within-set correlation patterns are informative but can be different in different datasets, while correlations of features to covariates (e.g., age) also contain useful matching information but can vary widely and were therefore not used in the algorithm.

Finally, our method can be used for more than two datasets by matching all to the same reference, though this sequential strategy increases the probability of matching errors. Methods simultaneously matching features of multiple datasets should yield better results.

## Conclusions

We have presented a method to find feature correspondence between two untargeted LC–MS datasets using only RT, MZ, and FI. Its simplicity and ease of use confer versatility, and integrated visualizations help guide the analysis, allowing its application to a wide range of situations. The method returns two datasets with feature correspondence increasing statistical power or facilitating discovery/validation studies. Software is freely available and was demonstrated on an extensively annotated cohort. Results of six orthogonal validation strategies suggest that the results are of very high quality. Analysis of three paired datasets with diverse characteristics was also showcased, in which M2S showed important advantages over an alternative method, metabCombiner.

## Supporting Information Available

Details of LC–MS datasets, procedure notes, supplementary figures, method details, workflow, and results of six examples (PDF)
