# Supplementary material for: Finding Correspondence between Metabolomic Features in Untargeted Liquid Chromatography–Mass Spectrometry Metabolomics Datasets
Source: Anal Chem. 2022 Mar 31;94(14):5493–503. doi: 10.1021/acs.analchem.1c03592 (PMC9008693; doi:10.1021/acs.analchem.1c03592)
Supplement: Supplementary file 1 — ac1c03592_si_001.pdf [file ac1c03592_si_001.pdf]

Supplementary Information (SI)

Table of Contents

Supplementary Information (SI) 1

Finding correspondence between metabolomic features in untargeted liquid chromatography - mass spectrometry metabolomics datasets 3

1. Details of LC-MS datasets 3

1.1. Example S1: MESA serum LPOS vs Rotterdam serum LPOS and Example S2: MESA serum LNEG vs Rotterdam serum LNEG 3

1.2. Example S4: AD plasma LPOS vs Airwave plasma LPOS 4

1.3. Example S5: Airwave plasma HPOS vs MESA serum HPOS 5

1.4. Example S6: MESA serum HPOS vs Airwave urine HPOS 6

2. Procedure notes 6

2.1. Reference and target datasets 7

2.2. Use of FI for matching 7

2.3. Metabolomic feature aggregation 7

2.4. Metabolomic feature quality control 7

3. Supplementary figures 7

4. Methods 8

4.1. Calculation of all distances between features in the two datasets (part of step 1) 8

4.2. Definition of initial matching thresholds (part of step 1) 8

4.3. Adjust FI of target to FI of reference (part of step 1) 9

4.4. Define neighbours (part of step 2a) 9

4.5. Step 2a: Define inter-dataset shift using feature neighbours 12

4.6. Step 2b: Calculate and normalise residuals 12

4.7. Step 2c: Define weights for each dimension's residuals 13

4.8. Step 2d: Calculate penalisation scores 13

4.9. Step 2e: Select best matches in multiple-match clusters 13

4.10. Step 3: Detect poor matches (tighten thresholds) 14

5. Example S1: MESA serum LPOS vs Rotterdam serum LPOS 15

5.1. Matching procedure 15

5.2. Validation 23

6. Example S2: MESA serum LNEG vs Rotterdam serum LNEG 28

6.1. Matching procedure 28

6.2. Validation 36

7. Example S3: Step-by-step analysis of synthetic data ... 39
7.1. Data ... 39
7.2. Procedure ... 40
8. Example S4: AD plasma LPOS vs Airwave plasma LPOS ... 44
8.1. Matching using metabCombiner ... 44
8.2. Matching using M2S ... 46
8.3. Comparison of results of metabCombiner and M2S ... 51
9. Example S5: Airwave plasma HPOS vs MESA serum HPOS ... 53
9.1. Matching using metabCombiner ... 53
9.2. Matching using M2S ... 54
9.3. Comparison of results of metabCombiner and M2S ... 59
10. Example S6: MESA serum HPOS vs Airwave urine HPOS ... 61
10.1. Matching using metabCombiner ... 61
10.2. Matching using M2S ... 62
10.3. Comparison of results of metabCombiner and M2S ... 67

Finding correspondence between metabolomic features in untargeted liquid chromatography - mass spectrometry metabolomics datasets

Rui Climaco Pinto*[1, 2], Ibrahim Karaman[1, 2], Matthew R. Lewis[3, 4], Jenny Hällqvist[5, 6,] Manuja Kaluarachchi[2, 7], Gonçalo Graça[7], Elena Chekmeneva[3, 4], Brenan Durainayagam[1,2], Mohsen Ghanbari[8], M. Arfan Ikram[8], Henrik Zetterberg[9, 10, 11, 12], Julian Griffin[7], Paul Elliott[1, 2], Ioanna Tzoulaki[1, 13], Abbas Dehghan[1, 2, 8], David Herrington[14], Timothy Ebbels[*7]

*Corresponding authors: r.pinto@imperial.ac.uk; t.ebbels@imperial.ac.uk

[1] Dept. Epidemiology and Biostatistics, MRC-PHE Centre for Environment and Health, School of Public Health, Imperial College London, London, UK; [2]UK Dementia Research Institute, Imperial College London, London, UK, [3]MRC National Phenome Centre, Dept. Metabolism, Digestion and Reproduction, Imperial College London, London, UK; [4]Section of Bioanalytical Chemistry, Department of Metabolism, Digestion and Reproduction, Imperial College London, London, UK; [5]Centre for Translational Omics, Great Ormond Street Hospital, University College London, London, UK; [6]Dept. Clinical and Movement Neurosciences, Queen Square Institute of Neurology, University College London; [7]Section of Bioinformatics, Division of Systems Medicine, Dept. Metabolism, Digestion and Reproduction, Imperial College London, London, UK; [8]Dept. Epidemiology, Erasmus University Medical Center, Rotterdam, Netherlands; [9]Dept. Psychiatry and Neurochemistry, Institute of Neuroscience and Physiology, the Sahlgrenska Academy at University of Gothenburg, Mölndal, Sweden; [10]Clinical Neurochemistry Laboratory, Sahlgrenska University Hospital, Mölndal, Sweden; [11]Dept. Neurodegenerative Disease, University College London, Queen Square, London, United Kingdom; [12]UK Dementia Research Institute, University College London, London, United Kingdom; [13]Dept. Hygiene and Epidemiology, University of Ioannina School of Medicine, Ioannina, Greece; [14]Dept. Internal Medicine, Wake Forest School of Medicine, Winston-Salem, NC, USA.

1. Details of LC-MS datasets

1.1. Example S1: MESA serum LPOS vs Rotterdam serum LPOS and Example S2: MESA serum LNEG vs Rotterdam serum LNEG

These datasets were acquired by Metabometrix, a London-based metabolomics service company. The samples were processed and analysed in study batches. Multi-ethnic study of atherosclerosis (MESA) [1] and Rotterdam study [2] human serum samples were thawed, and three parts of cold isopropanol were added to each sample, followed by incubation of 2 h at -20°C, centrifugation, and analysis of the supernatant with a Waters Acquity Ultra Performance LC system coupled to a Xevo G2-S ToF mass spectrometer (Waters Corp., Milford, MA, USA) . The metabolites were separated by reversed-phase ultra-performance liquid chromatography by gradient elution. The mobile phases were composed of a solution of 5 mM Ammonium acetate + 0.05% Acetic acid in a mixture of 25:25:50 proportion of Isopropanol, acetonitrile and ultra-pure water (Mobile phase A); and 5mM Ammonium acetate + 0.05% Acetic acid in a 50:50 mixture of Acetonitrile and Isopropanol (Mobile phase B). After injection of 100 µL sample the chromatography was run at flow rate of 0.6 mL/min using the gradient: 99% A (0-2 min); 70% A (2-11.5 min) and 10% A (11.5-12 min). The MS data was collected separately in positive and negative mode electrospray ionization (RP UPLC MS ESI+/-). The capillary voltage was set to 1.5 kV for positive mode and 1.0 kV for negative mode, cone voltage was 20 V, source temperature was set at 120 °C with a cone gas (nitrogen) flow rate of 50 L/h, a desolvation gas temperature of 600

°C, and a nebulization gas (nitrogen) flow of 1000 L/h. All mass spectral data were collected in centroid mode using a 50-2000 m/z scan range. Lock-mass scans were collected every 30 s to perform mass correction. Leucine Enkephalin (555.2645 amu) (20 µg/L) at a flow rate of 15 µL/min was used for lock-mass correction. The MS^E data acquisition mode was used, in which MS scans are acquired by alternating all-ion fragmentation with no fragmentation [3]. When no fragmentation was employed (odd scans) a low collision energy (4 eV) was used and a high collision energy (ramp (10-30 eV) was used to acquire fragmentation scans (even scans). Only low collision scans MS from both datasets were considered.

Both datasets were separately processed using XCMS [4]. Briefly, peaks were picked using centWave method using the following parameters for UPLC MS ESI+: 15 ppm tolerance, peak width (8, 20), signal-to-noise threshold (snthresh) 10, noise level 300 and prefilter (6, 1000); and for UPLC MS ESI-: 30 ppm tolerance, peak width (5, 20), signal-to-noise threshold (snthresh) 15, noise level 300 and prefilter (6, 1000). After peak grouping, non-linear retention time correction was applied, then missing values were imputed which yielded a table of samples (rows) by features (columns).

Retention time trimming was applied to both MESA and Rotterdam datasets. In the LPOS platform, only features at RT < 12 minutes were selected, while in the LNEG platform only features between 0.45 – 9.5 minutes were selected. Additionally, a set of quality control samples created from the same pooled sample but at different dilution levels were injected, and for each metabolomic feature the linearity of the measured value vs know value was evaluated using robust linear regression. R-squared was used as a goodness-of-fit measure, and all features with R-squared < 0.7 were removed.

1.2. Example S4: AD plasma LPOS vs Airwave plasma LPOS

1.2.1.AD plasma LPOS

This dataset was acquired by Prof. Julian Griffin's group at Imperial College London. The 40 samples were obtained from a group of Alzheimer's patients and control. Briefly, samples were defrosted from -80°C overnight to 4°C. Extraction was performed using a modified Folch extraction, 100 µl plasma was homogenised in 800 µl cold (-20C) $CHCl_3$:MeOH (2:1,v/v). In addition, 20 µl of each sample were collected for a pooled quality control sample (QC). Samples were stored at -20°C for 30 minutes to ensure protein precipitation. Then 400 µL water was added, followed by vortex mixing (2 x 20 s) and centrifugation at 12000 RPM, 4°C, for 15 minutes. The organic lower layer from each sample was transferred to a 2ml glass vial and evaporated to dryness under a stream of nitrogen and stored at -20°C until analysis.

On the day of analysis samples were re-constituted with IPA:ACN:H_2O and each sample spiked with SPLASH LIDIDOMIX Mass Spec Standard (1:300, Avanti Polar Lipids Inc., Alabaster, AL, US). Instrument setup has been described previously [5]. Briefly, the ion-mobility chromatography used an Infinity II UHPLC coupled to an Agilent 6560 IM QTOF MS (Agilent Technologies, Santa Clara, USA) using a reversed-phase ACQUITY CSH C18 column (1.7µm, 2.1 x 100mm, Waters, UK), thermostated at 55°C during analysis. The mobile phase consisted of (A) 10 mM ammonium formate solution in 60% of water and 40% of ACN and (B) 10 mM ammonium formate solution containing 90% of IPA, 10% of ACN, pumped at a flow rate of 400 µL/minute. The gradient was initiated at 60% A, linearly decreased to 50% A over 2 minutes, then decreased to 1% A over 14 minutes before being brought back to initial conditions over 2 minutes.

Raw data was pre-processed with the MassHunter Workstation suite (Agilent Technologies, Santa Clara, USA) to perform mass re-calibration, $^{DT}CCS_{N2}$ re-calibration and deconvolution, yielding a matrix containing all features present across all samples. The data matrix was further processed with KniMet

[6], a pipeline on the Knime analytic platform. Features were filtered based on their presence in blanks and QC samples.

1.2.2. Airwave plasma LPOS

The Airwave health monitoring study [7] was established to study possible health risks from the use of a personal communication device in police and emergency personnel in the United Kingdom. This large epidemiological dataset (~3000 samples) was acquired by the National Phenome Centre at Imperial College London. Airwave utilised lithium heparin plasma samples stored at −80 °C prior to the analysis. The complete sample preparation and acquisition details can be found in [8]. Briefly, samples were thawed at 4°C for 2h, then prepared for lipid analysis by addition of four parts of isopropanol (IPA) containing the mixture of reference standards to one part of the sample for protein precipitation. After mixing to allow protein precipitation and centrifugation for 10mins at 3486×g and 4°C, the supernatant was aliquoted to a 96-well plate for the analysis. Subsequently, 2µL of sample were injected in the chromatographic system using full loop mode. Lipidomic profiling was conducted using a 2.1×100mm BEH C8 column, held at 55°C. Mobile phase A consisted of a 50:25:25 mixture of H_2O:ACN:IPA with 5mm ammonium acetate, 0.05% acetic acid, and 20µM phosphoric acid. Mobile phase B consisted of 50:50 ACN:IPA with 5mm ammonium acetate, 0.05% acetic acid. The initial conditions were 99% A, decreased to 10% A over 11.4 minutes at a flow rate of 0.6 mL/minute, followed by column wash and equilibration at initial conditions. The mass spectrometry parameters for lipid analysis were set as follows: capillary voltage 2/1.5kV (positive/negative ionisation mode), sample cone voltage 25V, source temperature 120°C, desolvation temperature 600°C, desolvation gas flow 1000L.h^{-1}, and cone gas flow 150L.h^{-1}. Data were collected in centroid mode with a scan range of 50-2000*m/z* and a scan time of 0.15s. For mass accuracy, LockSpray mass correction was performed using a 200pg.μL^{-1} leucine enkephalin solution (*m/z* 556.2771 in ESI+) in 50:50 H_2O:ACN solution at a flow rate of 10µL.min^{-1}. Lockmass scans were collected every 60s and averaged over 3 scans.

The dataset was processed using XCMS [4], yielding a table of samples (rows) by features (columns). Retention time trimming was applied, selecting only features between 0.45-12 minutes. Also, only features between 120-1400 m/z were considered. To ensure the quality of the features used in this matching, we only used those that were present also in two other datasets in our laboratory.

1.3. Example S5: Airwave plasma HPOS vs MESA serum HPOS

1.3.1. Airwave plasma HPOS

The Airwave health monitoring study [7] was established to study possible health risks from the use of a personal communication device in police and emergency personnel in the United Kingdom. This large epidemiological dataset (~3000 samples) was acquired by the National Phenome Centre at Imperial College London. Airwave utilised lithium heparin plasma samples stored at -80 °C previous to the analysis. The complete sample preparation and acquisition details can be found in [8]. Samples were thawed at 4°C for 2h. Subsequently, 100 µL of samples were spiked with 10 µL of HILIC internal standards (details can be found in Izzi-Engbeaya et al., 2018). Three parts of acetonitrile were then added to one part of the sample for protein precipitation followed by mixing and centrifugation to separate precipitated protein from supernatant aliquoted for the analysis using LC and MS parameters reported in [9].

The dataset was processed using XCMS [4], yielding a table of samples (rows) by features (columns). Retention time trimming was applied, selecting only features between 0.55-6.2 minutes. To ensure the quality of the features used in this matching, we only used features that were present also in two other datasets in our laboratory.

1.3.2. MESA1 serum HPOS

This large dataset (~2000 samples) was acquired by Metabometrix, a London-based metabolomics services company. MESA [1] human serum samples were stored in -80°C. For sample preparation, they were thawed, and three parts of cold isopropanol were added to each sample, followed by incubation of 2 h at -20°C, centrifugation, and analysis of the supernatant by an Acquity UPLC system coupled to a Xevo G2 ToF mass spectrometer (Waters Corporation). The metabolites were separated by Hydrophilic interaction ultra-performance liquid chromatography. The mobile phases were composed of: A - 100% ACN + 0.1% formic acid; B - H_2O, 20mM Ammonium formate, 0.1% formic acid. The gradient was initiated at 95% A with a flow rate of 0.6 mL/minute. A was decreased to 50% over 6.75 minutes and held at 50% for 1.15 minutes before equilibration prior to injection.

The dataset was processed using XCMS [4], yielding a table of samples (rows) by features (columns). Retention time trimming was applied, selecting only features between 0.4-6.0 minutes. Only features detected in at least 20% of the samples were considered.

1.4. Example S6: MESA serum HPOS vs Airwave urine HPOS

1.4.1. MESA2 serum HPOS

This large dataset (~2000 samples) was acquired by Metabometrix, a London-based metabolomics services company. MESA [1] human serum samples were stored in -80°C. For sample preparation, they were thawed, and three parts of cold isopropanol were added to each sample, followed by incubation of 2 h at -20°C, centrifugation, and analysis of the supernatant by an Acquity UPLC system coupled to a Xevo G2-S ToF mass spectrometer (Waters Corporation). The metabolites were separated by Hydrophilic interaction ultra-performance liquid chromatography. The mobile phases were composed of: A - H_2O, 20mM Ammonium formate, 0.1% formic acid; B - 100% ACN + 0.1% formic acid. The gradient was initiated at 95% B with a flow rate of 0.6 mL/minute. B was decreased to 80% over 4.5 minutes, then decreased to 50% over 0.9 minute and held at 50% for 1.5 minutes before equilibration prior to the subsequent injection. The dataset was processed using XCMS [4], yielding a table of samples (rows) by features (columns). Retention time trimming was applied, selecting only features between 0.5-6.5 minutes. Additionally, features that correlated more than 0.75 with a peak due to undesired polymeric compound were deleted.

1.4.2. Airwave urine HPOS

The Airwave health monitoring study [7] was established to study possible health risks from the use of a personal communication device in police and emergency personnel in the United Kingdom. This large dataset was acquired by the National Phenome Centre at Imperial College London. The details have been published previously [9]. The data acquisition was performed using an ACQUITY UPLC (Waters Corp., Milford, MA, USA) coupled to a Xevo G2-S oaTOF MS (Waters Corp., Manchester, UK) operating in positive ESI mode.

The dataset was processed using Progenesis QI, yielding a table of samples (rows) by features (columns). Retention time trimming was applied, selecting only features between 0.6-6.5 minutes.

2. Procedure notes

2.1. Reference and target datasets

The terms "reference" and "target" dataset refer to the two datasets to be matched. All thresholds and calculations are made and plotted using the "reference dataset". Due to the way the inter-dataset shifts are found, the method is not symmetric, meaning different results may be obtained by swapping reference and target datasets. Nevertheless, in our experience, very similar results are expected regardless of the choice of reference and target datasets.

For each dataset we are provided with a set of features to match, defined by (RT, MZ, FI) values. Note that these are summary level data, not individual level ones. For example, the FI of a feature corresponds to the median FI for that feature across all samples in the dataset. This has the advantage that sample- or individual-specific information is not required to run the algorithm.

2.2. Use of FI for matching

Though usually we discuss the use of RT, MZ and FI for matching, not all have to be used, depending on the application and on dataset similarity. For example, when the datasets are very similar (e.g., different batches of the same experiment) one could use RT, MZ and FI. Conversely, when matching datasets peak-picked using different software or from different populations or tissues, the FI is not expected to be similar in the two sets and the FI weight (in W_{FI}) can be set to zero. It may be necessary to adjust the target FI to the reference, which is explained in section 4.3.

2.3. Metabolomic feature aggregation

For best results, during peak-detection and integration it advantageous to keep the features of all adducts and isotopic forms of a metabolite, instead of combining them into a single feature. This is because, if the features are combined, then a single (MZ, RT, FI) triad will represent the feature and it may not be the same (MZ, RT, FI) selected in both datasets, and thus a match may not be found. Also, for each metabolite, in case of undesirable effects such as detector saturation, abnormal peak integration, missing adducts, there is increased probability that at least one of the adducts/isotopic forms will match the corresponding one on the other dataset, thus increasing the number of matched features available and of good quality for later statistical evaluation.

2.4. Metabolomic feature quality control

For better matching results, before applying the method, the independently peak-picked reference and target datasets can be cleaned of low-quality features, to reduce the number of poor matches. The quality control (QC) procedures may include using laboratory-defined retention time and m/z ranges of interest, evaluating the variability of quality control samples for each variable, evaluating the regression quality of dilution quality control samples, counting the number of samples containing the peak, among others [10]. Although improving the quality of the matches, this procedure also reduces the number of matches, as deleting low-quality features in even only one of the sets reduces the matching across sets.

3. Supplementary figures

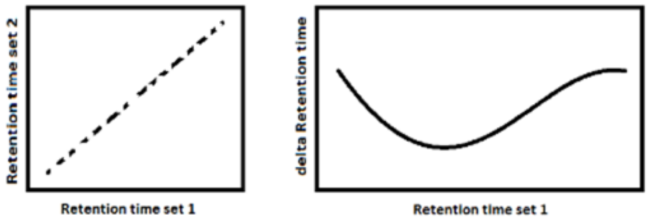

Figure S 1: (left) Example of retention time (RT) correlation between target and reference datasets; (right) example of RT distance (RTdist) between RT of target and reference features as a function of the reference RT. Similar plots can be also made for MZ and log10FI.

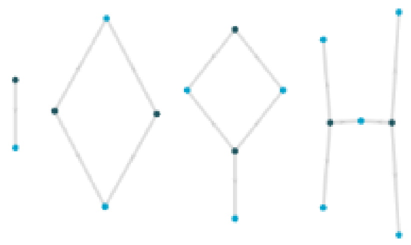

Figure S 2: Possible occurrences of poor matches in four multiple-match clusters, or connected components (CCs), with reference features in dark blue, target features in light blue). From left to right: single match; multiple-match cluster with two features in both reference and target; multiple-match cluster with two features in reference and three in target; multiple-match cluster with two reference and five target features. The single match cluster will yield one match, while all others may each yield (in this example) a maximum of two matches per cluster.

4. Methods

In this section we describe the matching method in more detail than space would allow in the main paper.

4.1. Calculation of all distances between features in the two datasets (part of step 1)

Considering a reference dataset with *R* features, and a target dataset with *T* features, the inter-dataset distance in each dimension can be calculated for each pair of reference-target features as:

$$RTdist_{tr} = RT_{target\ t} - RT_{ref\ r} \quad (1a)$$

$$MZdist_{tr} = MZ_{target\ t} - MZ_{ref\ r} \quad (1b)$$

$$log_{10}FIdist_{tr} = log_{10}FI_{target\ t} - log_{10}FI_{ref\ r} \quad (1c)$$

4.2. Definition of initial matching thresholds (part of step 1)

Two lines in the per dimension plots (e.g., RTdist vs RT) define the upper and lower limits of the matching areas (see e.g., Figure SE1 3), and they describe absolute threshold values if the slope is defined as zero or relative values otherwise. The equations below are used twice in each dimension (for lower and upper thresholds, using two different values of intercept and slope). The intercept and slope parameters are defined by the user.

$$RTdist_{Threshold(tr)} = RT_{intercept} + RT_{slope} \cdot RT_{ref(r)} \quad (2a)$$

$$MZdist_{Threshold(tr)} = MZ_{intercept} + MZ_{slope} \cdot MZ_{ref(r)} \quad (2b)$$

$$log_{10}FIdist_{Threshold(tr)} = log_{10}FI_{intercept} + log_{10}FI_{slope} \cdot log_{10}FI_{ref(r)} \quad (2c)$$

The cases when the three values in the triad ($RTdist_{tr}$, $MZdist_{tr}$, $log_{10}FIdist_{tr}$) are within their respective thresholds become candidate matches. In those cases, the feature-related subscript "tr" is substituted by the match-related "m" and a total of M candidate matches (including multiple ones) is found for the two datasets (Figure 1, bottom panel, step 1). The cases where that triad is not within the thresholds do not originate candidate matches, and those are not considered any more in the procedure.

4.3. Adjust FI of target to FI of reference (part of step 1)

Considering that after matching features between two datasets a number of matches have been obtained, at this point it is possible to compare their $log_{10}FI$. Again, note that we only consider the median of the log10FI across each dataset, and ignore values for individual samples. If no good correlation between the median $log_{10}FI$ in the two sets is observed, then $log_{10}FI$ should not be used for matching. Otherwise, for datasets with similar characteristics it is reasonable to expect that a large peak in one is also large in the other (on average) and FI can be considered for matching. Correlation does not require identical values in both sets, thus the $log_{10}FI$ values of target can be adjusted to the reference before any calculations. Two methods are proposed:

4.3.1. Median method

To simply adjust robustly for a systematic difference, assume the offset as the median difference between $log_{10}FI$ of all matches:

$$FI_{offset} = \text{median}\,(log_{10}FI_{target} - log_{10}FI_{ref}) \tag{3a}$$

and subtract it from each target feature in a match *m*:

$$log_{10}FI_{target(m)} = log_{10}FI_{target(m)} - FI_{offset} \tag{3b}$$

as shown in Figure SE1 4.

4.3.2. Regression method

In this case the adjustment is performed using robust linear regression of $log_{10}FI_{ref}$ vs $log_{10}FI_{target}$ of the two sets:

$$log_{10}FI_{ref} = slope\ .\ Log_{10}FI_{target} + intercept \tag{3c}$$

and then obtain the adjusted $log_{10}FI_{target}$ as the model's predicted values of $log_{10}FI_{target}$.

4.4. Define neighbours (part of step 2a)

The neighbours of a feature *m* are the *k* features in the same dataset that are closest to that feature, either in each dimension (RT, MZ, FI) separately, or as a consensus including multiple dimensions. The number of neighbours can be directly defined (e.g., 21) or as a percentage of the number of features in the reference dataset without considering features in clusters of multiple matches (e.g., 0.01 for 1% of the number of reference features not in clusters). There are two methods to define neighbours, "cross" and "circle", these names arising from the pattern the neighbours form in the MZ vs RT plot (Figure S 3 and Figure S 4, left plots).

In order to reduce the influence of features that are part of matches but will not be selected in the end (either because they were in multiple matches or in poor matches), only features in single matches are used to sample the neighbours. This is applied in both methods.

4.4.1. "Cross" method

The "cross" method defines *k* neighbours independently in each of the dimensions (RT/MZ/log10FI) and uses those to calculate expected inter-dataset shift (trends) and residuals for each dimension separately. A robust LOESS using a span of 10% (of the number of matches) is then used to smooth the expected inter-dataset shift points (trends) for the complete dataset. This results in the RTdist being the same for features at the same RT_{ref} (and similarly for MZ).

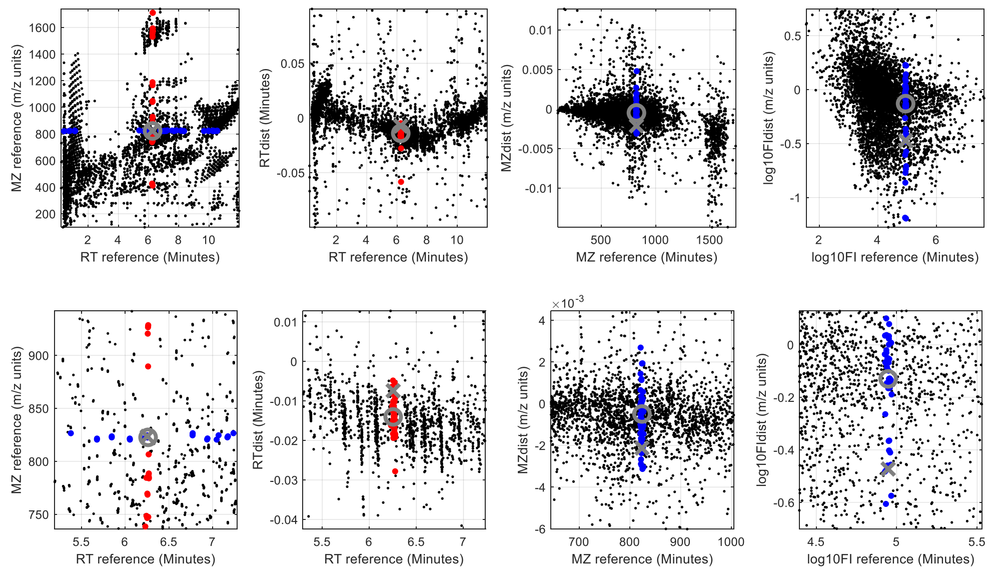

Figure S 3: Definition of neighbours and expected inter-dataset shift for a single match using the "cross" method. A set of independent k neighbours is defined in each dimension, meaning they are the closest features to the feature in question in that dimension. Bottom row shows a zoom of the top row plots. The grey "x" represents the inter-dataset distance for the match in question and the "o" is the corresponding expected inter-dataset shift, red and blue dots are the chosen neighbours in each of the dimensions. The first plot on the left is the MZ vs RT of reference and the others are distance plots as previously presented.

4.4.2. "Circle method"

The "circle" method defines *k* neighbours in (normalised) RT and MZ simultaneously (thus finding the same features as neighbours in both dimensions) by evaluating the Euclidean distance using those dimensions only, not FI. This means that the RTdist (and/or MZdist) can be different for features at similar RT_{ref}. Because the expected log10FI is not necessarily similar for neighbour features in the MZ vs RT space, log10FI is not used in this method and the expected inter-dataset shift for log10FI is defined as zero. Thus, in practice the residuals for log10FI are directly obtained by reference log10FI subtraction from target log10FI.

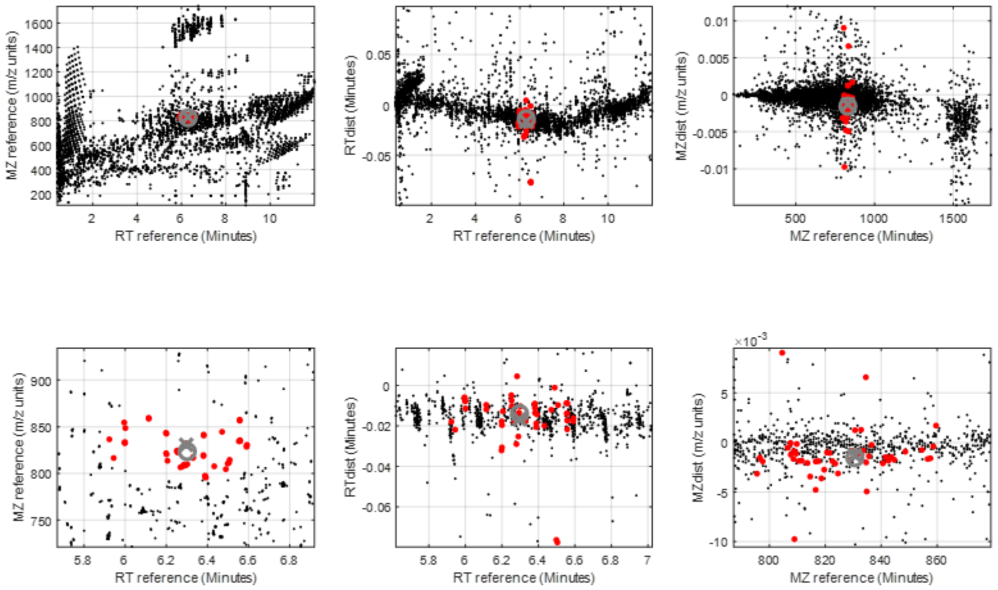

Figure S 4: Definition of neighbours and expected distances for a random feature using the "circle" method. A set of k neighbours is defined simultaneously for RT and MZ using Euclidean distance. The bottom row of plots shows a zoom of the top row. The grey "x" represents the inter-dataset distance for the feature in question and the "o" is its expected inter-dataset shift. Notice there is no FI dimension in this method.

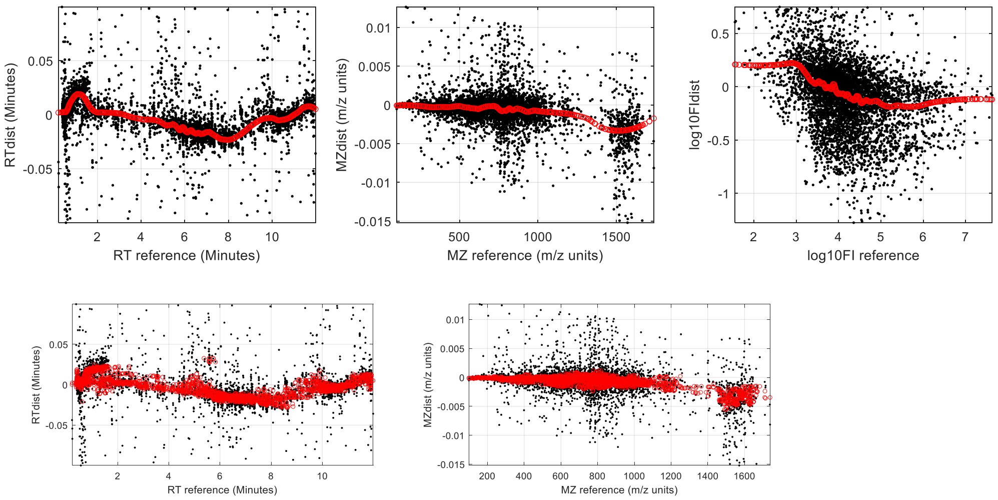

Figure S 5: Match distances (black dots) in each of the dimensions and expected distances (red circles) calculated by the two methods ("cross" and "circle") using 1% of the total number of reference features as neighbours. Top row: Independently calculated expected distances in each dimension using the "cross" method. Bottom row: Consensus expected distances for RT and MZ using the "circle" method. The "cross" method seems to define more tight expected values than the "circle" method, as the latter allows for different e.g., RTdist for the same RT of a reference. Notice that while the "cross" method red points are actually a smooth line, the ones in the "circle" are individual unsmoothed points.

4.4.3. Difference between the two methods

Importantly, the "circle" method allows for clusters of features at e.g., same RT but different MZ to present different inter-dataset RT shift trends, and this does not happen in the "cross" method, where matches at the same reference RT have a single inter-dataset RT shift trend (the same would apply for MZ). In other words, the points obtained by the "cross" method can be smoothed by adjusting a line to it, while the points in the "circle" method are left unsmoothed (as there could be multiple "lines" for each e.g., RT of the reference). As an example of this effect, consider the reference features (left plot) in Figure S 6 and compare the groups of features at RT = 8 minutes and MZ = 700 m/z to the features with the same RT at around MZ = 1600. If those features had different physico-chemical properties, then in the target dataset (right plot) the two groups should not elute at the same retention time, and the use of the "circle" method may be advantageous, modelling both individually. The left plots on Figure S 5 actually show this effect in practice, as while in the "cross" plot there is only one line (in red), at $RT_{ref} < 2$ minutes the "circle" method presents two almost parallel sets of points, meaning it is finding the distances differently for two groups of matches at the same RT_{ref}. Another example is presented in Figure S 6.

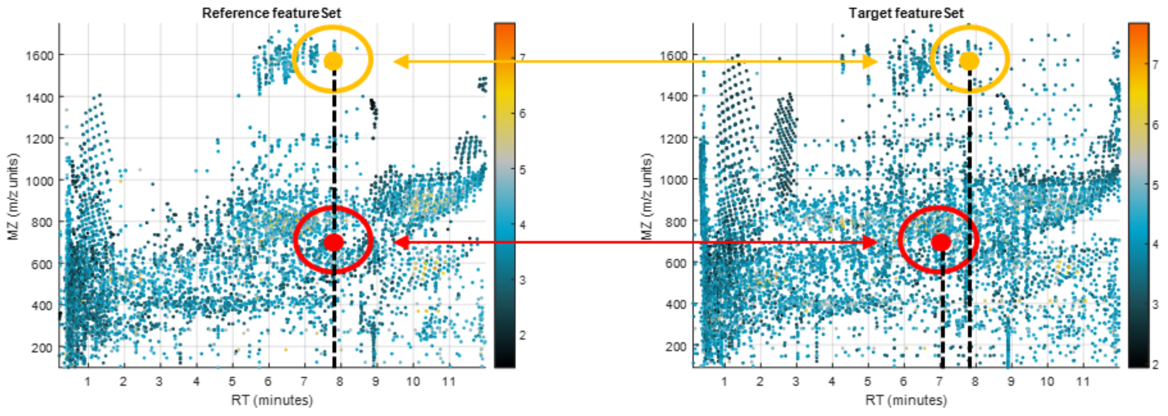

Figure S 6: The expected RT inter-dataset shift at the same RT=8 minutes value is not necessarily the same for clusters of features at different values in MZ, and the "circle" method can model both separately. These two matches could both be at their own expected inter-dataset shift regardless of being at the same retention time. That would not happen if using the

“cross” method, as there is a single expected inter-dataset shift for each RT value. (NOTE: The same applies to inverse roles of RT and MZ).

4.5. Step 2a: Define inter-dataset shift using feature neighbours

To find the inter-dataset shift at a specific e.g. RT value, start by finding the *k* nearest neighbours of its reference feature in the (MZ, RT, log_{10}FI) space of the reference dataset according to one of the methods previously described. Then calculate $RTdist_{neigh(m)}$, the RTdist for each of its *k* neighbours. Finally, define the expected inter-dataset shift $RTdist_{expected(m)}$ for that match as the median (or other averaged measure) value of its neighbours' $RTdist_{neigh(m)}$ as shown in Figure S 5 and calculated below:

$$RTdist_{expected(m)} = median(RT_{target\_neigh(m)} - RT_{ref\_neigh(m)}) \tag{6a}$$

The same procedure is also applied to calculate the $MZdist_{expected(m)}$ and $log_{10}FI_{(m)}$:

$$MZdist_{expected(m)} = median(MZ_{target\_neigh(m)} - MZ_{ref\_neigh(m)}) \tag{6b}$$

In the “circle” neighbours' method FI is not used, so one defines $log10FIdist_{expected(m)} = 0$. For the “cross” method,

$$log10FIdist_{expected(m)} = median(log_{10}FI_{target(m)} - log_{10}FI_{ref(m)}) \tag{6c}$$

4.6. Step 2b: Calculate and normalise residuals

4.6.1. Calculate residuals

RT and MZ residuals ($\Delta dist_{(m)}$) are the difference between the e.g. $RTdist_{(m)}$ of each candidate match and its expected inter-dataset shift value $RTdist_{expected\ (m)}$, as in row 3 of Figure 3 (in main paper) according to:

$$\Delta RTdist_{(m)} = RTdist_{(m)} - RTdist_{expected(m)} \tag{7a}$$

$$\Delta MZdist_{(m)} = MZdist_{(m)} - MZdist_{expected(m)} \tag{7b}$$

For the FI dimension the calculation depends on the neighbours' method chosen. In the neighbours' “cross” method the FI residuals are calculated similarly to the other dimensions:

$$\Delta log_{10}FIdist_{(m)} = log_{10}FIdist_{(m)} - log_{10}FIdist_{expected(m)} \tag{7c}$$

In the neighbours' “circle” method we defined $log10FIdist_{expected(m)} = 0$, so FI residuals end up as simply the difference between target and reference, or $log_{10}FIdist_{(m)}$.

4.6.2. Normalise residuals

The objective in this section is to normalise the values of the residuals in each dimension (RT, MZ, log_{10}FI) to control their dispersion, by attributing them the same value (of 1) at a specific threshold point. The residuals of the three dimensions are the distances to the inter-dataset shift trends observed in each dimension, and by being defined in different units (RT minutes, m/z units, log_{10}FI units) need to be normalised before being combined into penalisation scores. A robust z-like score is obtained (in each dimension separately) by dividing them by a threshold point defined as the median

of the values plus a factor F (default = 3) times the median absolute deviation (MAD). The general expression is

$$threshold\ point_x = median(x) + F * MAD(x) \quad (8a)$$

and thus, applied to each dimension becomes:

$$threshold\ point_{\Delta RTdist} = median(\Delta RTdist) + F * MAD(\Delta RTdist) \quad (8b)$$

$$threshold\ point_{\Delta MZdist} = median(\Delta MZdist) + F * MAD(\Delta MZdist) \quad (8c)$$

$$threshold\ point_{\Delta log_{10} FIdist} = median(\Delta log_{10} FIdist) + F * MAD(\Delta log_{10} FIdist) \quad (8d)$$

The standardisation becomes thus:

$$norm\Delta RTdist_{(m)} = \frac{\Delta RTdist_{(m)}}{threshold\ point_{\Delta RTdist}} \quad (9a)$$

$$norm\Delta MZdist_{(m)} = \frac{\Delta MZdist_{(m)}}{threshhold\ point_{\Delta MZdist}} \quad (9b)$$

$$norm\Delta log_{10} FIdist_{(m)} = \frac{\Delta log_{10} FIdist_{(m)}}{threshold\ point_{\Delta log_{10} FIdist}} \quad (9c)$$

The threshold point defines a percentile of the residuals. The threshold point could also be decided by the analyst directly, by just choosing the desired value of the residuals to be the threshold point, from visually inspecting the residuals on e.g., Figure SE1 9. After this adjustment the residual value for all dimensions is 1 at the threshold points decided (see e.g., Figure SE1 10), and the residuals of all dimensions can be combined into a single value.

4.7. Step 2c: Define weights for each dimension's residuals

The normalisation of the residuals to the value of 1 at the value of the residuals' dMAD (or defined percentile) allows one to understand the impact of the residuals of each dimension and helps define their weights $W_{RT,MZ,FI}$. In the simplest case, and by default, $W_{RT,MZ,FI}$ can be the same in all dimensions ([1, 1, 1]). Alternatively, the value of $W_{RT,MZ,,FI}$ can be manually defined by inspection of residual plots such as in row 4 of Figure 3 (in main paper). For cases where e.g., FI is not relevant, define $W_{FI} = 0$. Notice that the FI dimension should only be considered in case the feature intensities are highly correlated and thus comparable in both datasets.

4.8. Step 2d: Calculate penalisation scores

The penalisation score is a weighted root sum of squares of the normalised residuals:

$$Score_m = \sqrt{(W_{RT} \cdot norm\Delta RTdist_{(m)})^2 + (W_{MZ} \cdot norm\Delta MZdist_{(m)})^2 + (W_{FI} \cdot norm\Delta log_{10} FIdist_{(m)})^2} \quad (10)$$

4.9. Step 2e: Select best matches in multiple-match clusters

Consider Figure SE1 13 showing all the matches. Most of those are single matches, though there are some to the left that are in multiple-match clusters and decisions need to be taken regarding which matches to select. A simple greedy algorithm is used. For each cluster with multiple matches select first the match with lowest penalisation score (the "best" match). After deleting the matches that are not possible anymore once the features in the best match are removed, the match with the lowest

score in the remaining network becomes the new "best" match. This process is iterated until no features remain. In the end the cluster yields at least one match, though it could yield more.

4.10. Step 3: Detect poor matches (tighten thresholds)

After choosing the best matches in the multiple match clusters there are only unique matches. Poor matches are defined as unique matches far away from the inter-dataset shift trends, which obtain a high penalisation score. Those matches are more probable to have happened by chance than the ones closer to the shift trends, and optionally can be deleted.

4.10.1. "Trend mad" method

In this method, the unique matches from step 2 are found and the procedure is restarted from the beginning using only those ones: 1. The neighbours and residuals are recalculated for the three dimensions (RT/MZ/log10FI). 2. It then finds a threshold value (see "Normalise residuals") in each dimension. 3. It defines the poor matches as the ones outside limits in at least one dimension. Note that this method uses the "cross" method for the calculation of neighbours and thus uses the residuals of the three dimensions.

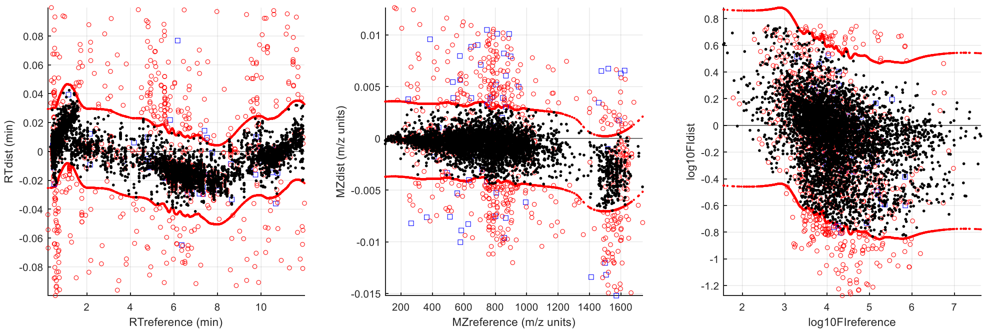

Figure S 7: Example of "Trend mad" method result showing distance plots in each of the dimensions with red lines representing the threshold values, as well as step 2 rejected matches from clusters (blue), good matches (black) and poor matches (red). Poor matches are the ones that are outside threshold points in at least one dimension.

4.10.2. "Scores" method

In this method, the calculated penalisation scores are used to find the poor matches, by finding the ones larger than a threshold value (see "Normalise residuals"). The procedure has the following steps: 1. Collect only the penalisation scores of the final single matches selected (multiple matches have already been deleted). 2. Calculate the threshold value for the penalisation scores; 3. Find the matches that are outside the penalisation score threshold points, those are the poor matches.

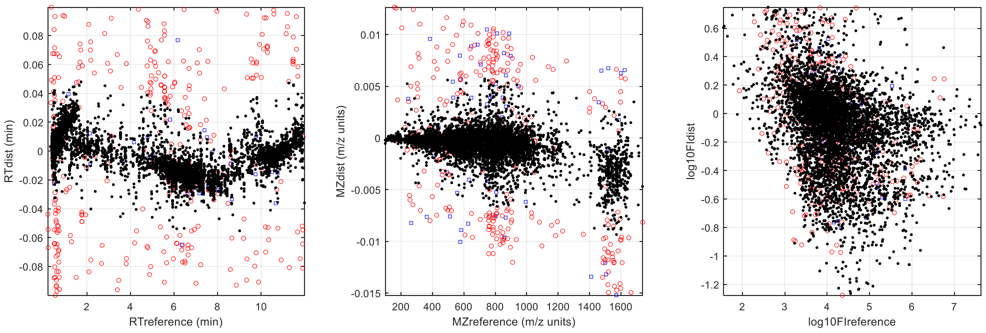

Figure S 8: Example of "Scores" method result showing distance plots in each of the dimensions with good matches (black), poor matches (red) and false matches that were part of clusters and were not selected (blue). There are no limit lines in the plots as what is evaluated is the penalisation score, which is an aggregate measure of the residuals.

5. Example S1: MESA serum LPOS vs Rotterdam serum LPOS

Here we give a detailed description of the matching of Dataset 1 in the article.

5.1. Matching procedure

The complete example of the matching of features of serum LPOS MESA (reference) vs Rotterdam (target) presented in the main body of the article is shown below.

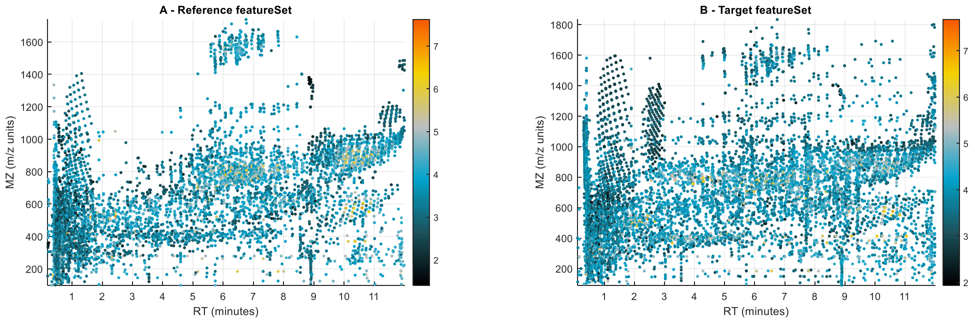

Figure SE1 1: MZ vs RT plots of serum lipid positive mode datasets after RT trimming, coloured by log10FI.

5.1.1. Step 1: Match all features within thresholds

Initially large values of RT, MZ, FI thresholds may be used to guarantee that all possible matches are found and for a trend in deviation to be observed. An initial matching with large thresholds was calculated, to detect all possible matches and to visualise trends between the two datasets, where some of the inter-dataset trends were clearly observed (Figure SE1 2). In our lipidomics datasets a maximum of RT_{thresh} = 30 seconds, MZ_{thresh} = 100 ppm, $log_{10}FI_{thresh}$= 3 is in general enough. The following large thresholds were used:

```
opt.FIadjustMethod = 'median';

opt.multThresh.RT_intercept = [-1, 1];
opt.multThresh.RT_slope = [0 0];
opt.multThresh.MZ_intercept = [-0.025, 0.025];
opt.multThresh.MZ_slope = [0, 0];
opt.multThresh.log10FI_intercept = [-1000, 1000];
opt.multThresh.log10FI_slope = [0 0];
```

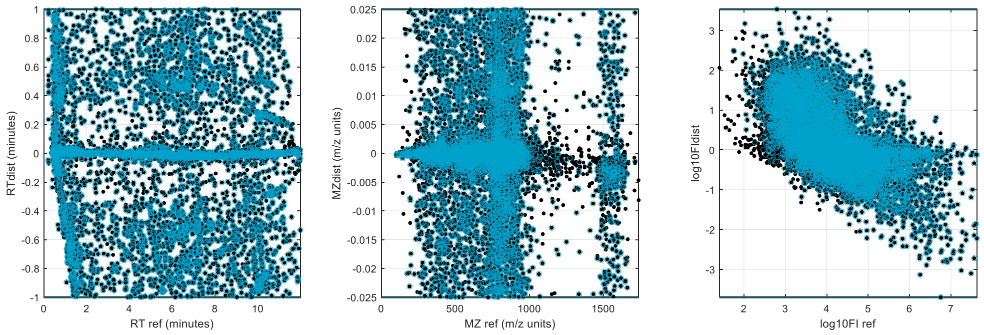

Figure SE1 2: Initial matching with large thresholds, to detect major inter-dataset shift trends. The RT and MZ trends are clearly seen in the respective plots, while the FI dimension is less clear. Matches containing features involved in multiple match clusters s are shown in blue, unique matches in black.

The FI was adjusted using the “median” method, which subtracts to the target matched features the difference between the medians of the two datasets.

By visually inspecting the plots in Figure SE1 2, it was observed that there is a cloud of points in $\log_{10}$FI that does not correlate well between the two datasets and thus the $\log_{10}$FI threshold was defined in a way as to delete those matches. After several tests with different settings, the Log_{10}FI adjustment method and the settings for RT/MZ/$\log_{10}$FI thresholds were decided as:

```
opt.FIadjustMethod = 'median';

opt.multThresh.RT_intercept = [-0.1, 0.1];
opt.multThresh.RT_slope = [0, 0];
opt.multThresh.MZ_intercept = [-0.0075, 0.015];
opt.multThresh.MZ_slope = [-0.01/2000, -0.01/2000];
opt.multThresh.log10FI_intercept = [-1.4, 0.75];
opt.multThresh.log10FI_slope = [0, 0];
```

After RTdist, MZdist, and log10FIdist thresholds are set, every pair *p* of reference-target features with these distances lower than all respective thresholds is a match *m* (Figure SE1 2). New peak tables with columns RT, MZ, and FI (as in Figure 1, step 1) for each inter-dataset match *m* are defined. Notice that at this point we change from referring to RTdist in terms of the features (as in RTdist_{ij}) and refer to it in terms of its match index m (as in RTdist_m) These two tables contain information on all matches, and the matched features of reference and target are in the same row. Also, features may appear multiple times in the table, as one feature in the reference dataset may find multiple matches in the target dataset, and vice-versa. This procedure can be repeated with different threshold values, for optimization purposes.

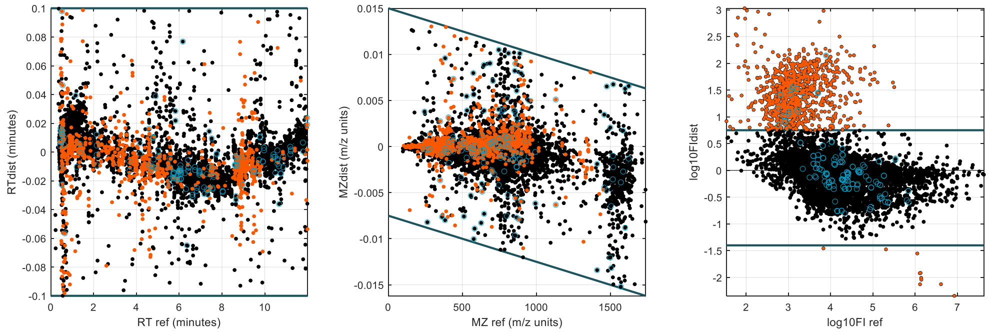

Figure SE1 3: Matches within thresholds in the RT, MZ, log10(FI) domains between LPOS datasets. (left) RT distance of target to reference; (centre) MZ distance of target to reference; (right) log10(FI) of target vs reference. Absolute thresholds are represented as horizontal lines, relative threshold as diagonal lines. Matches are represented as black dots; if part of a cluster of multiple matches their outline is emphasised in blue; if outside the log10FI thresholds they are coloured in orange.

In order to use the FI for matching the log10FI of both datasets is harmonised, using in this case the “median” method (see SI section “Adjust FI of target to FI of reference”).

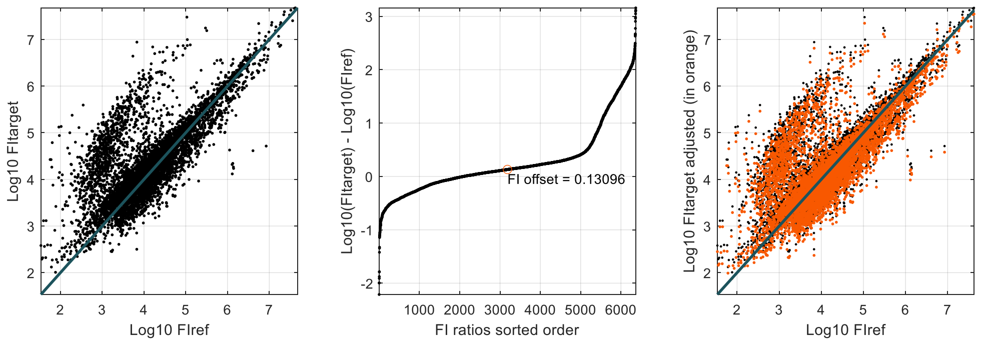

Figure SE1 4: Results of harmonisation of FI in the two LPOS sets, using the 'median' method. (left) Log10 FI of target vs reference; (centre) Sorted values and median of difference between Log10 FI of target and reference; (right) Initial (black dots) and corrected values (orange dots) of Log10 FI of target to reference.

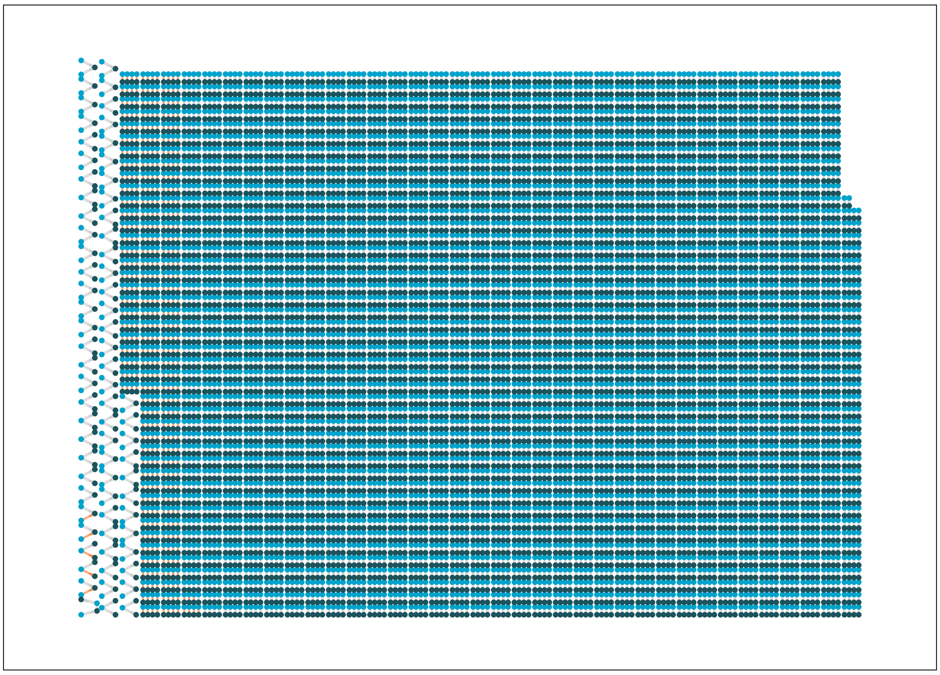

Figure SE1 5: All possible matches (edges, as lines) within thresholds between the features (nodes, as dots) of the two LPOS sets. Reference features in dark blue, target features in light blue. Orange edges represent the matches outside of log10FI thresholds.

5.1.2. Step2: Find unique correspondence

The two datasets may be shifted in each of the dimensions, and that inter-dataset shift should be modelled. For each feature, its inter-dataset shift in a dimension is given by the median of the shifts of its neighbours. Thus, the first step in this process is to find the k-nearest neighbours of each feature, and this search is only performed within the set of features that are not involved in multiple match clusters (Figure SE1 6). The neighbours for the calculation of residuals were found using the following definitions:

opt.neighbours.nrNeighbors = 0.01;

opt.calculateResiduals.neighMethod = 'cross';

This means that the “cross” method will be used, with the number of neighbours for each feature set to 1% of the total number of features that are not part of multiple match clusters.

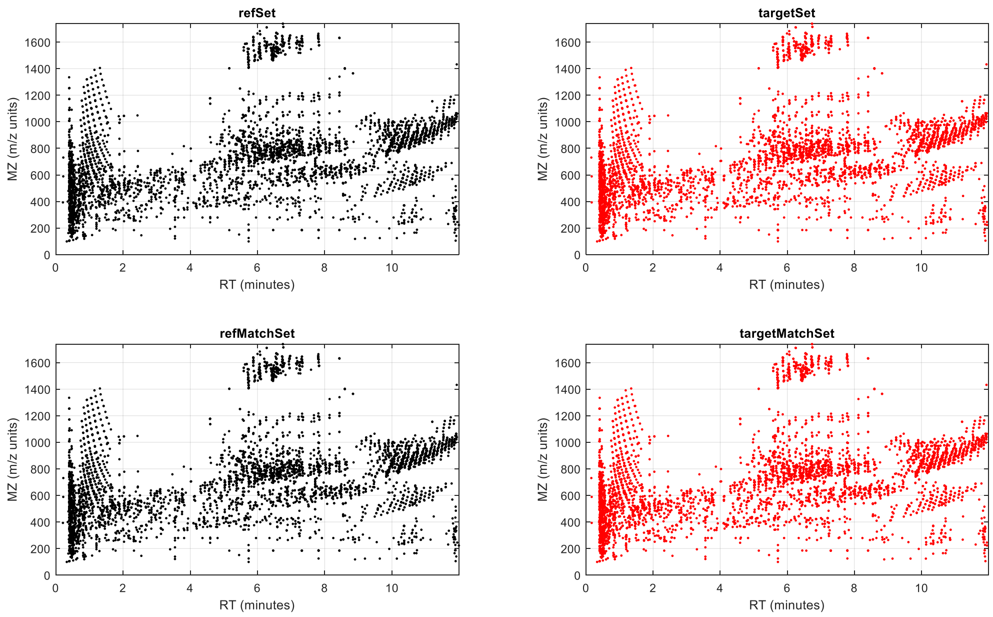

Figure SE1 6: (top) Features captured in matches within thresholds in the LPOS datasets, including the ones in multiple-match cases; (bottom) features with only single match possibilities used to calculate neighbours in LPOS datasets. Notice the similarity between corresponding reference and target sets (left and right figures), as well as the similarity between the sets and match sets (top and bottom figures) as in this case most matching features only single match.

Using the “cross” method, the k features closest to a feature using Euclidean distance on normalized MZ and RT values are the chosen ones for neighbours of that feature.

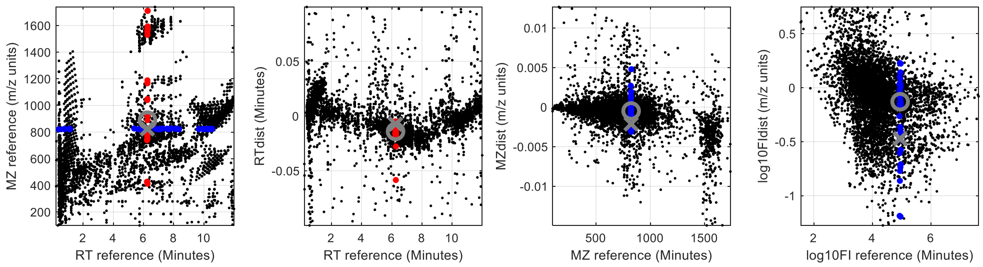

Figure SE1 7: Example of calculation of neighbours for a random feature (in this case feature with index 2713) using the “cross” method. This method selects neighbours of a feature in each dimension independently, thus the neighbours in each dimension are not necessarily the same. The red and blue dots are the closest neighbours of the feature highlighted with a grey cross; the grey circle is the median of the neighbours indicating the inter-dataset shift trend for that dimension for that

The median inter-dataset difference of the neighbours of a feature represents the inter-dataset shift for the feature (after robust loess smoothing using 1% of the points), as seen in Figure SE1 8.

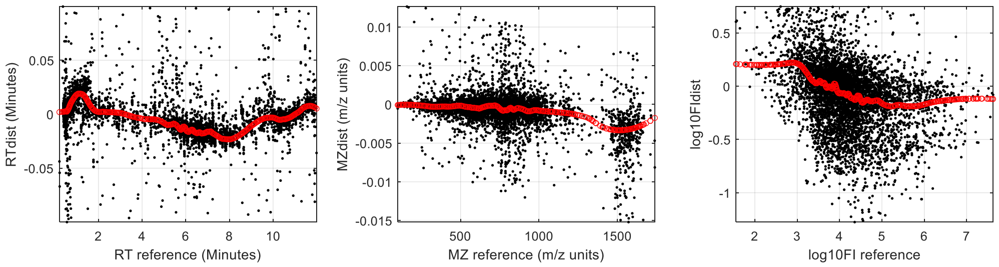

Figure SE1 8: Expected smoothed values (in red) of the inter-dataset shift using the "cross" method for each feature (black dots) used to calculate the residuals in the RT, MZ, $log_{10}FI$ domains.

By subtracting the value of the expected inter-shift distances of a feature from each distance between that feature and its corresponding match in the other dataset, one obtains the residuals (see Figure SE1 9). These indicate the distance of the match to the inter-dataset shift distance.

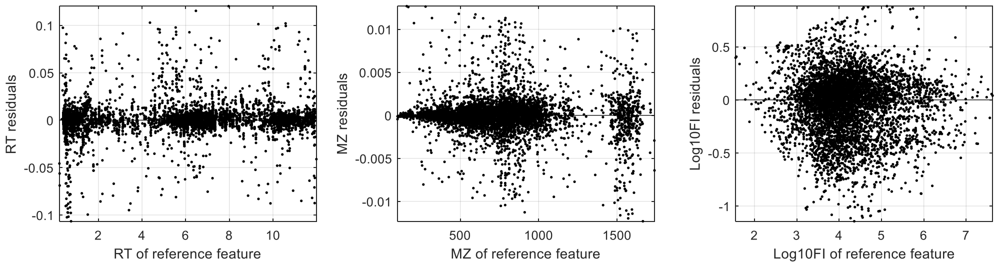

Figure SE1 9: Residuals in the three domains, to be used in the calculation of the penalisation scores for each match.

The threshold point calculated with median + 3*MAD was used to find the value of the residuals to be used for normalisation (to divide by) in each of the dimensions and those values were at the percentiles 91.8/92.4/95.0 for (RT, MZ, $log_{10}FI$), respectively. After dividing by the value at those percentiles, the residuals were normalised (Figure SE1 10).

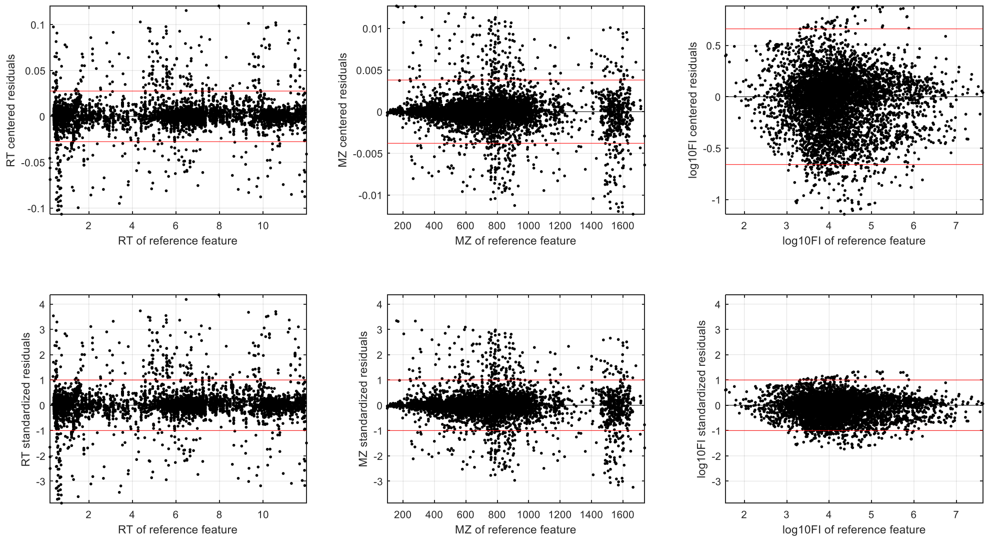

Figure SE1 10: (top) Median-centred residuals for RT/MZ/log10FI and red lines indicating residuals' percentiles 95.8, 96.0, 99,2 found using the median plus 3 MAD of the residuals in the RT, MZ, log10FI dimensions, respectively. These values will be used as pivot and will become =1 in the normalised residuals; (bottom) normalised residuals after dividing the median-centred residuals by the percentile values. Notice that as the residuals are in normalised units, the bottom plots scale is the same, for magnitude comparison.

After the residuals are normalised, it is easy to combine them into a single value, by weighing them with a value W, which can be different in each dimension. In this case it was decided to give the same weight (1) to the RT and MZ dimensions, while penalising the log10FI with a weight of 0.2 (W = [1, 1, 0.2]), with the resulting weighted residuals represented on Figure SE1 11.

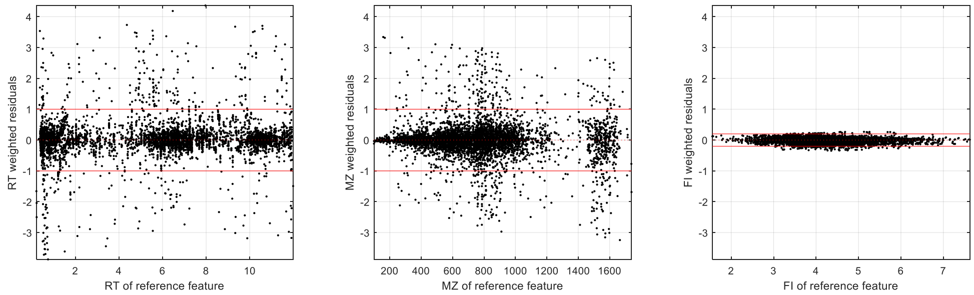

Figure SE1 11: Weighted residuals, after using a unit weight on RT and MZ, but penalising log_{10}FI, as the weights were defined as W=[1, 1, 0.2]. These are used to calculate the penalisation scores (squared root of the sum of squares of the weighted residuals) in the next step. By looking at the plots one can understand that in this case the contributions of RT and MZ are the most relevant dimension in the calculation of the penalisation score, while the contribution of log_{10}FI is much more reduced.

The penalisation scores are then created as the squared sum of squares of the normalised weighted residuals and can be visualised by colouring the previous distance plots (Figure SE1 12).

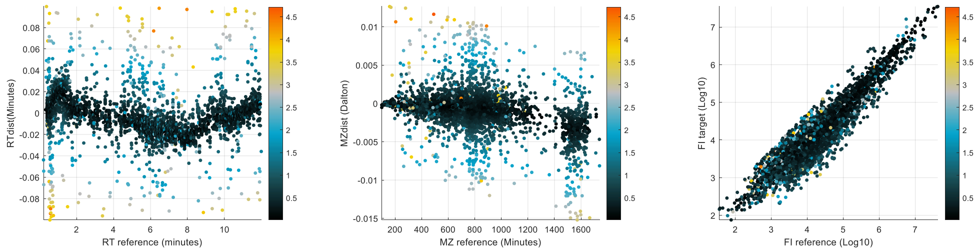

Figure SE1 12: Matches in the RT, MZ, log10(FI) domains between LPOS datasets. (left) RT distance of target to reference; (centre) MZ distance of target to reference; (right) log10(FI) of target vs reference. All plots are coloured by match penalisation scores created from the normalised residuals.

The network of all matches (including clusters of multiple matches) is shown in Figure SE1 13. It is remarkable that most of the clusters only involve one feature from each set (unique matches). This means that all those are already in their final form, and except for outlier matches that stand too far from the inter-dataset shift trends, there is not much room for wrong matches to happen.

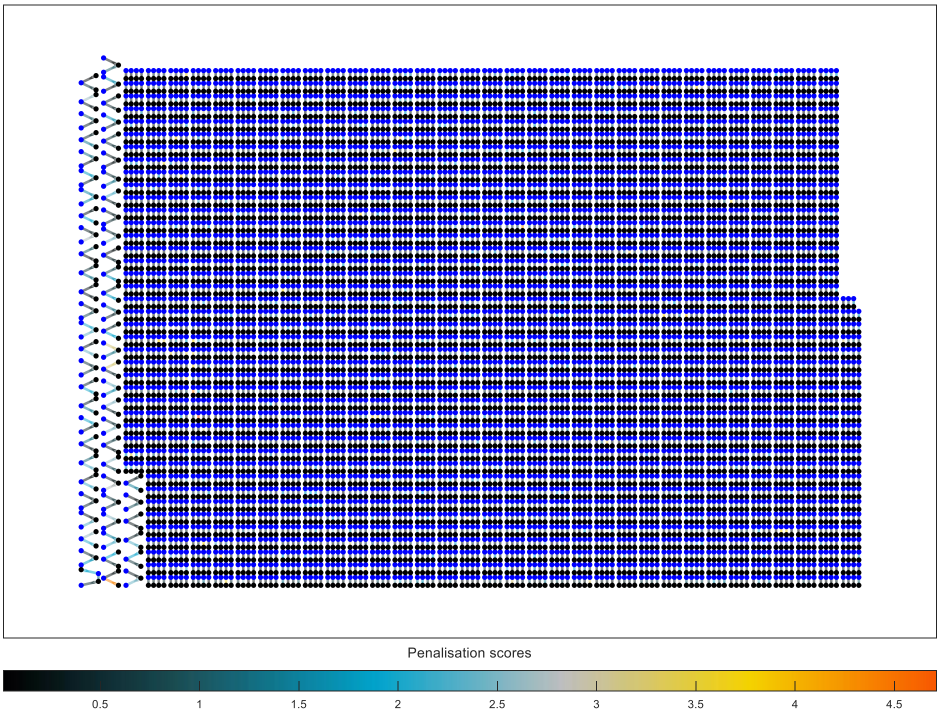

Figure SE1 13: Network with all matches (edges) coloured by penalty scores. Metabolomic features (nodes) of reference in black, target features in blue.

5.1.3.Step 3: Detect poor matches (tighten thresholds)

The initial threshold definition can be tightened so it is possible to find matches that - although unique – are at large distances from the inter-dataset shift trends and produced high penalisation scores. These are the so-called “poor” matches. The method “scores” was used to find the scores that were at a distance larger than the median of the scores plus 3 MAD.

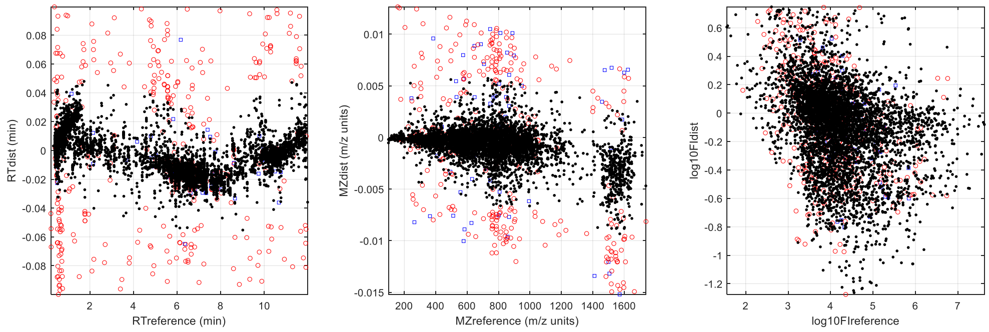

Figure SE1 14: Tightening of thresholds used to define poor matches (in red) using the method “scores” to find matches with penalisation scores higher than the median plus 3 MAD. Multiple matches that were previously deleted in blue; poor matches in red; good matches in black.

The numbers of features and matches during each stage of the process are presented in Figure SE1 15 and in Table SE1 1.

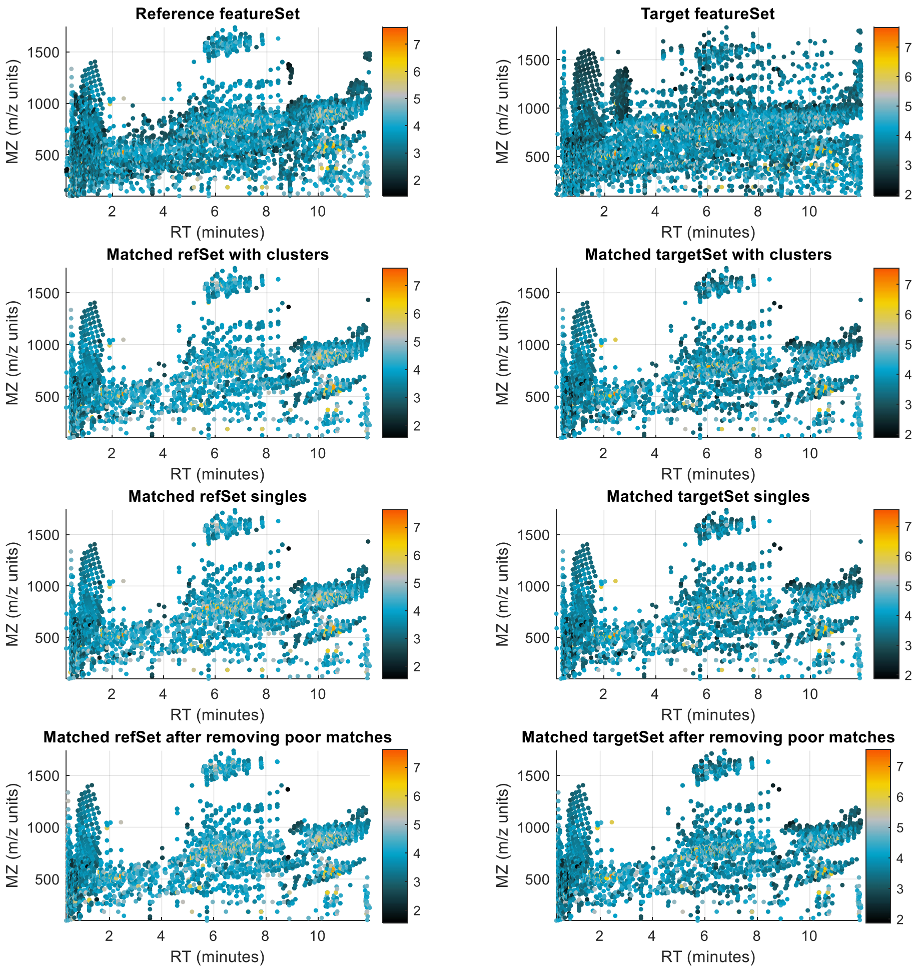

.

Figure SE1 15: MZ vs RT plots of LPOS datasets at each stage of the process (top to bottom) coloured by penalisation scores.

Table SE1 1: Summary of number of matches, features and clusters of multiple matches in the network. There are 5426 total matches, most of them unique (5303 clusters of matches with only 2 nodes, when both features only match to each other). The network of these matches produces 5364 clusters with 2-4 features each, which after recursive division end up yielding a total of 5365 unique matches. Excluding poor matches (by tightening thresholds) results in 4953 matches.

**Total matches**	5426
Reference features	5396
Target features	5394
**Features in only one match**	
In Reference	5366
In Target	*5362*
**Features with multiple matches**	
In Reference	*30*
In Target	*32*
**Clusters of matches**	5364
2 nodes	5303

3 nodes	60
4 nodes	1
**Unique matches including poor**	5365
**Unique matches without poor**	4953

5.2. Validation

5.2.1. Comparison of FI

The composition of plasma is highly regulated thus the median concentration of a metabolite should be of a similar order of magnitude in both sets. Although from different populations, the sample type, extraction, injection and peak-picking methods were similar, and we observe that peak size in both sets shows good agreement on a $\log_{10}$FI scale for most features (plot on the right, Figure SE1 12 and Figure SE1 17).

5.2.2. Comparison of metabolite annotations

The two datasets (MESA and Rotterdam) were thoroughly manually annotated (see Experimental section/Data/Metabolite annotation in the main text), with 604 features in both datasets having the same annotation. Evaluating the number of matches that correctly match two features with the same annotation is the best way to validate the data, though depending on the number of annotations. The results of this strategy are presented in Table SE1 2, Figure SE1 16 and Figure SE1 17.

Table SE1 2: Number of annotated matches in the data at each stage. There are 604 annotations in the initial data of both Reference (10427 features) and Target datasets (14097 features). There are 9 annotated features in each set that could match, but their matches are outside of the defined initial thresholds. After setting thresholds for multiple matching only around 44% of the features in the datasets match to each other (5426 matches). After unique matching (5365 matches) all annotated matches (595) are found to have the correct ID in both datasets. Regrettably, after deleting poor matches (ending in 4953 matches) 10 correctly annotated matches are deleted, ending with a total of 585 (96.8%) correct and 19 (3.1%) incorrect/not found matches.

Stage and results	Number annotations	Number matches
Initial data	**604**	(10427/14097 unmatched features)
Matches outside thresholds		**9**
After all matches within thresholds (step1)	**595**	5426
After unique matches (step 2)	**595**	5365
Correct ID matches	**595**	**-**
Wrong ID matches	**0**	**-**
After removing poor matches (step 3)		4953
Final number of correct ID matches	**585**	
Final number of wrong ID/outside threshold matches	**19**	
Poor matches	**10**	412

With correct ID	**10**	-
With wrong ID	**0**	-

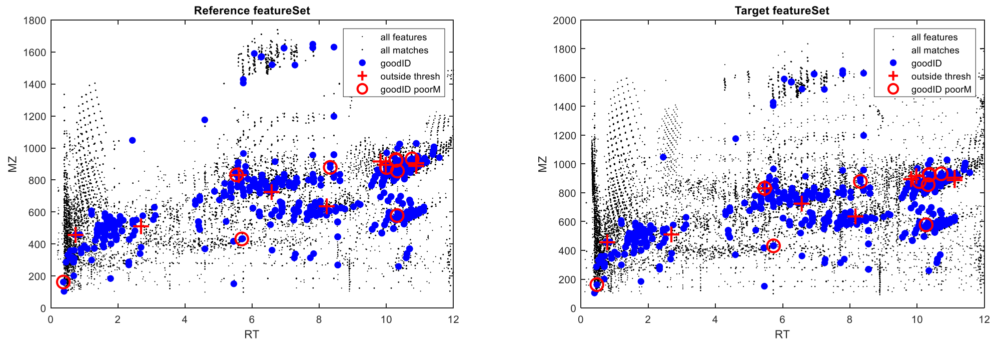

Figure SE1 16: MZ vs RT of reference (left) and target (right) LPOS datasets, with a summary of matching results for each of the metabolomic features. For each plot, small black dots represent all features in the dataset, while larger black dots represent features that were matched. Red "+" show features that were initially found outside of the defined thresholds, blue dots are the annotated features matching a feature with the correct (same) annotation in the other dataset, red "X" are features matched to features with wrong (different in the two datasets) annotations, red "o" are features with correctly matched annotations but deleted for being in a match considered as poor.

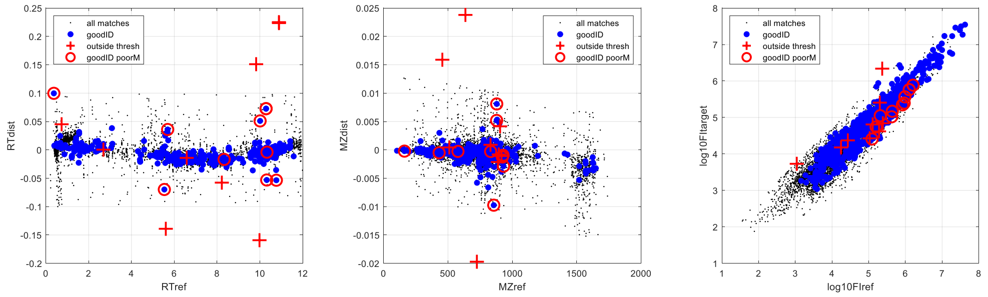

Figure SE1 17: LPOS distance plots in RT, MZ as well as log_{10}FI target vs reference, with a summary of matching results for each of the matches. For each plot, black dots represent all matches within threshold. Red "+" show matches of features that were annotated in both sets but were initially found outside of the defined thresholds, blue dots are the annotated matches with the correct (same) annotation in both datasets, red "X" are matches with wrong (different in the two datasets) annotations, red "o" are matches with correct annotations, but deleted as poor matches.

5.2.3. Comparisons of associations to covariates

The assumption here is that for true associations to defined covariates, the direction of association is the same in the two populations. Age, gender and body mass index (BMI), which distributions are shown in Figure SE1 18, were chosen because they are easy to access and are known to have many associations to metabolites. We calculated Pearson correlations (for age or BMI) or t-test (for gender, and log_{10}[fold change] between the two sexes medians) of matched features to these covariates in each dataset and compared the results for both datasets (Table SE1 3). The expected result that the associations are comparable would lead all true associations to the bottom left and top right quadrants, which is not observed when no thresholds are set (black dots in Figure SE1 19), but it is almost perfectly observed for more robust associations such as FDR and Bonferroni-level thresholds (green dots in Figure SE1 19).

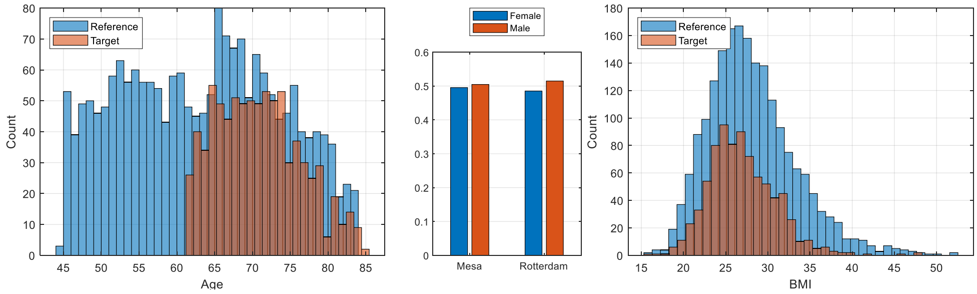

Figure SE1 18: Age, gender and BMI distributions in the MESA and Rotterdam datasets.

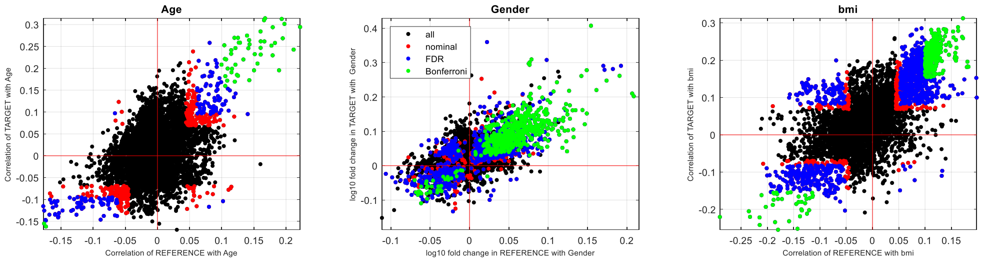

Figure SE1 19: Comparison of correlation (for age and BMI) and log_{10}(gender fold change) of all features in the target and reference datasets (black dots). Features that are significant at a specific significance level are presented in colour: red for nominal significance (p<0.05); blue for FDR significance (p<0.05); green for Bonferroni significance (0.05/6018). The plot for Gender has been zoomed for better visualisation, thus hiding some outliers (<5).

Table SE1 3: Number of features whose Pearson correlations (with age or BMI) or fold change (with gender) agrees or disagrees (has the same sign or not) in both datasets. That number was calculated for: A -all matches, no p-value threshold; B - only for matches with statistically significant coefficient/t-test with p-value at α = 0.05; C – as B, but controlling for false discovery rate (FDR) at α = 0.05 (Benjamini-Hochberg); D – as B, but controlling for family-wise error rate (Bonferroni). Notice that the level of agreement is around 60% when calculating all associations without thresholds as only a minority of variables correlate with these covariates but increases to close to 100% for the more stringent thresholds, which shows a high level of agreement.

Age	all	nominal	FDR	Bonferroni
All features	4953	471	203	49
Agreeing	2838	443	200	49
Disagreeing	2115	28	3	0
% agreeing	57.3	94.1	98.5	100.0
% disagreeing	42.7	5.9	1.5	0.0

Gender	all	nominal	FDR	Bonferroni
All features	4953	1641	1414	576
Agreeing	3403	1483	1304	574
Disagreeing	1550	158	110	2
% agreeing	68.7	90.4	92.2	99.7
% disagreeing	31.3	9.6	7.8	0.3

BMI	all	nominal	FDR	Bonferroni
All features	4953	1338	1126	290
Agreeing	3009	1181	1019	290
Disagreeing	1944	157	107	0
% agreeing	60.8	88.3	90.5	100.0
% disagreeing	39.2	11.7	9.5	0.0

5.2.4.Check if feature selection on multiple matches is correct

The npeaks parameter resulting from peak-picking using the xcms software registers the number of peaks that were found for each feature (expectedly similar to the total number of samples). In this validation strategy we assume that features detected in a higher number of samples have larger signal to noise and better quality, thus correctly matched features should be detected in more samples than incorrect matches. For each match in a cluster with 3 nodes, we computed the "npeaks" difference of [selected matches minus discarded matches]. For clusters with more than 3 features (nodes) we computed the best versus the worst match (according to the penalty scores).

Reference and target datasets contain 1958/2639 and 814/1178 biological/total samples respectively (datasets contain QC samples at time of peak picking). A much higher proportion (50 in 60, or 83%) of "npeaks" differences are positive in both reference and target (see Figure SE1 20), suggesting that the correct match was usually selected from the cluster.

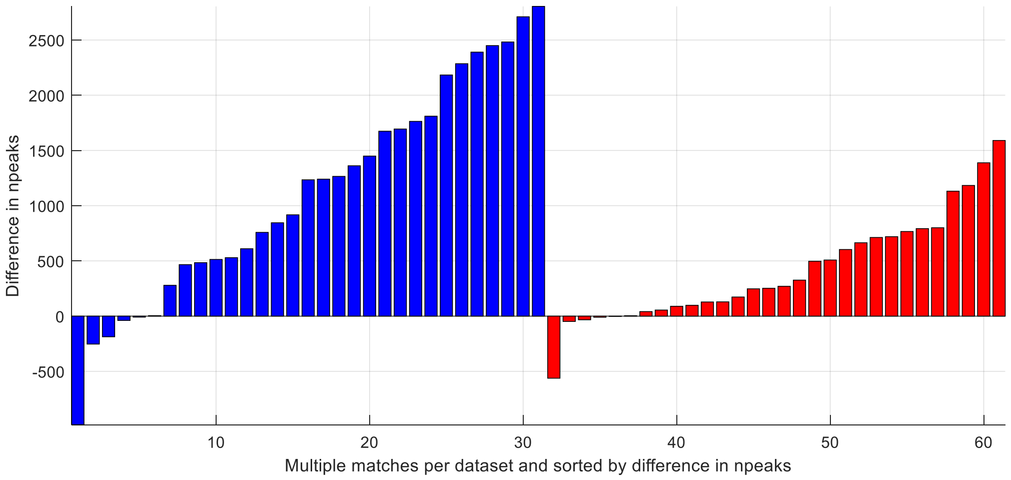

Figure SE1 20: Difference in npeaks between same-dataset features of selected and deleted matches (between multiple matches from same clusters of multiple matches) in reference (blue) and target (red) sets for each of the matches involved in a multiple match cluster. Positive values represent matches with more peaks in the features of matches that were preferred, versus features that were discarded, while negative values represent the opposite. A good outcome for the method is shown, as most matches used the feature with highest number of npeaks among the possible features in the multiple match clusters.

5.2.5. Evaluation of the number of highly correlated features with the matched features

These datasets are expected to contain adducts and isotopes, as the features have not been aggregated. Those are expected to be highly correlated among each other, and in a similar fashion for matched features in both datasets. For each matched feature, we found all features in the same dataset at a small retention time distance (< 0.25 seconds) and highly correlated (Spearman correlation > 0.7), and calculated five entities: number of within-dataset features correlated with each feature in the reference dataset; same for the target dataset; minimum number between these two values, which defines the maximum possible number of common features between matched features; number of common highly correlated features in two matched features; and the common-to-minimum number of correlated features ratio “patternScore” (1 is added to the number of minimum features to allow division when the minimum is zero). The results of this strategy are presented in Figure SE1 21.

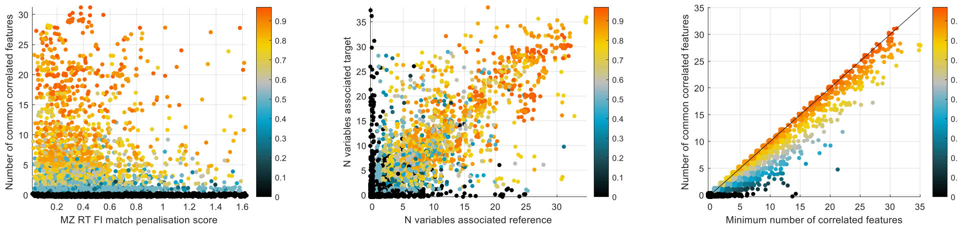

*These three plots contain only the features that survived removal of poor matches.
**Highly correlated features were defined as having Spearman > 0.7 and RT difference < 0.25 seconds.

Figure SE1 21: (left) Number of common features highly correlated** with each matched feature vs penalty scores used in the matching method (after removing poor matches). The lower the penalty score the higher the number of common correlated features; (centre) number of features highly associated (not necessarily common) with each matched feature in target vs reference; (right) Number of common correlated features vs the minimum number of correlated features (not necessarily common) between the reference or target datasets. All plots are coloured by a score obtained by the ratio common/(minimum +1).*

6. Example S2: MESA serum LNEG vs Rotterdam serum LNEG

Here we present a second example of matching, using Dataset 2. This corresponds to the same cohorts and analytical setup as Dataset 1 but was acquired in separate analytical runs and in negative ionisation mode.

6.1. Matching procedure

The complete example of the matching of features of plasma lipids in the negative mode (LNEG) MESA (reference, 6793 features) vs Rotterdam (target, 6315 features) datasets is shown below. If not mentioned in the text, figures replicate those given for Example S1.

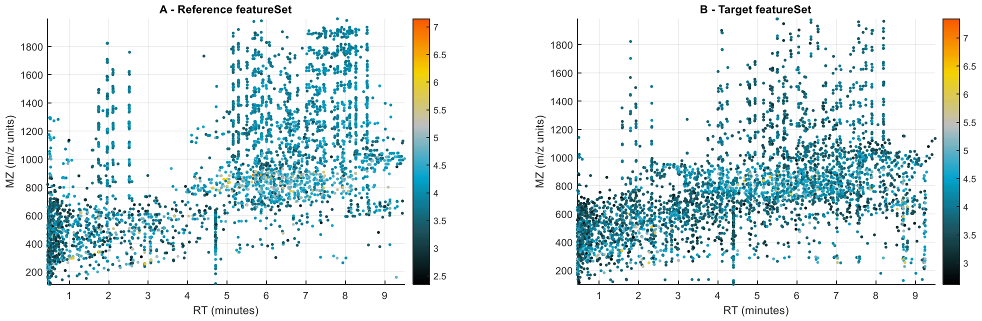

Figure SE2 1: MZ vs RT plots of LNEG datasets after RT trimming, coloured by log10FI. A: reference and B: target feature sets.

6.1.1. Step 1: Match all features within thresholds

As in example S1, initially the datasets are matched dimension using large thresholds (same as in example S1) in each dimension, so it helps visualise the trends of the inter-dataset shifts (see Figure SE2 2).

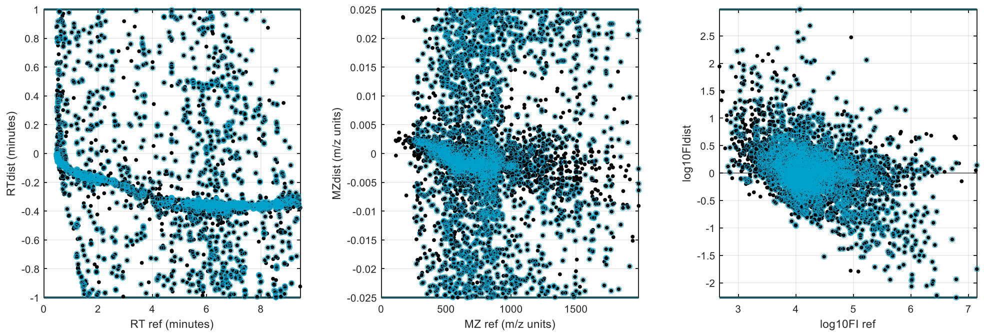

Figure SE2 2: Initial matches at large thresholds to detect major inter-dataset shift trends. The RT and MZ trends are clearly seen in the respective plots, while the FI dimension is less clear. Matches containing features involved in multiple match clusters s are shown in blue, unique matches in black.

After visual inspection of the plots in Figure SE2 2, the adjustment method for Log10FI and definition of RT/MZ/log10FI thresholds were set as:

opt.FIadjustMethod = 'regression';

```
opt.multThresh.RT_intercept = [-0.55,0.15];

opt.multThresh.RT_slope = [0, 0];

opt.multThresh.MZ_intercept = [-0.01, 0.01];

opt.multThresh.MZ_slope = [-5e-6, 5e-6];

opt.multThresh.log10FI_intercept = [-1, 1.5];

opt.multThresh.log10FI_slope = [0, 0];
```

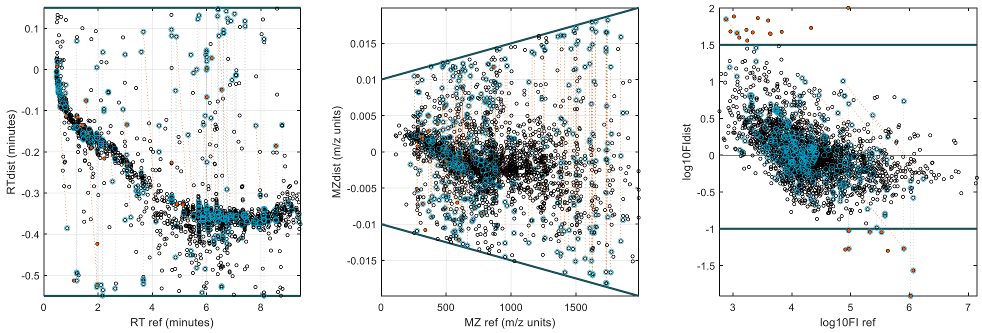

Figure SE2 3: Matches within thresholds in the RT, MZ, log10(FI) domains between LNEG datasets. (left) RT distance of target to reference; (centre) MZ distance of target to reference; (right) log10(FI) of target vs reference. Absolute thresholds are represented as horizontal lines, relative threshold as diagonal lines. Matches are represented as black dots; if part of a cluster of multiple matches their outline is emphasised in blue, and the matches are connected by thin dashed lines; if outside the log10FI thresholds they are coloured in orange.

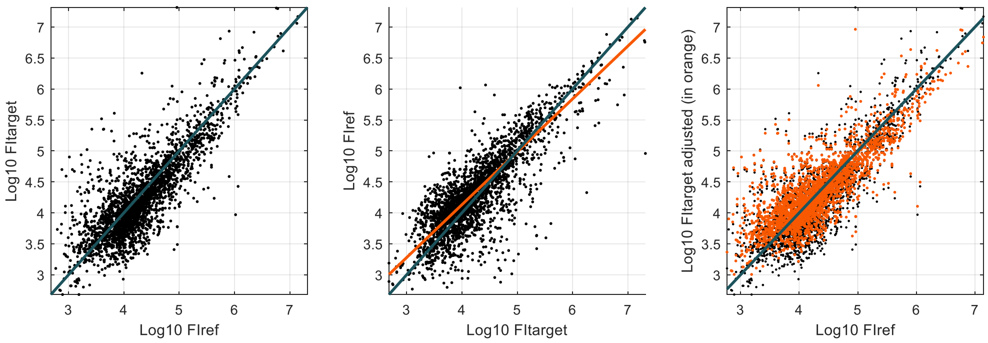

Figure SE2 4: Results of harmonization of FI in the two LNEG sets, using the 'regression' method. (left) Log10FI of target vs reference, plus line of perfect correlation; (centre) Same as left plot, plus red line with robust linear regression predictions for log10FItarget values; (right) Same as left plot, with additional orange dots representing the corrected Log10FItarget values.

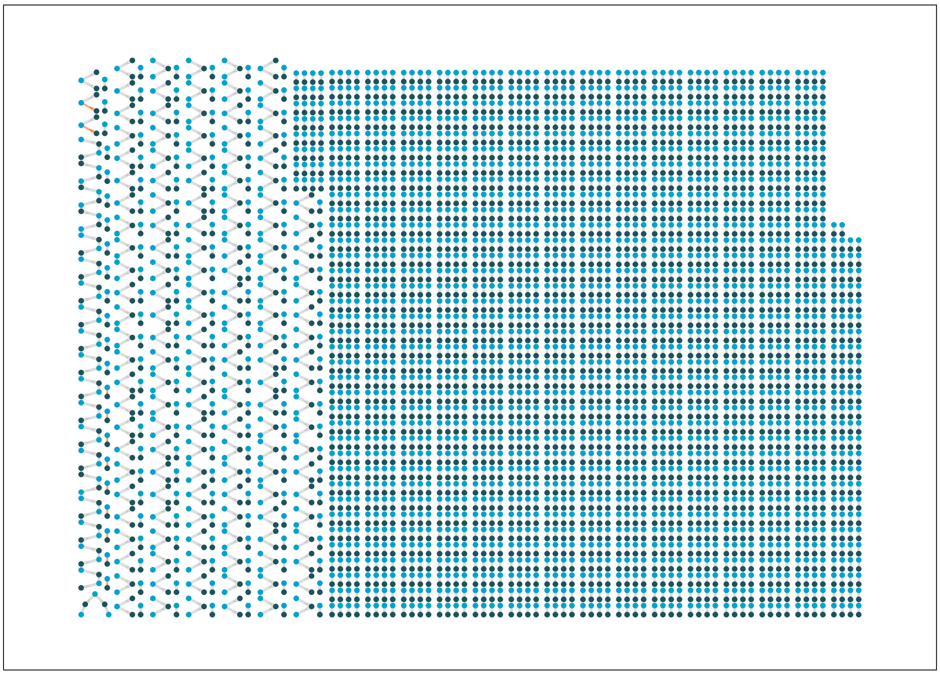

Figure SE2 5: All possible matches (edges, as lines) within thresholds between the features (nodes, as dots) of the two LNEG sets. Reference features in dark blue, target features in light blue. Orange edges represent the matches outside of log10FI thresholds.

6.1.2. Step 2: Find unique correspondence

The neighbours for the calculation of residuals were found using the following definitions:

opt.neighbours.nrNeighbors = 21;

opt.calculateResiduals.neighMethod = 'circle';

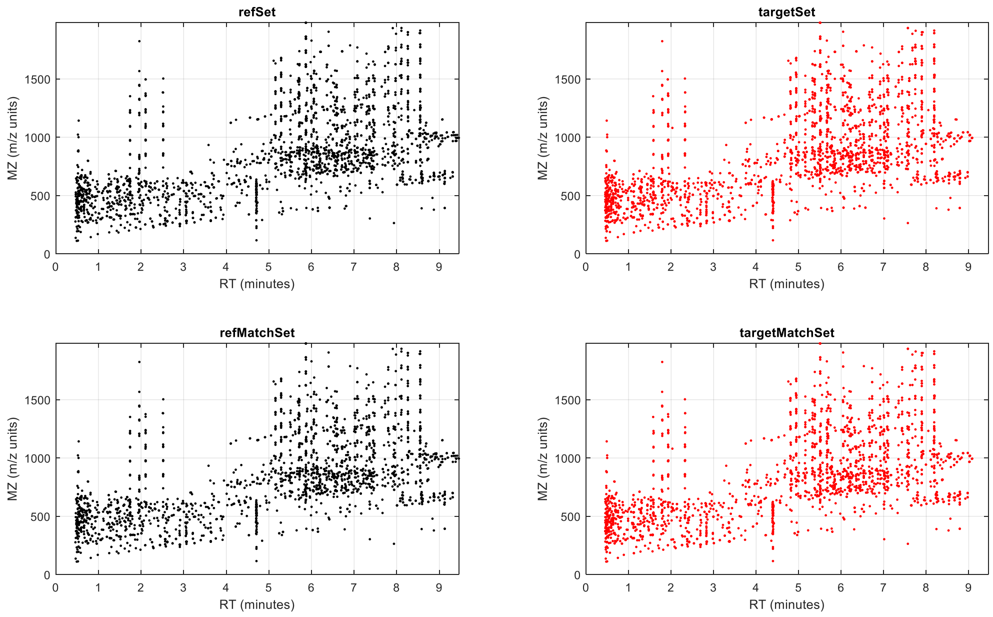

Figure SE2 6: (top) Features captured in matches within thresholds in the LNEG datasets, including the ones in multiple-match clusters; (bottom) features with only single-match possibilities used to calculate neighbours in LNEG datasets. Notice the similarity between corresponding reference and target sets (left and right figures), as well as the similarity between the sets and match sets (top and bottom figures) as most matching features only have single matches.

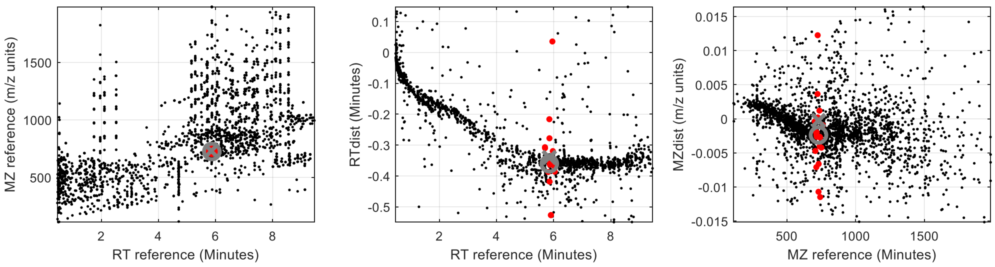

Figure SE2 7: Example of calculation of neighbours for a random feature (in this case feature with index 1323) using the 'circle' method (thus log10FI is not used). The red dots are the closest neighbours (same on the two dimensions) of the feature highlighted with a grey cross; the grey circle is the median of the neighbours indicating the inter-dataset shift trend for that dimension for that feature

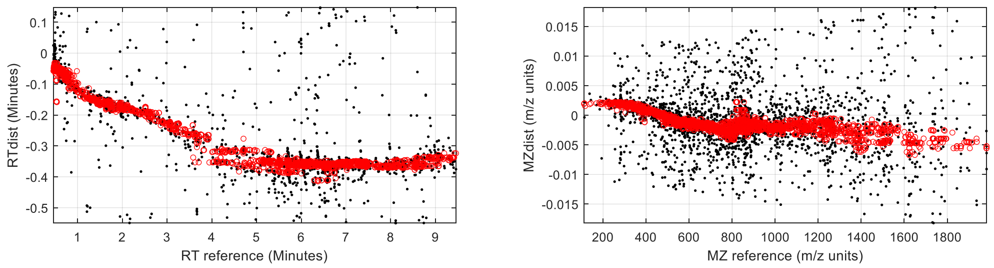

Figure SE2 8: Expected values using the "circle" method for each feature (in red) used to calculate the residuals in the RT, MZ, domains used for the calculation of the scores. This method assumes there is no inter-dataset shift in log10FI, meaning that it is equal to zero, thus in practice the residuals are calculated by direct subtraction of log10FI of the reference to the target.

.

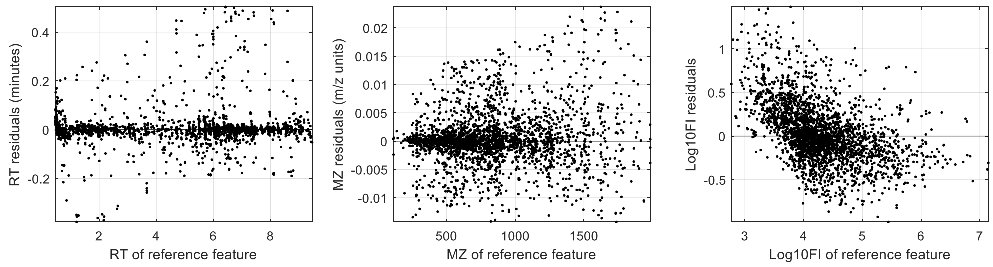

Figure SE2 9: Residuals in the three domains, to be used in the calculation of the penalisation scores for each match.

For the normalisation of residuals, the plots of Figure SE2 9 were inspected, and it was decided to manually define the values of the residuals (threshold points) that will become 1 after residualisation. Those values were [0.1, 0.01, 1.5] for RT, MZ and log10FI respectively.

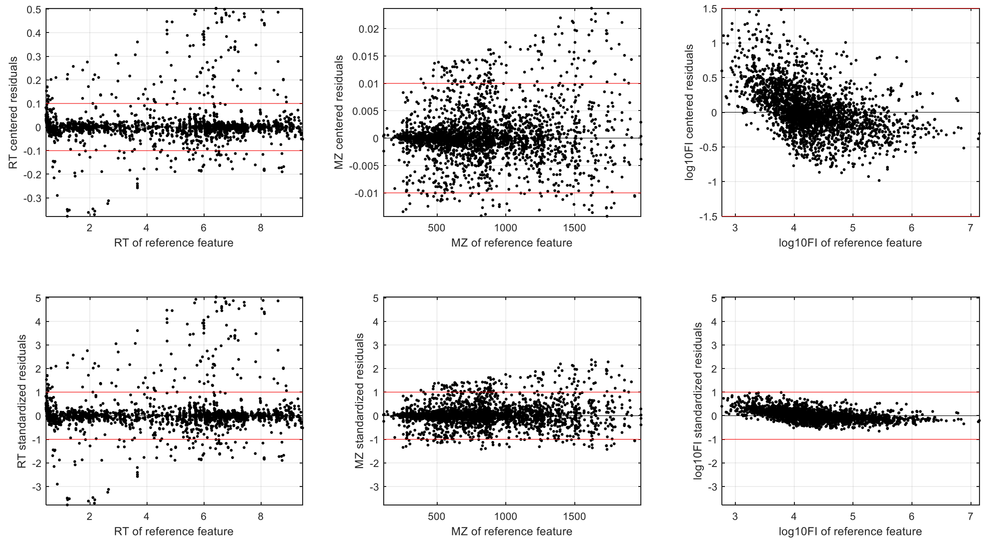

Figure SE2 10: (top) Median-centred residuals for RT/MZ/log10FI and red lines indicating the manually chosen residual values which become pivot points to be made equal to 1 for the three domains. (bottom) normalised residuals after dividing the median-centred residuals by the pivot point values selected. Notice that as the residuals are in normalised units, the bottom plots scale is the same for magnitude comparison.

The weights W were defined as [1, 1, 1], thus giving equal weight to the residuals of each dimension in the construction of the penalisation scores. Though due to the distribution of the residuals one can expect that the penalisation scores of the more extreme matches will be more influenced by the residuals of RT, then MZ and finally log10FI, as can be seen in Figure SE2 11.

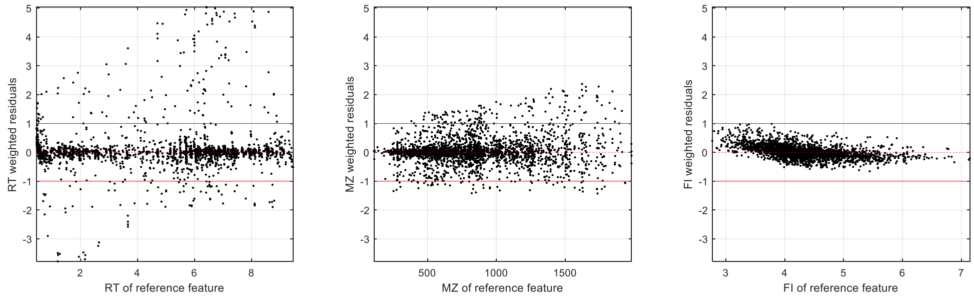

Figure SE2 11: Weighted residuals after weights were chosen to be equal in the three dimensions [1, 1, 1]. These will be used to calculate the penalisation scores (squared root of the sum of squares of the weighted residuals) in the next step. By looking at the plots one can understand that in this case RT is the most relevant dimension in the calculation of the penalisation score.

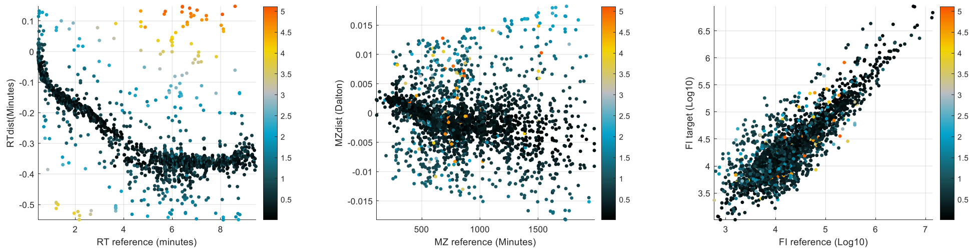

Figure SE2 12: Matches in the RT, MZ, log10(FI) domains between LNEG datasets. (left) RTdist of target to reference; (centre) MZdist of target to reference; (right) log10(FI) of target vs reference. All plots are coloured by match penalisation scores created from the normalised residuals.

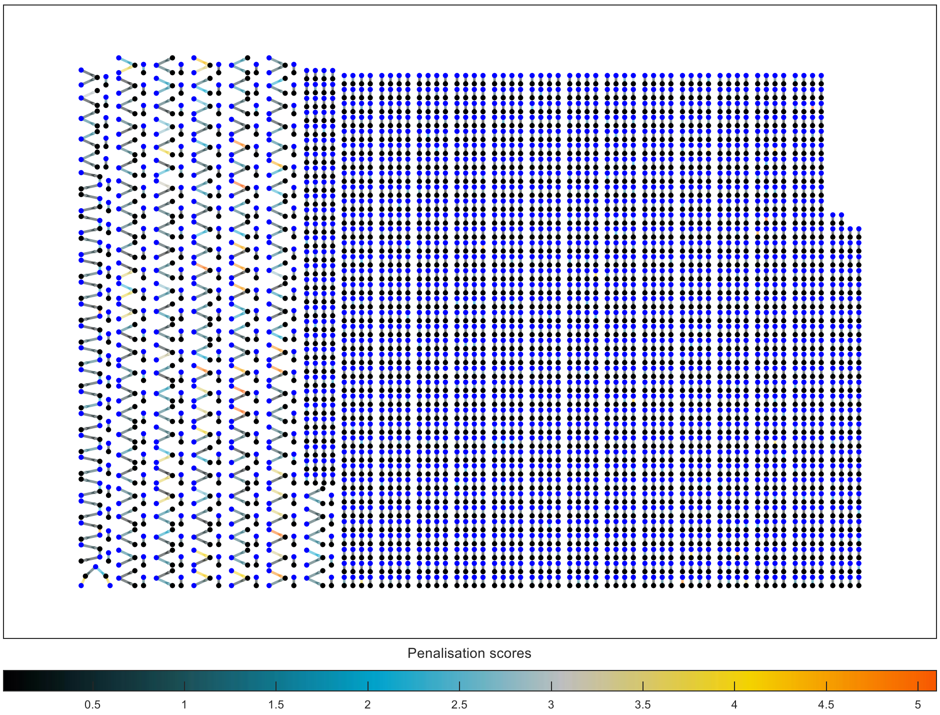

Figure SE2 13: Network with all matches (edges) coloured by penalty scores. Metabolomic features (nodes) of reference in black, target features in blue.

6.1.3.Step 3: Detect poor matches (tighten thresholds)

The method chosen to detect matches at extreme distances from the trends was “trend_mad”. Using this method, the inter-dataset shift trends were recalculated in each dimension using only the final matches, and thresholds were set using a factor of 5 median absolute deviations.

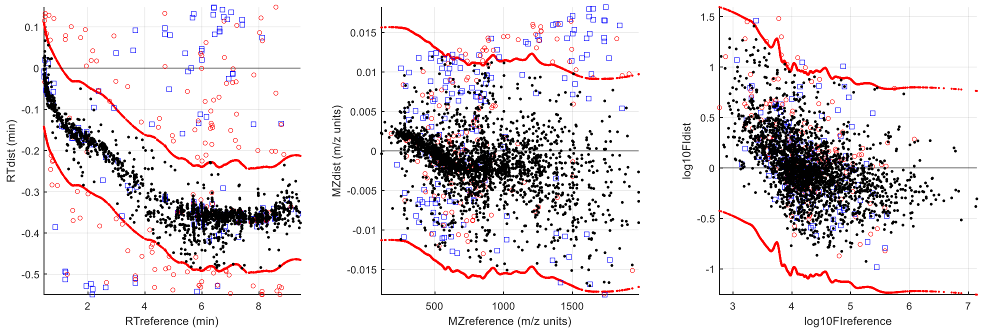

Figure SE2 14: Tightening of thresholds (red lines) used to define poor matches using the method ‘trend_mad’, with 5 MAD. Inter-dataset distance plots showing good matches as black dots, matches not selected from clusters as blue squares, poor matches as red circles

The numbers of features and matches during each stage of the process are presented in Figure SE2 15 and Table SE2 1.

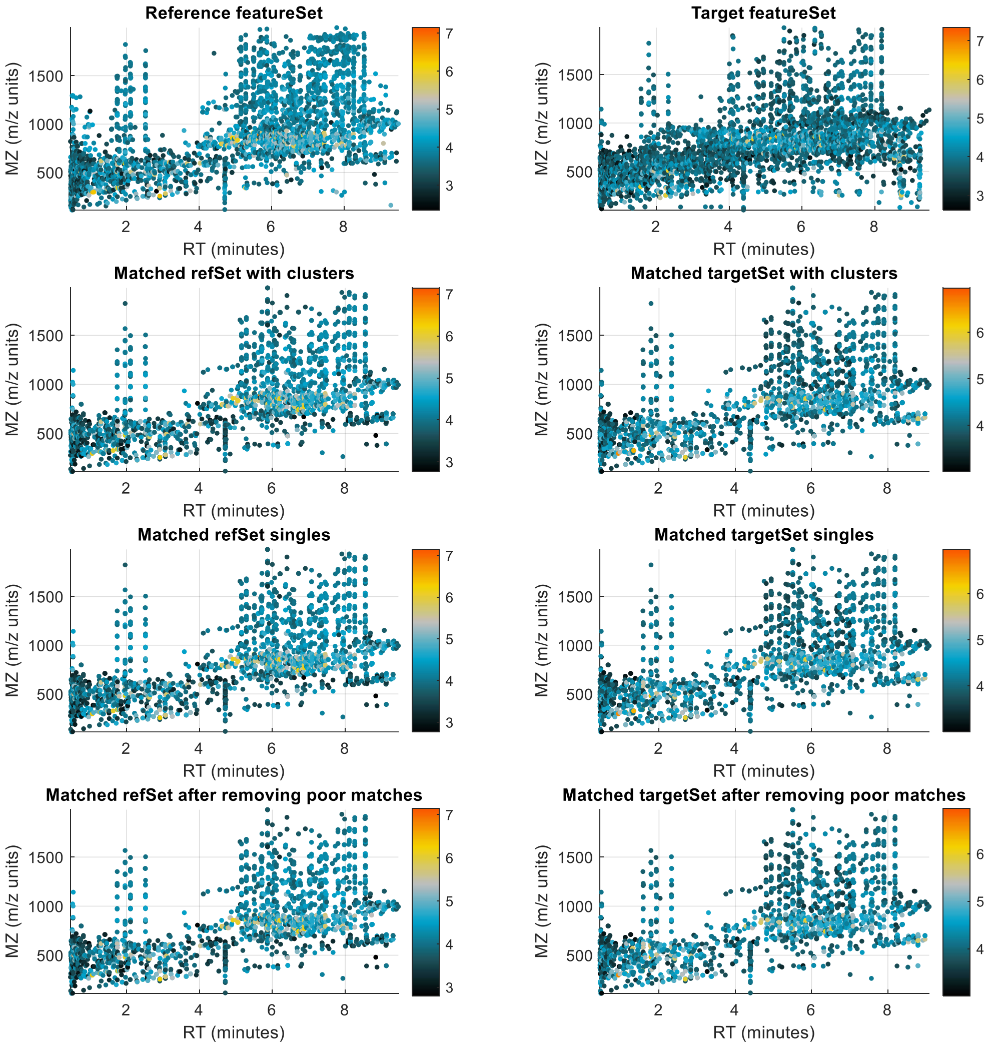

Figure SE2 15: MZ vs RT plots of LNEG datasets at each stage of the process (top to bottom) coloured by penalisation scores.

Table SE2 1: Summary of number of matches, features and clusters of multiple matches in the network. There are 2646 total matches, most of them unique (2308 clusters of matches with only 2 nodes, when both features only match to each other). The network of these matches produces 2467 clusters with 2-5 features each, which after recursive division end up yielding a total of 2486 unique matches. Excluding poor matches (by tightening thresholds) results in 2324 matches.

**Total matches**	2646
Reference features	2547
Target features	2566
**Features in only one match**	
In Reference	2448
In Target	*2486*
**Features with multiple matches**	
In Reference	*99*
In Target	*80*
**Clusters of matches**	2467
2 nodes	2308

3 nodes	140
4 nodes	18
5 nodes	1
**Unique matches including poor**	2486
**Unique matches without poor**	2324

6.2. Validation

6.2.1. Comparison of FI

The composition of plasma is highly regulated thus the median concentration of a metabolite should be on a similar order of magnitude in both sets. Although from different populations, the sample type, extraction, injection and peak-picking methods were similar, and we observe that peak size in both sets shows good agreement in a log_{10}FI scale for most features (plot on the right, Figure SE2 12 and Figure SE2 17).

6.2.2. Comparison of metabolite annotations

Table SE2 2: Number of annotated matches in the data at each stage. There are 87 annotations in the initial data of both Reference (6793 features) and Target datasets (6315 features). There are 3 annotated features in each set that could match, but their matches are outside of the defined initial thresholds. After setting thresholds for multiple matching only around 40% of the features in the datasets match to each other (2646 matches). After unique matching (2486 matches) 82 of the annotated features are found with correct ID in both datasets, while for 2 of them the ID is different. Regrettably, after deleting poor matches (ending in 2324 matches) 3 correctly annotated matches are deleted, ending with a total of 79 (90.8%) correct and 8 (9.2%) incorrect/not found matches.

Stage and results	Number annotations	Number matches
Initial data	**87**	(6793/6315 unmatched features)
Matches outside thresholds		**3**
After all matches within thresholds (step1)	**84**	2646
After unique matches (step 2)	**84**	2486
Correct ID matches	**82**	**-**
Wrong ID matches	**2**	**-**
After removing poor matches (step 3)		2324
Final number of correct ID matches	**79**	
Final number of wrong ID/outside threshold matches	**8**	

Poor matches	**3**	162
With correct ID	**3**	-
With wrong ID	**0**	-

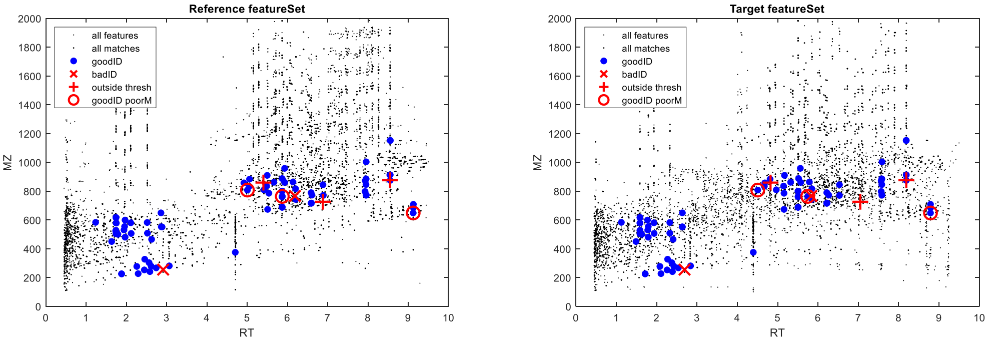

Figure SE2 16: MZ vs RT of reference (left) and target (right) LNEG datasets, with a summary of matching results for each of the metabolomic features. For each plot, small black dots represent all features in the dataset, while larger black dots represent features that were matched. Red "+" show features that were initially found outside of the defined thresholds, blue dots are the annotated features matching a feature with the correct (same) annotation in the other dataset, red "X" are features matched to features with wrong (different in the two datasets) annotations, red "o" are features with correctly matched annotations but deleted for being in a match considered as poor.

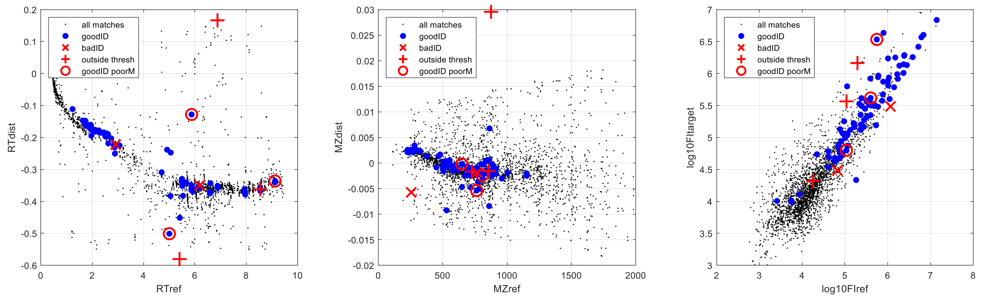

Figure SE2 17: LNEG distance plots in RT, MZ as well as $log_{10}FI$ target vs reference, with a summary of matching results for each of the matches. For each plot, black dots represent all matches within threshold. Red "+" show matches of features that were annotated in both sets but were initially found outside of the defined thresholds, blue dots are the annotated matches with the correct (same) annotation in both datasets, red "X" are matches with wrong (different in the two datasets) annotations, red "o" are matches with correct annotations, but deleted as poor matches.

6.2.3. Comparison of associations to covariates

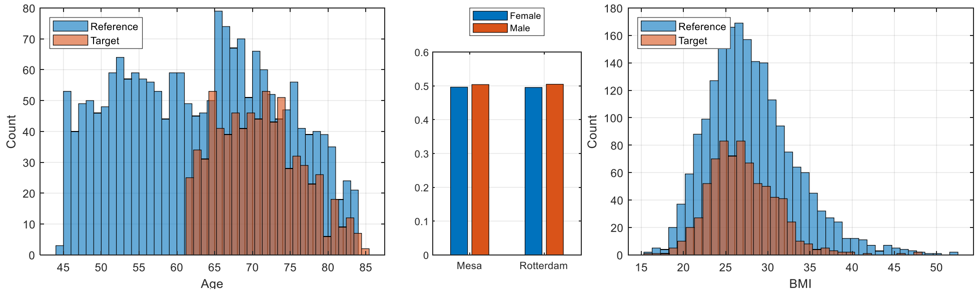

Figure SE2 18: Age, gender and BMI distributions in the MESA and Rotterdam datasets.

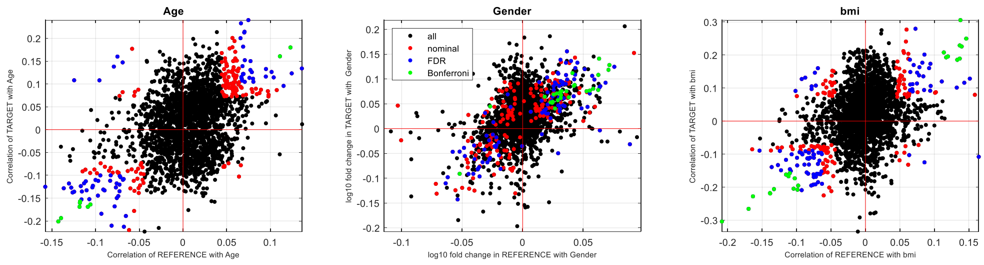

Figure SE2 19: Comparison of correlation (for age and BMI) and log10(gender fold change) of all features in the target and reference datasets (black dots). Features that are significant at a specific significance level are presented in colour: red for nominal significance (p<0.05); blue for FDR significance (p<0.05); green for Bonferroni significance (0.05/2079). The plot for Gender has been zoomed for better visualisation, thus hiding some outliers (<10).

Table SE2 3: Number of features whose regression coefficient (with age or BMI) or fold change (with gender) agrees or disagrees (same sign or not) in both datasets. That number was calculated for: A -all matches, no p-value threshold; B - only for matches with statistically significant coefficient/t-test with p-value at α = 0.05; C – as B, but controlling for false discovery rate (FDR) at α = 0.05 (Benjamini-Hochberg); D – as B, but controlling for family-wise error rate (Bonferroni). Notice that the level of agreement is close to 60% when calculating all associations without thresholds as only a minority of variables correlate with these covariates but increases to nearly 100% for more stringent thresholds, showing a high level of agreement.

Age	all	nominal	FDR	Bonferroni
All features	2324	210	79	9
Agreeing	1392	193	74	9
Disagreeing	932	17	5	0
% agreeing	59.9	91.9	93.7	100.0
% disagreeing	40.1	8.1	6.3	0.0

Gender	all	α = 0.05	FDR	Bonferroni
All features	2324	285	126	31
Agreeing	1410	237	117	30
Disagreeing	914	48	9	1
% agreeing	60.7	83.2	92.9	96.8
% disagreeing	39.3	16.8	7.1	3.2

BMI	all	α = 0.05	FDR	Bonferroni
All features	2324	218	104	22
Agreeing	1389	180	93	22
Disagreeing	935	38	11	0
% agreeing	59.8	82.6	89.4	100.0
% disagreeing	40.2	17.4	10.6	0.0

6.2.4. Check if feature selection on multiple matches is correct

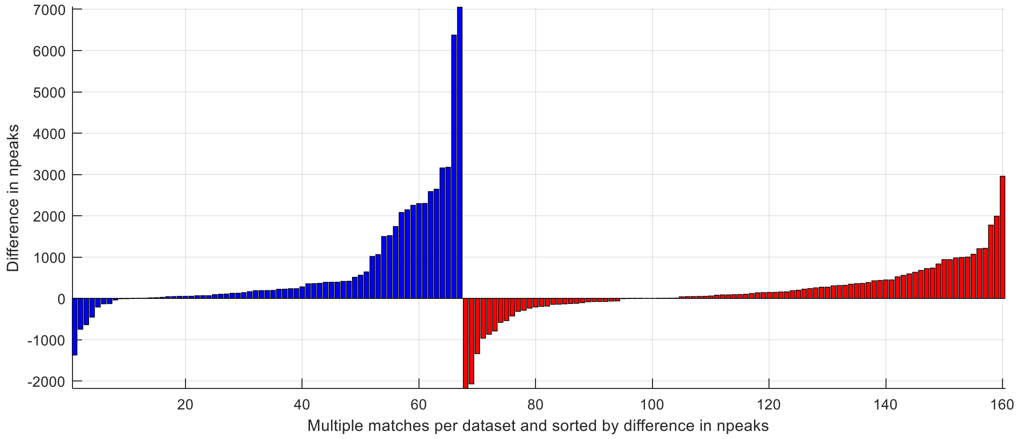

Figure SE2 20: Difference in npeaks between same-dataset features of selected and deleted matches (between multiple matches from same clusters of multiple matches) in reference (blue) and target (red) sets for each of the matches involved in a multiple match cluster. Positive values represent matches with more peaks in the features of matches that were preferred, versus features that were discarded, while negative values represent the opposite. A good outcome for the method is shown, as most matches used the feature with highest number of npeaks among the possible features in the multiple match clusters.

6.2.5. Evaluation of the number of highly correlated features

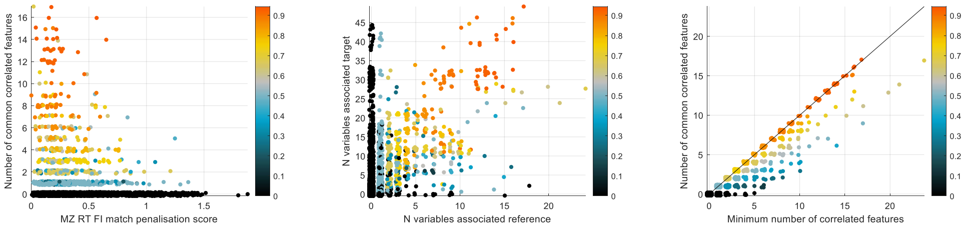

*These three plots contain only the features that survived removal of poor matches.

**Highly correlated features were defined as having Spearman > 0.7 and RT difference < 0.25 seconds.

Figure SE2 21: (left) Number of common features highly correlated** with each matched feature vs penalty scores used in the matching method (after removing poor matches). The lower the penalty score the higher the number of common correlated features; (centre) number of features highly associated (not necessarily common) with each matched feature in target vs reference; (right) Number of common correlated features vs the minimum number of correlated features (not necessarily common) between the reference or target datasets. All plots are coloured by a score obtained by the ratio common/(minimum +1).*

7. Example S3: Step-by-step analysis of synthetic data

7.1. Data

Two synthetic datasets were prepared by adding systematic and random variation into features of a real dataset (the MESA negative mode dataset), and the two were then matched. The synthetic datasets were prepared in order to have similar variation and inter-dataset shifts to the MESA vs Rotterdam negative mode datasets described in example S2. To simplify the analysis, FI is not used to define penalisation scores ($W_{FI} = 0$), thus the FI dimension is irrelevant for the matching. The synthetic dataset was prepared according to the following: systematic RT (in minutes) and MZ (in Dalton) shifts were created, according to

$$\text{SystematicShift}_i = \text{offset} + \text{maxShift} \cdot \sin \theta_i$$

, where θ_i was defined for each of the N (= number of features) equal increments in a specified interval, multiplied by a maximum shift factor, and added of an offset value. For RT, θ_i was defined in [0, π /2], with maxRTshift = -0.4 and offset = 0. For MZ the parameter θ_i was defined in [0, π /2], with maxMZshift = -0.005 Dalton and offset = 0.0025.

Random variation (noise) was also created to be added for both RT and MZ. For RT, it was defined as

$$\text{RTnoise}_i = \text{RTnoisefactor} \cdot \text{randomShiftRT},$$

with RTnoisefactor = 0.02 (in minutes) and by sampling randomShiftRT from a uniform distribution defined in [-1,1]. For MZ the noise was proportional to the MZ_i and was defined as

$$\text{MZnoise}_i = (\text{MZnoisefactor} \cdot \text{MZ}_i) \cdot \text{randomShiftMZ},$$

with MZnoisefactor = 4×10^{-6} (in Daltons) and by sampling randomShiftMZ from a uniform distribution defined in [-1,1].

The systematic and random variations were added to each of the dimensions after these were sorted, resulting in a smooth shift along the RT and MZ as seen in the original datasets (see Figure SE3 3), according to:

$$RTsynthetic_i = sortedRT_i + SystematicRTshift_i + RTnoise_i$$

$$MZsynthetic_i = sortedMZ_i + SystematicMZshift_i + MZnoise_i$$

The sources of variation added to the LNEG features are presented in Figure SE3 1, together with the distances plots. Notice that the FI are the same so they are not presented.

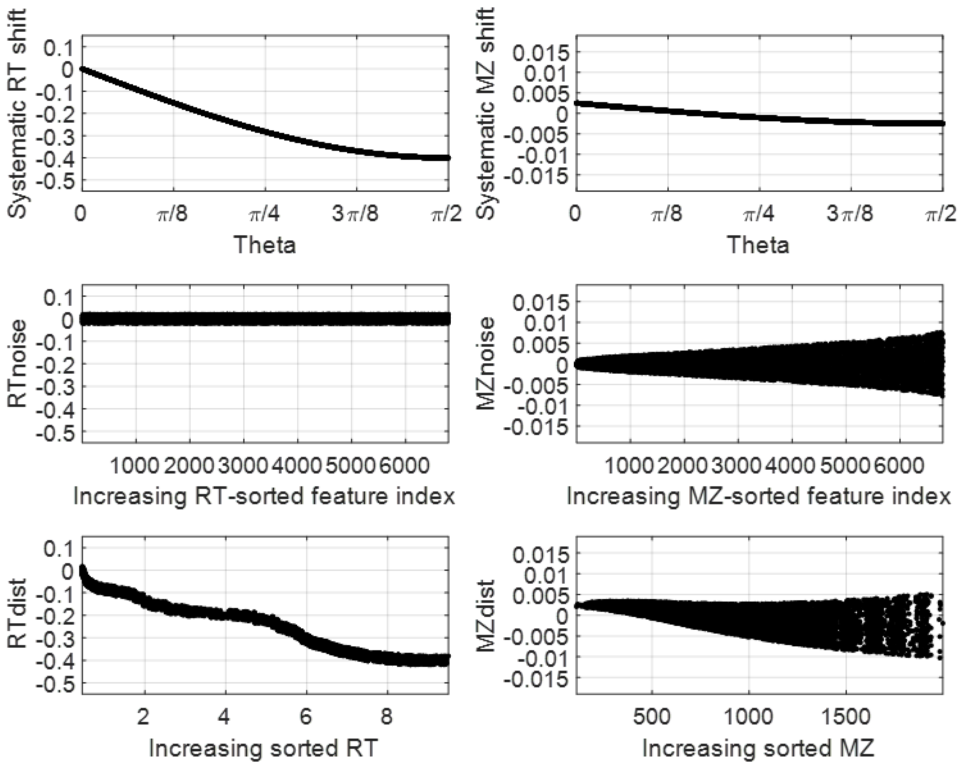

Figure SE3 1: Systematic and random variation to add to RT and MZ of the MESA negative mode dataset to create an artificial dataset. (top left) Systematic RT shifts to add to the sorted RT; (top right) Systematic MZ shifts to add to the sorted MZ. (centre left) Random variation to add to the sorted RT. (centre right) Proportional random variation to add to the sorted MZ. (bottom left) The RT difference between synthetic and original data as a function of the original data's sorted RT. (bottom right) The MZ difference between synthetic and original data as a function of the original data's sorted MZ.

At this point, for each feature in the original dataset there is a single corresponding one in the synthetic dataset, thus all features are paired. In order to create additional differences in the original and synthetic datasets a block 30% of the features is deleted in the reference dataset, and a different block with 30% of the features is deleted in the target. Thus, each of the datasets contains now 40% of the same features and 30% unpaired ones (randomly chosen), in a total of 4755 features each (each dataset has only 70% of the 6793 in initial LNEG). If only the designed matches would match, we should be able to match correctly 0.4*6793 = 2717 features.

7.2. Procedure

For comparison with the artificial datasets, the initial real LNEG datasets looked like:

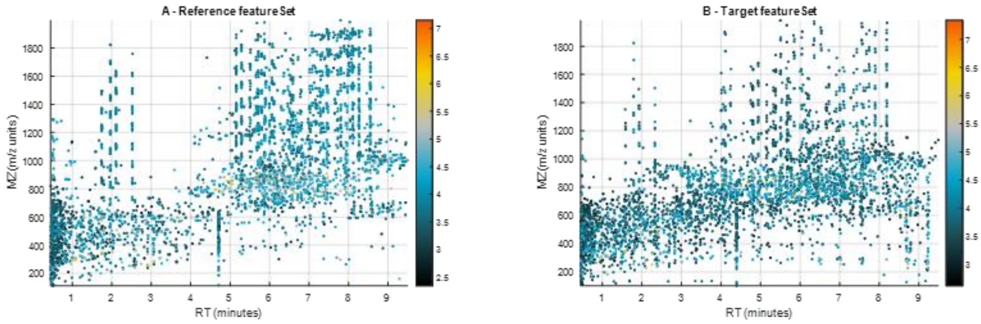

Figure SE3 2: initial LNEG real features

And the real LNEG distances plots looked like:

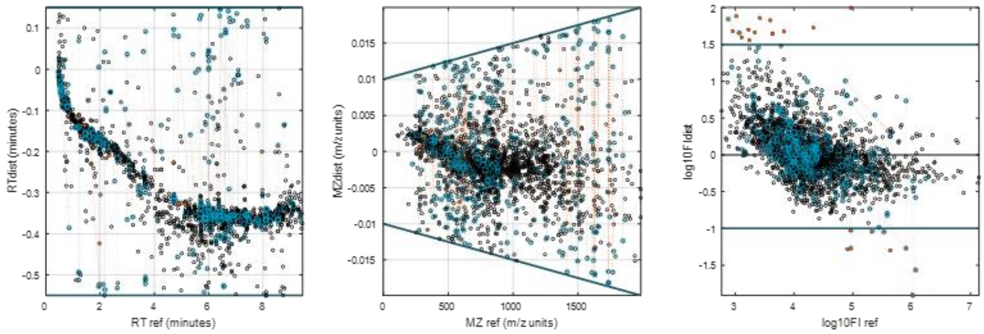

Figure SE3 3: Distance plots for the real LNEG datasets

The MZ vs RT plots for the MESA reference and (target) artificial dataset are presented below. Notice that the reference dataset features are the same as in the initial MESA negative mode dataset, though only 70% of them make part of this dataset. The artificial target dataset also contains only 70% of the variables of MESA (with added variance), as previously described.

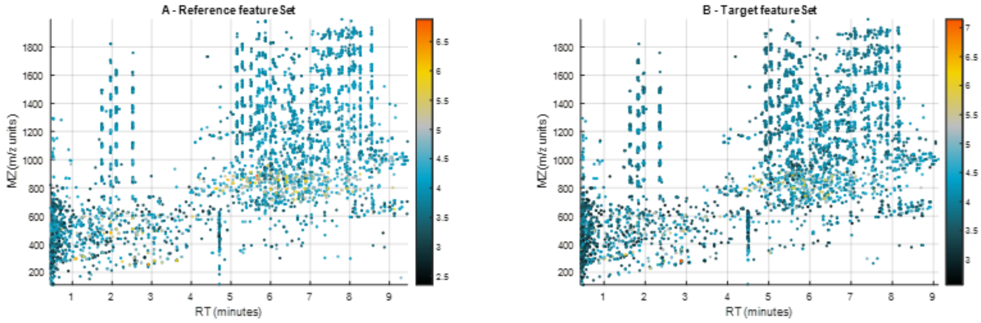

Figure SE3 4: the artificial datasets features

The same initial thresholds as in Example S2 were set for the RT and MZ dimension. The FI dimension is not used to simplify the analysis (we will set later $W_{FI} = 0$), so the thresholds are irrelevant. The distance plots for the artificial sets matches within thresholds are:

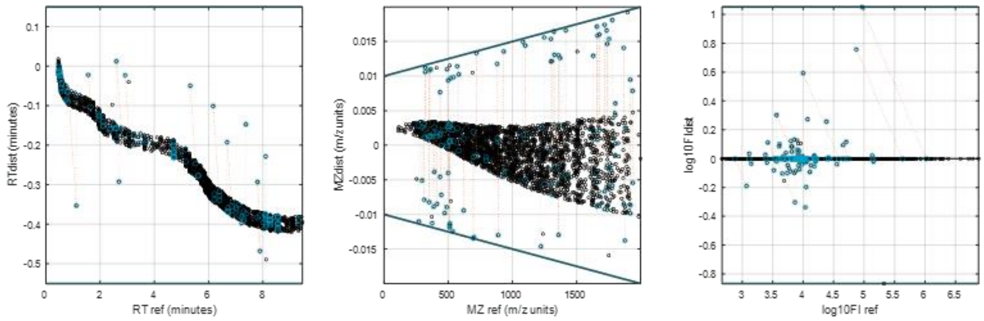

Figure SE3 5: Distance plots for all matches between the artificial datasets

The matches represented as a network are:

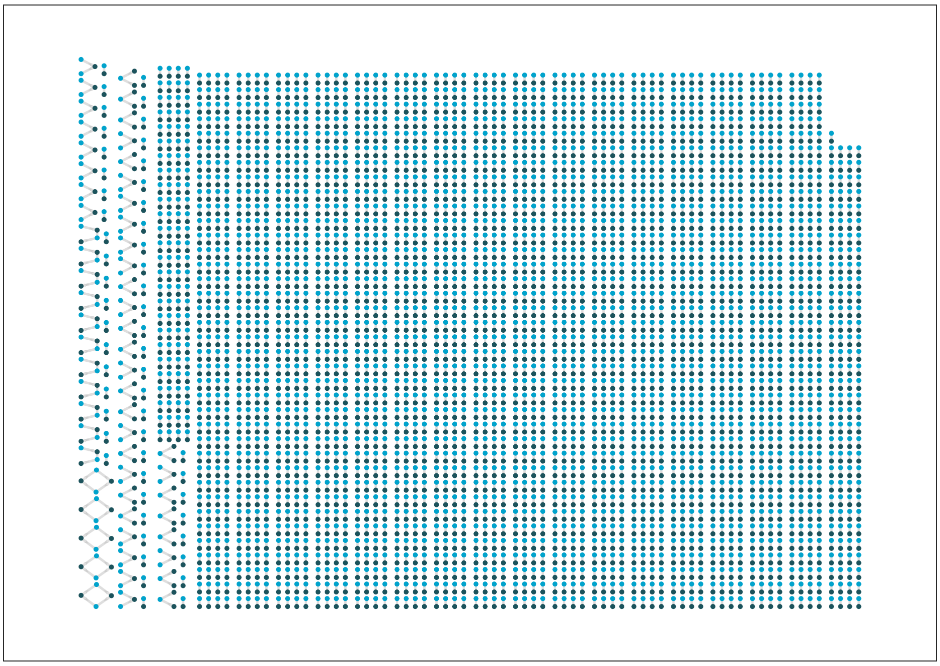

Figure SE3 6: Network with all matches (edges). Metabolomic features (nodes) of reference in black, target features in blue.

The “circle” method using 21 neighbours was applied to find inter-dataset shifts and residuals. The inter-dataset shifts for RT, MZ are presented in Figure SE3 7:

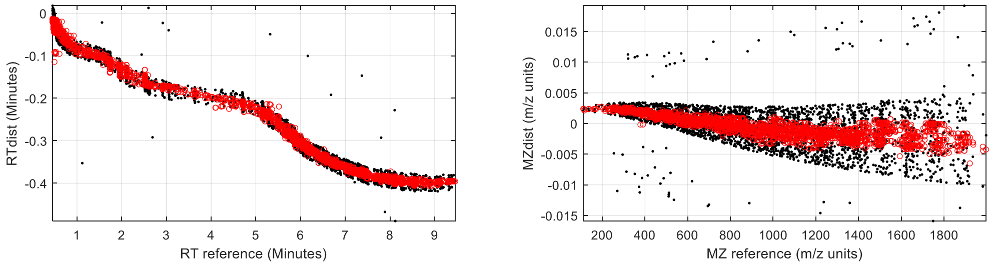

Figure SE3 7: Trends for RT and MZ in the artificial datasets

The threshold values used to normalise the residuals were [0.1,0.01,1.5]. The weights were defined as W = [1, 1, 0], thus FI does not enter in the calculation of the scores. The weighted residuals are shown in Figure SE3 8:

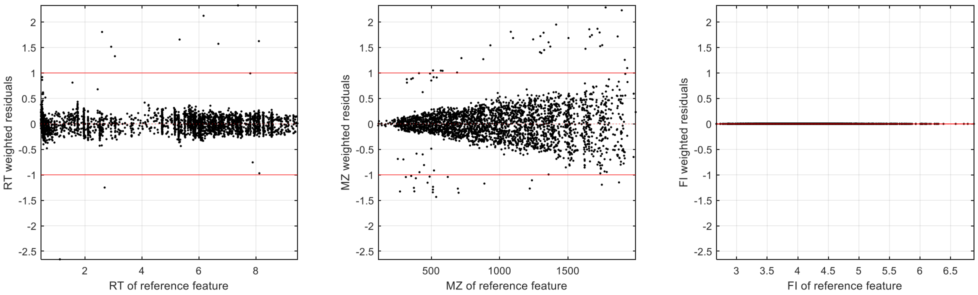

Figure SE3 8: Weighted residuals of the artificial datasets

The distance plots coloured by scores are presented in Figure SE3 9:

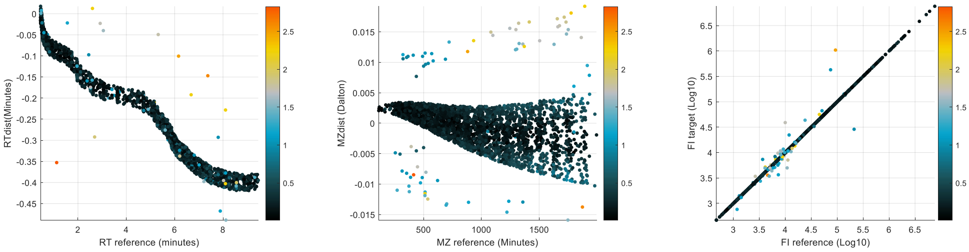

Figure SE3 9: Distance plots with matches coloured by penalisation scores.

All matches within thresholds represented as a network, edges coloured by penalisation scores:

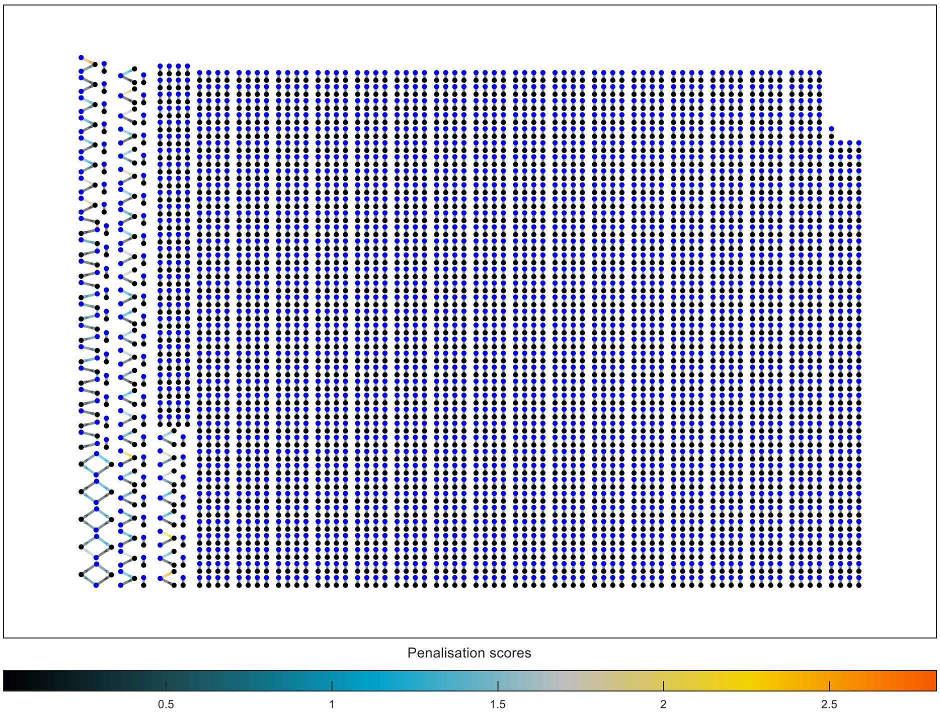

Figure SE3 10: Network with all matches (edges) coloured by penalty scores. Metabolomic features (nodes) of reference in black, target features in blue.

The poor matches were found using the “scores” method at 5 MAD, and are presented in Figure SE3 11:

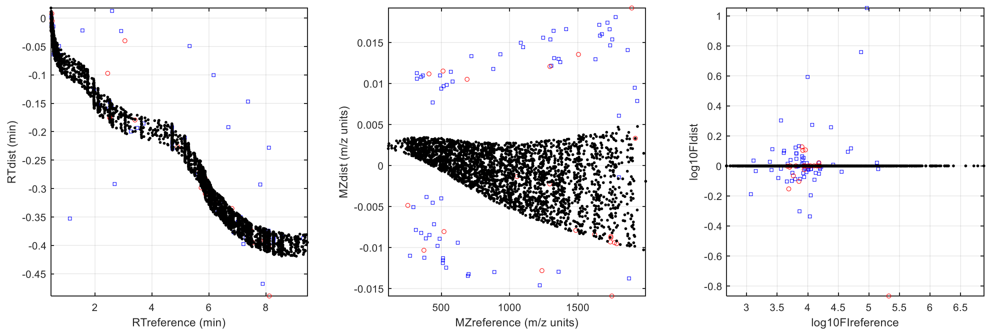

Figure SE3 11: Tightening of thresholds used to define poor matches (in red) using the method ‘scores’, at 5 dMAD. Residuals plots showing poor matches in red and true positive matches in black, as well as the features from clusters that were not selected in blue.

We expected to find 2717 matches, while 2712 clusters were found after step 1. From these, after step 2, 2728 were unique matches. Notice that there are more unique matches than expected ones, as additional matches not accounted by the dataset design may happen by chance. After detection and deletion of poor matches (step 3), the number reduced to 2707 (99.63%), a result very close to the expected one.

8. Example S4: AD plasma LPOS vs Airwave plasma LPOS

This example has three objectives:

- Show the application of our method to **lipidomics datasets with large retention time differences**

- Compare the M2S methodology and results with another method, metabCombiner [11], **without using annotations** to assist in the matching

- Add practical information about the use of some functions and strategy for matching using M2S

These two plasma lipidomics datasets were acquired by different research groups. The AD samples underwent modified Folch lipid extraction, while Airwave did not. AD was acquired using ion mobility, while Airwave was not. The chromatograms were acquired in **different instruments** (both in ESI positive mode) using **very different elution gradients**, yielding non-linear, **large retention time differences** (up to 6 minutes in a total of 18 minutes), as well as **significant m/z systematic difference**. The peaks in each sample were detected, integrated and assembled into a single table using **different software** packages (MassHunter Workstation suite for AD and XCMS for Airwave). For this article the median values of RT, MZ, FI were then obtained/calculated for each of the datasets, and the datasets were matched.

8.1. Matching using metabCombiner

We aimed to replicate the experience of a regular user, accessing both the article and an online tutorial for information. For the R session we followed the online tutorial posted at [12], and the function calls and discussion in this section can be better understood by following that webpage.

We used the default 'binGapValue' of 0.005 m/z, to combine the features into groups (or clusters), obtaining 311 groups of features. Then we used the settings below to define the anchors. Anchors are the inter-dataset-matched highest intensity features in each dataset within retention time and m/z delimited windows. This methodology forces the feature intensity to be comparable in both sets, thus should only work in datasets of the same biological fluids.

p.combined.2 = selectAnchors(p.combined, windx = 0.025,windy = 0.025, tolQ = 10, tolmz = 0.0075, useID = FALSE)

We modelled the inter-dataset retention time shift using the Generalized Additive Model with the following settings:

set.seed(100)

p.combined.3 = fit_gam(p.combined.2, useID = FALSE, k = seq(12,20,2), iterFilter = 2, coef = 2, prop = 0.5, bs = "bs", family = "gaussian", m = c(3,2))

The only plot supplied by the metabCombiner package is the following:

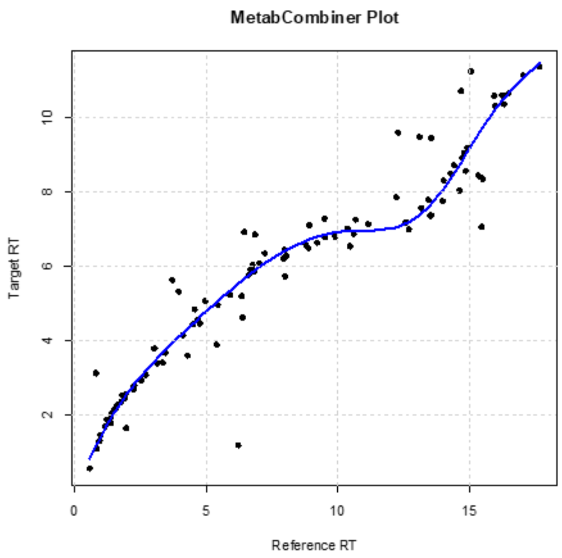

Figure SE4 1: metabCombiner scatter plot of RT of target vs RT of reference. The black dots are the anchor matches, and the blue line is the modelled inter-dataset shift.

We then calculated the scores by adjusting the weights as:

p.combined.4 = calcScores(p.combined.3, A = 85, B = 13, C = 0.5, usePPM = FALSE, useAdduct = FALSE, groups = NULL)

The results of the matching were collected in two ways, one containing a table with all matches (including conflicts), and another forcing a decision on the conflicts, yielding only one-to-one matches, which could be compared directly with the results of our method.

The function calls to obtain all matches were:

combined.table.byMZRT = labelRows(combined.table, minScore = 0.5, maxRankX = 3, maxRankY = 3, method = "mzrt", balanced = TRUE, delta = c(0.005,0.5,0.005,0.5))

The instructions for obtaining only one-to-one matches were:

combined.table.finalReport = reduceTable(combined.table, minScore = 0.5, maxRankX = 3, maxRankY = 3)

After these steps the matching of the two datasets using metabCombiner was complete.

8.2. Matching using M2S

We loaded the data and created unique MZRT string identifiers representing each of the features.

```
[refFeatures] = importdata(refFilename);
[targetFeatures] = importdata(targetFilename);

[refMZRT_str] = M2S_createLabelMZRT('ref',refFeatures(:,2),
refFeatures(:,1));
[targetMZRT_str] = M2S_createLabelMZRT('target', targetFeatures(:,2),
targetFeatures(:,1));
```

The two datasets are presented in the figures below.

```
M2S_figureH(0.8,0.5)
subplot(1,2,1),
M2S_plotMZRT_featureSet(refFeatures,1,8,1); title('Reference featureSet')
subplot(1,2,2),
M2S_plotMZRT_featureSet(targetFeatures,1,8,1); title('Target featureSet')
```

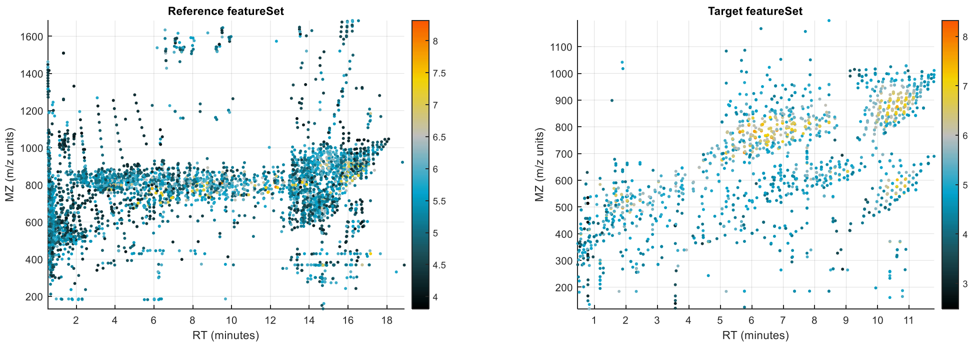

Figure SE4 2: MZ vs RT plots of plasma positive mode LC-MS lipidomics of experiments AD (left) and Airwave (right), coloured by log10FI

We started by matching the two datasets using large thresholds (function “M2S_matchAll.m” with default settings: no RT threshold, MZ threshold = 0.02 Da, no FI adjustment neither and no FI threshold). This allowed one to start understanding the inter-dataset shifts. We then had a look at the matches, and by colouring them by delta MZ one could see retention time difference trends clearly (greyish points in figure below). For these thresholds, one can use the function M2S_matchAll with default settings as below. We used the following code:

```
[refSet_i,targetSet_i,Xr_connIdx_i,Xt_connIdx_i,opt_i]=M2S_matchAll(
refFeatures,targetFeatures)
M2S_figureH(0.8,0.4)
M2S_plotDelta_matchedSets(refSet_i,targetSet_i,'.k')
M2S_colorByY_ofSubplot(2,gcf)
```

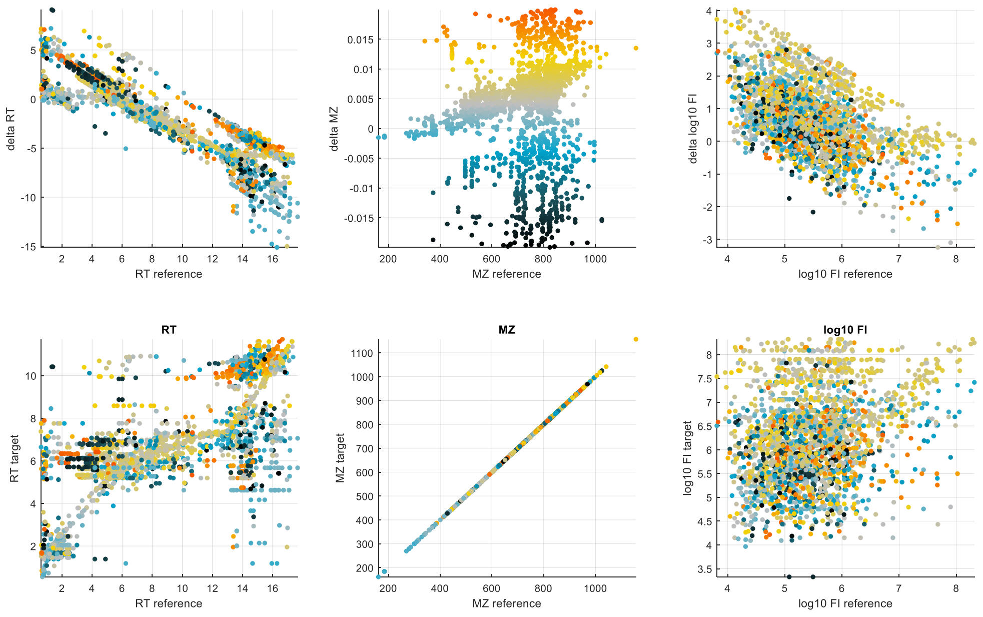

Figure SE4 3: Plot of matches using function "M2S_plotDelta_matchedSets.m" and "M2S_colorByY_ofSubplot.m". These functions help at visualising the inter-dataset shifts in more complex cases. Top row: differences vs reference values for each of the dimensions. Bottom row: target vs reference values for each of the dimensions.

Additionally, selecting only small clusters of matches and disregarding the larger ones, allows one to have a better understanding of the expected inter-dataset differences, even using these large thresholds. We used the function "deleteLargeClusters .m" to visualise only clusters with 2 features (meaning only 1-to-1 matches) with the following code:

```
maxFeaturesInCluster=2;
[refFeatures_noBigClusters,targetFeatures_noBigClusters,Xr_connIdx_i,
Xt_connIdx_i] = M2S_deleteLargeClusters(refSet_i,targetSet_i
,maxFeaturesInCluster, opt_i);
```

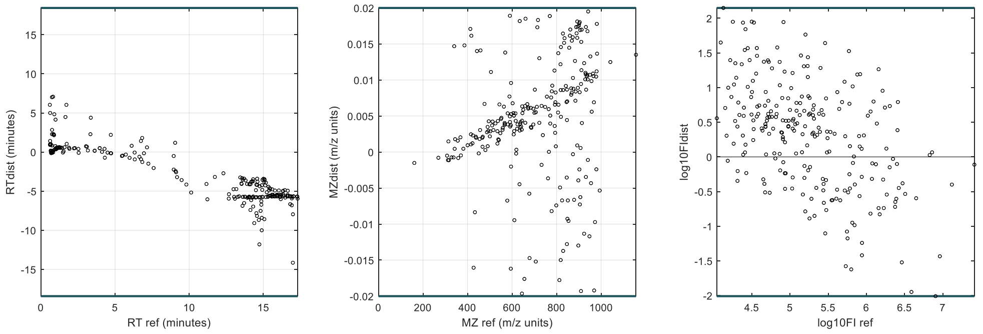

Figure SE4 4: 1-to-1 matches between the two datasets, allowing to better visualise the inter-dataset shifts.

Notice that until now the user would be just trying to figure out where the trends are, in order to select adequate threshold settings in the following phase. With the help of the plots above, at this point we could understand the main trends.

As FI is expected to be correlated in plasma samples of the two cohorts, we adjusted the target FI using the "median method". The MZ intercept and slope were manually defined by clicking the desired plot in two points at a time, using the function "M2S_calculateInterceptSlope.m":

```
[interceptSlope] = M2S_calculateInterceptSlope()
```

The settings used for the matching were then defined as below. Notice that the RT and FI thresholds are large enough to accommodate all possible matches in those dimensions, and thus the matching is entirely based on the MZ thresholds:

```
opt = struct;
opt.FIadjustMethod = 'median';
opt.multThresh.RT_intercept = [ -20 20];
opt.multThresh.RT_slope = [0 0];
opt.multThresh.MZ_intercept = [-0.0048 0.0035];
opt.multThresh.MZ_slope = [0.00000765 0.000012];
opt.multThresh.log10FI_intercept = [-10 10];
opt.multThresh.log10FI_slope = [0 0];
```

We then obtained the matches using the thresholds defined, as seen below, using the code:

```
plotType = 2;
[refSet,targetSet,Xr_connIdx,Xt_connIdx,opt]=M2S_matchAll(refFeatures,
targetFeatures,opt.multThresh,opt.FIadjustMethod,plotType);
```

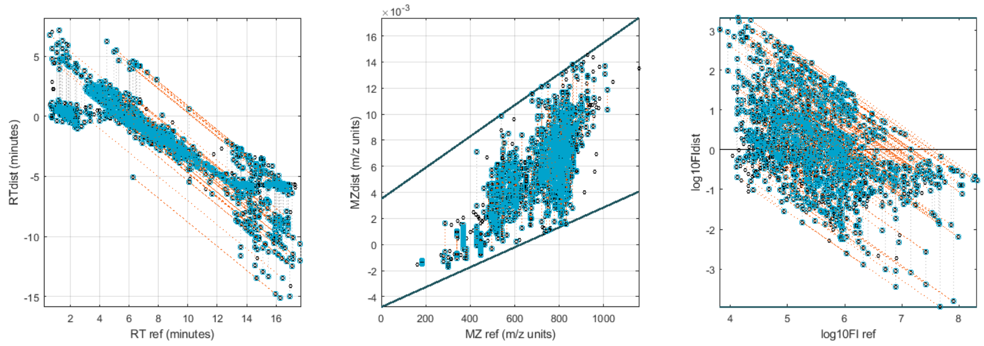

Figure SE4 5: M2S matches (black dots) plotted as distances vs reference value in the three dimensions. Matches in clusters of multiple matches (in blue) are connected by red dotted lines (multiple reference features) or blue dotted lines (multiple target features).

The inter-dataset shift trends are calculated with the definitions below.

```
opt.neighbours.nrNeighbors = 0.05;
opt.calculateResiduals.neighMethod = 'cross';
opt.pctPointsLoess = 0.1;
plotTypeResiduals = 1;
[Residuals_X,Residuals_trendline] =
M2S_calculateResiduals(refSet,targetSet,Xr_connIdx,Xt_connIdx,opt.neighbour
s.nrNeighbors,
opt.calculateResiduals.neighMethod,opt.pctPointsLoess,plotTypeResiduals)
```

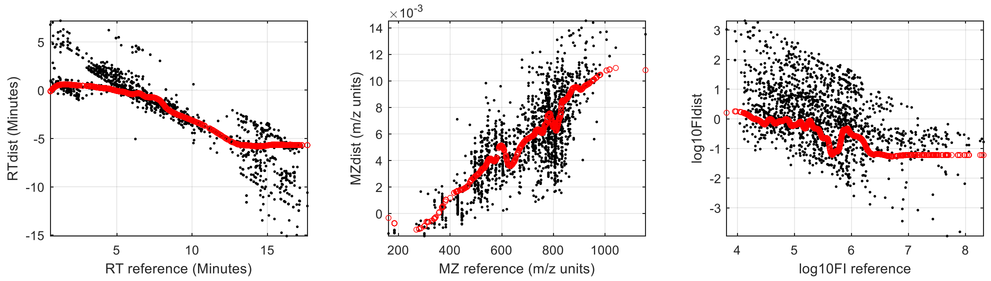

Figure SE4 6: All matches (black dots) and inter-dataset shifts for each match (red circles) in each dimension

The residuals are obtained in the same step, by subtraction of the inter-dataset shift in each dimension from each match.

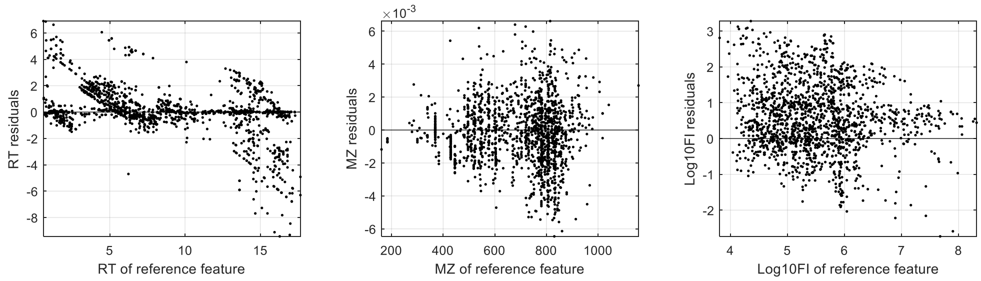

Figure SE4 7: Residuals in each dimension

The residuals are in different units (RT, MZ, log10FI). By visualising the plots above the user can harmonise them, selecting the value in each dimension that will be equal to 1 in the normalised residuals. In this case we defined them manually, and used the following settings:

```
opt.adjustResiduals.residPercentile = [1, 4e-3, 2];
[adjResiduals_X,residPercentile] = M2S_adjustResiduals(refSet,targetSet
,Residuals_X,opt.adjustResiduals.residPercentile);
```

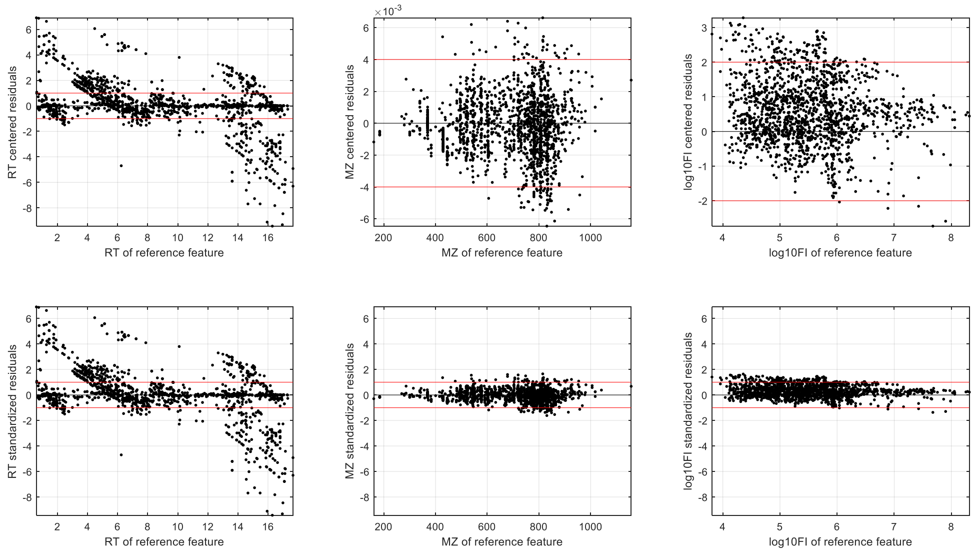

Figure SE4 8: (top) Selection of points (red lines) which become equal to 1 in the standardised residuals. (bottom) Standardised residuals obtained by dividing by the value defined for each dimension. In this case, RT is the dimension that affects the most the matches far from the inter-dataset shift.

As previously referred, plasma datasets are expected to have correlated log10FI, so we give a weight of 1 to each dimension, to build the penalisation scores. Nevertheless, by looking at the standardised residuals, the RT dimension will be the one dominating the scores:

```
opt.weights.W = [1,1,1];
plotOrNot = 1;
[penaltyScores] = M2S_defineW_getScores(refSet,targetSet, adjResiduals_X,
opt.weights.W, plotOrNot);
```

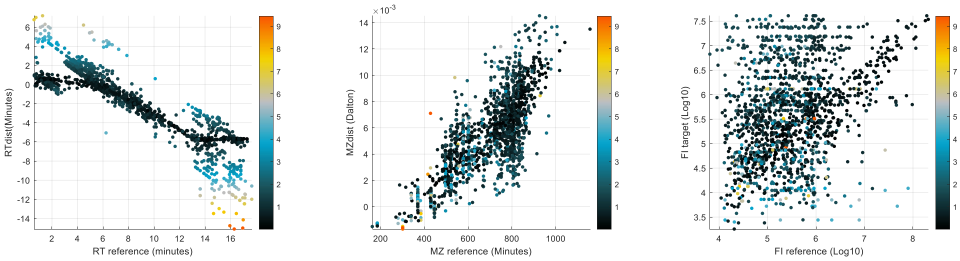

Figure SE4 9: All M2S matches coloured by penalisation scores.

The M2S algorithm successively selects the best matches from the clusters with multiple matches presented in the figure below, using the penalisation scores in the following function:

```
[eL,eL_INFO,CC_G1]= M2S_decideBestMatches(refSet, targetSet, Xr_connIdx,
Xt_connIdx, penaltyScores);
```

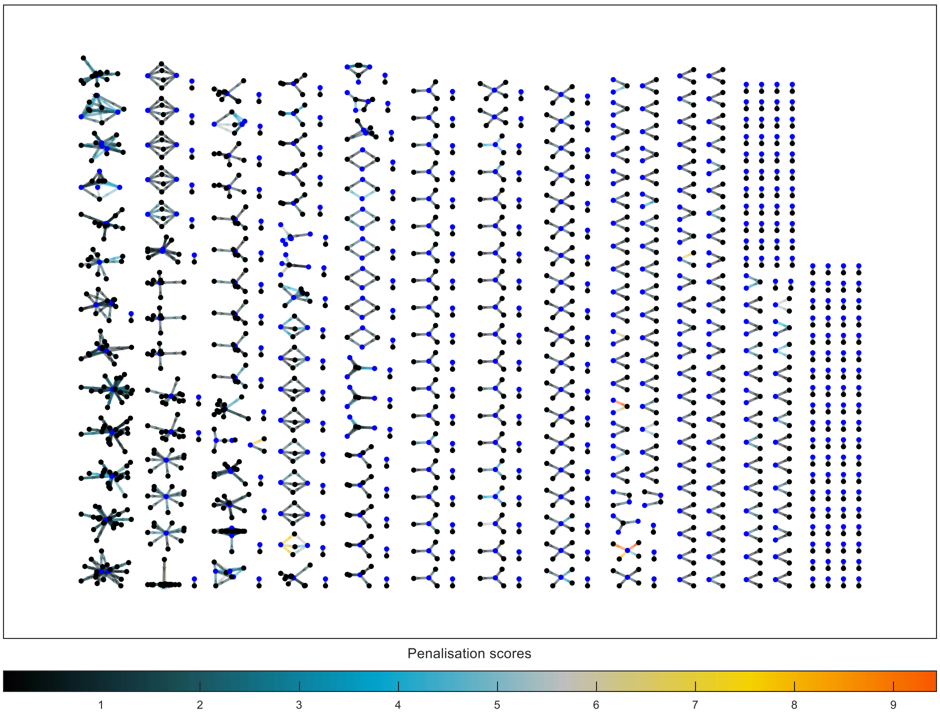

Figure SE4 10: Network with all matches (edges) coloured by penalty scores. Metabolomic features (nodes) of reference in black, target features in blue.

Finally, we adjust the thresholds for RT, MZ, log10FI, to delete possible matches that although not in clusters of multiple matches, were still found just by chance (and by using a too generous threshold definition):

```
opt.falsePos.methodType = 'trend_mad'
opt.falsePos.nrMad = 5;
plotOrNot = 1;
[eL_final, eL_final_INFO]
=M2S_findPoorMatches(eL,refSet,targetSet,opt.falsePos.methodType,opt.falseP
os.nrMad,plotOrNot)
```

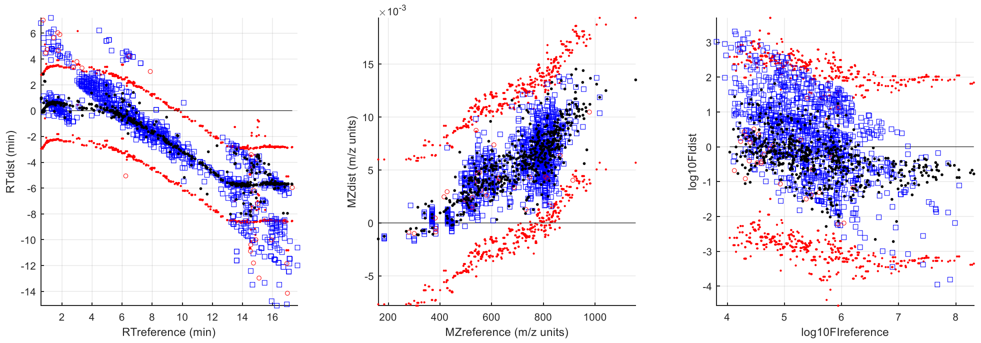

Figure SE4 11: Tightening of thresholds (red lines) used to define poor matches using the method 'trend_mad', with 5 MAD. Inter-dataset distance plots showing good matches as black dots, matches not selected from clusters as blue squares, poor matches as red circles. The limits are represented as red dots.

At this point, the matching using M2S is complete.

8.3. Comparison of results of metabCombiner and M2S

The metabCombiner software is designed for cases when the two datasets are acquired from the same biological fluid so the FI is comparable, which is the case in this example. Both methods seem competent at aligning the RT, yielding very similar results for that dimension, as we can see below.

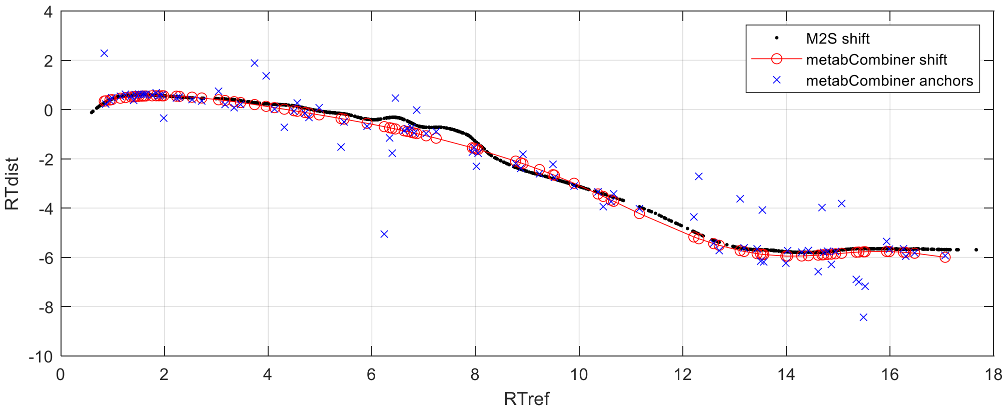

Figure SE4 12: Comparison of inter-dataset shift for the RT dimension obtained by metabCombiner and M2S. Both methods seem to perform appropriately.

As mentioned in the main text, we find some major issues with metabCombiner when compared to our method M2S. One is the limited plotting capabilities of the software, which presents a single plot during the whole process. Because of that, it was difficult to define appropriate limits for MZ thresholds, thus we missed a large set of matches outside of the defined thresholds (blue circle in the figures below). Another is the lack of alignment of MZ, which may show a systematic shift in this case. As in this example there is a systematic difference in MZ at higher values of MZ, the lack of alignment of MZ had a negative impact. In clusters, the selected match was the one closest to MZ=0 (red circle in figures below) and not the one close to the inter-dataset MZ shift trend, as we think is appropriate and is used in M2S.

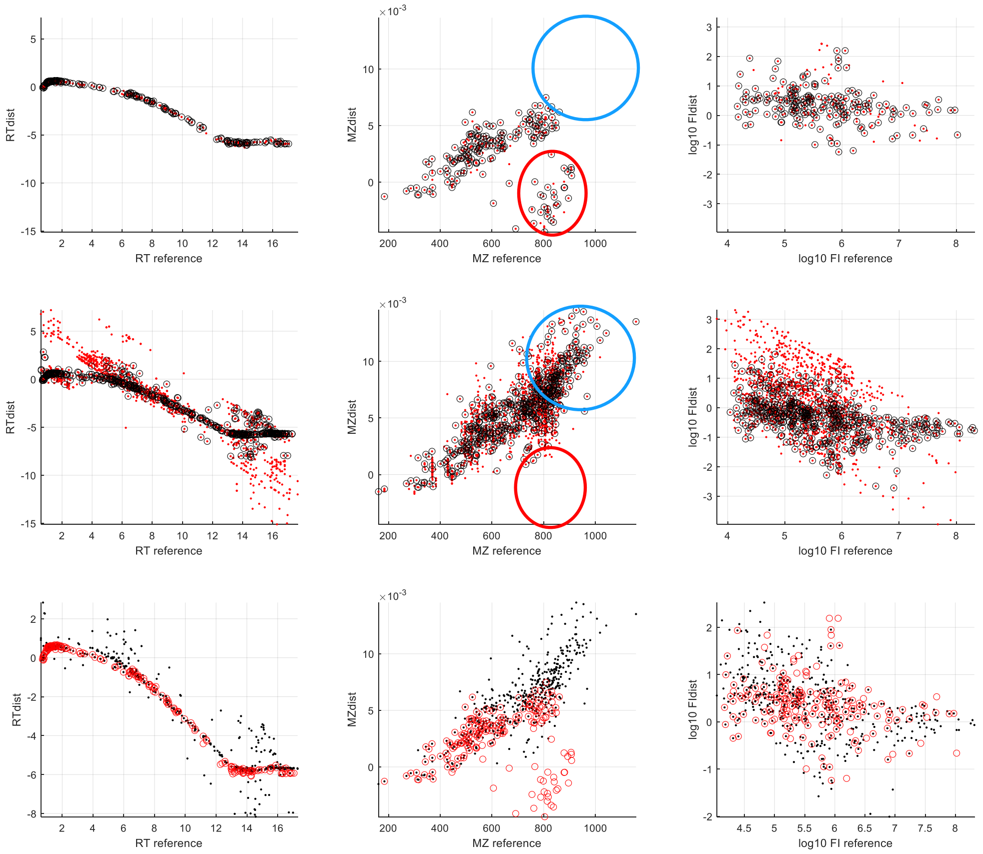

Figure SE4 13: Results using (top) metabCombiner, (middle) M2S, and (bottom) 1-to-1 matches comparison between M2S and metabCombiner. In the top and middle plots the red dots are multiple matches and the black circles are final 1-to-1 matches. In the bottom plot the black dots are M2S and the red circles are metabCombiner final 1-to-1 matches. The blue circles indicate matches that seem to not have been found in metabCombiner due to defective choice of thresholds. The red circles indicate areas with metabCombiner matches that seem to have been wrongly decided from clusters of multiple matches.

The final number of matches using both methods is presented in the table below.

Table SE4 1: Number of features and matches found in the different phases of the matching.

Match type	M2S	metabCombiner
Features to match (ref/target)	3633 / 1822	
Clusters of matches	1632	247
1-to1 matches	522	195
Common matches	137	
Different matches	385	58
With different ref OR target	43	38
With different ref AND target	342	20

These matches were not annotated in the two datasets, so it is not possible to count a number of correctly matched annotated features. From an initial number of 3633 reference and 1822 target features, M2S found 1632 clusters which yielded 522 unique matches, while metabCombiner found 247 clusters, yielding 195 matches. From these, 137 matches were exactly the same in M2S and metabCombiner. M2S found 385 matches that were not found in metabCombiner, 43 of them containing a reference or target feature that was matched to something else in metabCombiner, while 342 contained both reference and target features that were never matched by metabCombiner. Similarly, metabCombiner found 58 (38 + 20) matches that were not reciprocated in M2S.

The large difference in the final "1-to-1 matches" (522/195) reflects the difficulty of setting the right parameters in metabCombiner because of the lack of visualisations. We think that with the right thresholds metabCombiner numbers might approach M2S numbers. On the contrary, the relatively small number of "common matches" (137 in the possible 195) is due to the different choice of "best" matches in the clusters with multiple matches and cannot be rectified. As metabCombiner does not correct the MZ inter-dataset shift, it tends to select matches that have lower MZ difference between datasets, rather than lower MZ difference to the systematic shift. We think that M2S was superior on this matching of the two datasets.

9. Example S5: Airwave plasma HPOS vs MESA serum HPOS

This example has three objectives:

- Show the applicability of the M2S method to **non-lipidomics datasets with large retention time differences**

- Compare the M2S methodology and results with another method, metabCombiner, **without using annotations** to assist in the matching

- Add practical information about the use of some functions and strategy for matching using M2S

These are two large datasets, obtained using **similar – though not identical – analytical methods**, and from **slightly different biological fluids**, plasma in Airwave and serum in MESA1. Hydrophilic interaction (liquid) chromatography was used in both cases (with ESI positive mode), using **different elution gradients,** resulting in chromatograms with a **significant retention time differences** (up to 1.65 minutes in a total of 6 minutes), as well as **significant m/z systematic difference**. The peaks in each sample were detected, integrated and assembled into a single table using **different software packages**, XCMS for Airwave and Progenesis QI for MESA1. Please notice that Progenesis QI aggregates features of the same metabolite and selects one of the features to represent the metabolite in the dataset. This feature may not be the same in both datasets, reducing the number of matches. The Airwave 1 dataset was acquired by the National Phenome Centre at Imperial College London, while MESA1 was acquired by Metabometrix, a metabolomics services company at Imperial College London. For this article the median values RT, MZ, FI were then obtained/calculated for each of the datasets, and the datasets were matched.

9.1. Matching using metabCombiner

We tried to replicate the experience of a regular user, accessing both the article and an online tutorial for information. For the R session we followed the online tutorial posted at [12], and the function calls and discussion in this section can be better understood by following that webpage.

We used the default 'binGapValue' of 0.005 m/z, to combine the features into groups, obtaining 568 groups of features. Then we used the settings below to define the anchors. Anchors are the inter-dataset-matched highest intensity features in each dataset within retention time and m/z delimited windows. This methodology forces the feature intensity to be comparable in both sets, thus should only work in datasets of the same biological fluids.

p.combined.2 = selectAnchors(p.combined, windx = 0.03, windy = 0.02, tolQ = 0.3, tolmz = 0.005, useID = FALSE)

We modelled the inter-dataset retention time shift using the Generalized Additive Model with the following settings:

set.seed(100)

p.combined.3 = fit_gam(p.combined.2, useID = FALSE, k = seq(12,20,2), iterFilter = 2, coef = 2, prop = 0.5, bs = "bs", family = "gaussian", m = c(3,2))

The only plot supplied by the metabCombiner package is the following:

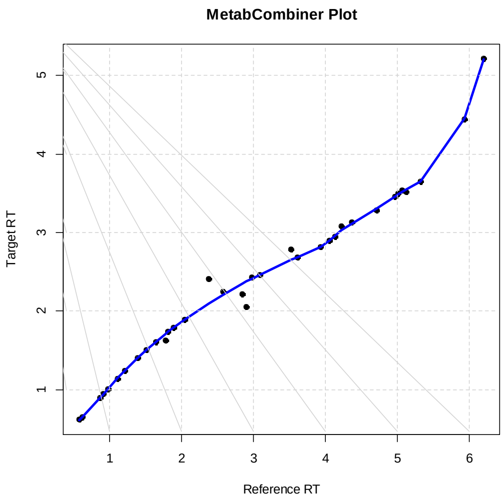

Figure SE5 1: metabCombiner scatter plot of RT of target vs reference showing the differences between both. The black dots are the anchor matches, and the blue line is the modelled inter-dataset shift.

We then calculated the scores by adjusting the weights as:

p.combined.4 = calcScores(p.combined.3, A = 85, B = 15, C = 0.5, usePPM = FALSE, useAdduct = FALSE, groups = NULL)

The results of the matching were collected in two ways, one containing a table with all matches (including conflicts), and another forcing a decision on the conflicts, yielding only one-to-one matches.

The function calls to obtain all matches were:

combined.table.byMZRT = labelRows(combined.table, minScore = 0.5, maxRankX = 3, maxRankY = 3, method = "mzrt", balanced = TRUE, delta = c(0.005,0.5,0.005,0.5))

The instructions for obtaining only one-to-one matches were:

combined.table.finalReport = reduceTable(combined.table, minScore = 0.5, maxRankX = 3, maxRankY = 3).

After these steps the matching the matching of the two datasets using metabCombiner was complete.

9.2. Matching using M2S

We loaded the data and created unique MZRT string identifiers representing each of the features.

```
[refFeatures] = importdata(refFilename);
```

```
[targetFeatures] = importdata(targetFilename);

[refMZRT_str] =
M2S_createLabelMZRT('ref',refFeatures(:,2),refFeatures(:,1));
[targetMZRT_str] =
M2S_createLabelMZRT('target',targetFeatures(:,2),targetFeatures(:,1));
```

The two datasets are presented in the figures below.

```
M2S_figureH(0.8,0.5)
subplot(1,2,1),
M2S_plotMZRT_featureSet(refFeatures,1,8,1);
subplot(1,2,2),
M2S_plotMZRT_featureSet(targetFeatures,1,8,1);
```

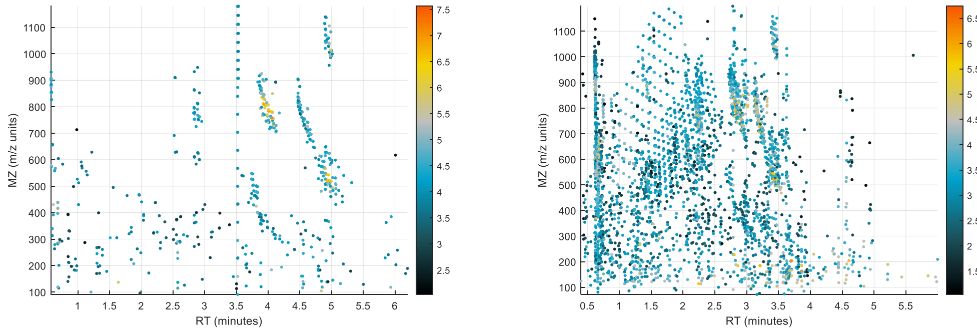

Figure SE5 2: MZ vs RT plots of plasma positive mode LC-MS HILIC of experiments Airwave plasma (left) and MESA1 serum (right), coloured by log10FI

After setting only large MZ thresholds (maximum difference of 0.02 m/z between cohorts) it is possible to see the trends representing the shifts between the datasets. For these thresholds, one can use the function M2S_matchAll with default settings as below.

```
[refSet_i,targetSet_i,Xr_connIdx_i,Xt_connIdx_i,opt_i]=M2S_matchAll(
refFeatures, targetFeatures)
```

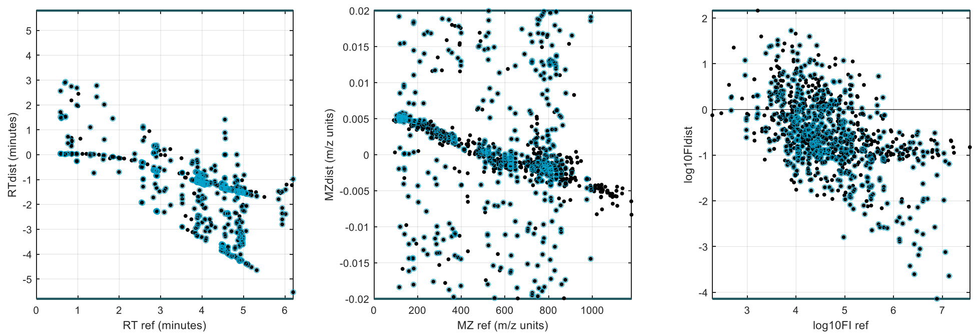

Figure SE5 3: Plot of all matches within thresholds in all three dimensions after using function “M2S_matchAll.m” with default settings. Black dots are 1-to-1 matches, and blue dots are matches that are part of clusters with multiple matches. The major inter-dataset shifts are visible.

We then applied the following settings to match the two datasets:

```
opt = struct;
opt.FIadjustMethod = 'median';
opt.multThresh.RT_intercept = [ -2.1,1];
opt.multThresh.RT_slope = [0 0];
opt.multThresh.MZ_intercept = [-0.003919186017602 0.013418570345910];
```

```
opt.multThresh.MZ_slope = [-0.000005141022420 -0.000004477501300];
opt.multThresh.log10FI_intercept = [-1000 1000];
opt.multThresh.log10FI_slope = [0 0];
```

The MZ intercept and slope were defined using the function “M2S_calculateInterceptSlope.m”, which facilitates the choice of these parameters by clicking directly on the plot of choice.

```
[interceptSlope] = M2S_calculateInterceptSlope()
```

The shifts are now perfectly visible in the RT and MZ. Notice how the MZ shift is not close to zero at lower MZ values. Also, notice the larger difference in log10FI for features with low values.

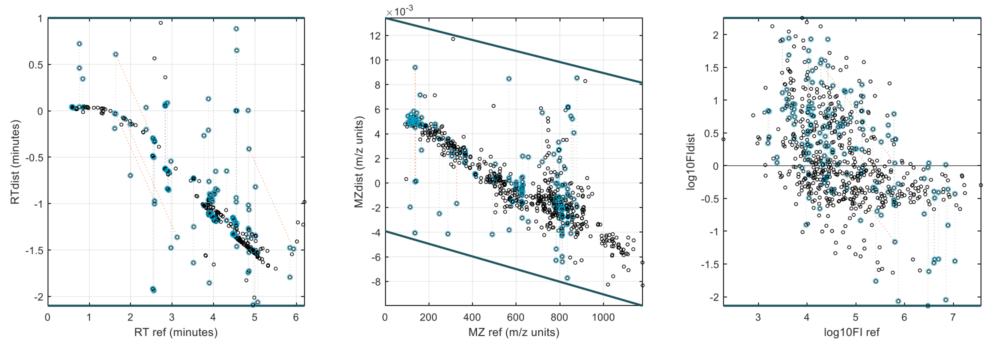

Figure SE5 4: Plot of all matches within thresholds in all three dimensions after using function “M2S_matchAll.m” with final settings. Black dots are 1-to-1 matches, and blue dots are matches that are part of clusters with multiple matches. Multiple matches of the same cluster are connected by dotted lines.

The inter-dataset shifts are modelled using the settings below. Notice the adaptation of the inter-dataset shift to the matching points, arguably over-fitting, which could be changed by increasing the percentage of points in the loess curve.

```
opt.neighbours.nrNeighbors = 0.025;
opt.calculateResiduals.neighMethod = 'cross';
opt.pctPointsLoess = 0.1;
plotTypeResiduals = 1
[Residuals_X,Residuals_trendline] = M2S_calculateResiduals(refSet,
targetSet, Xr_connIdx,Xt_connIdx,opt.neighbours.nrNeighbors,
opt.calculateResiduals.neighMethod, opt.pctPointsLoess, plotTypeResiduals)
```

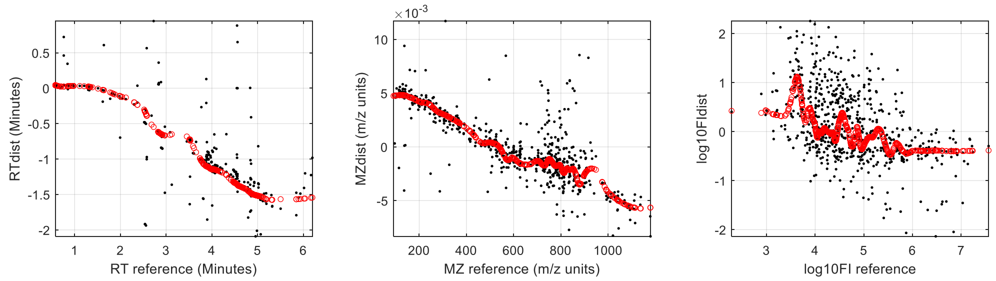

Figure SE5 5: All matches (black dots) and inter-dataset shifts for each match (red circles) in each dimension

The residuals are obtained in the same step, by subtraction of the inter-dataset shift in each dimension from each match.

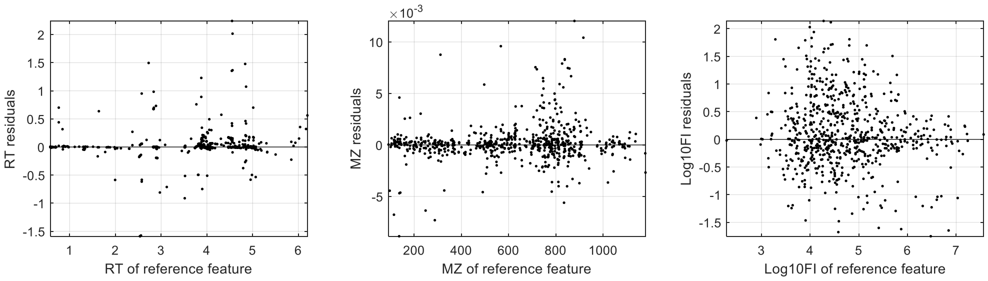

Figure SE5 6: Residuals in each dimension

The residuals are in different units (RT, MZ, log10FI). By visualising the plots above the user can harmonise them, selecting the value in each dimension that will be equal to 1 in the normalised residuals. We used the default settings, meaning in each dimension the median of the residuals plus 3 times the median absolute deviation (MAD) will be equal to 1 (red lines), as below:

```
[adjResiduals_X,residPercentile] = M2S_adjustResiduals (refSet, targetSet,
Residuals_X,NaN);
```

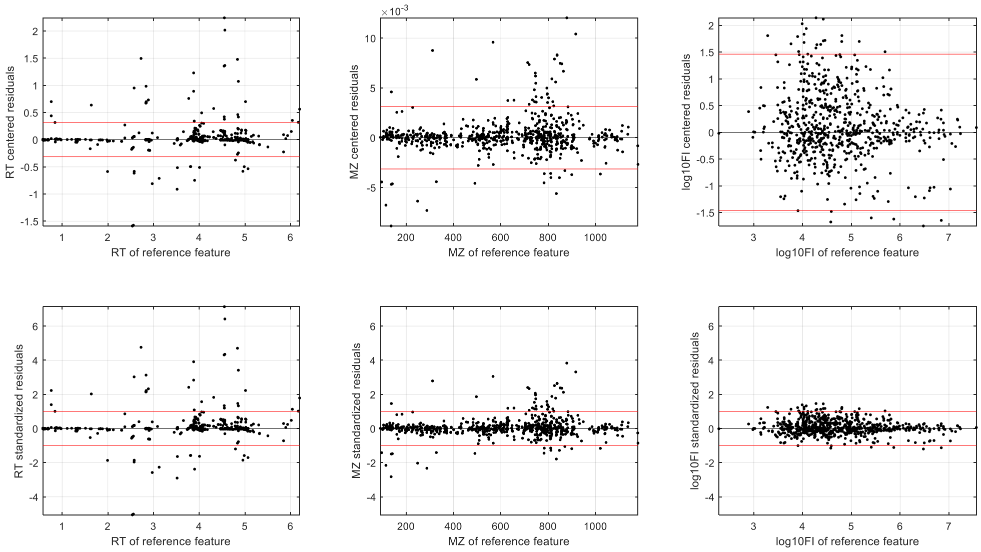

Figure SE5 7: (top) Selection of points (red lines) which become equal to 1 in the standardised residuals. (bottom) Standardised residuals obtained by dividing by the value defined for each dimension. In this case, both RT and MZ are the dimensions that affect the most the matches far from the inter-dataset shift.

As one of the datasets was plasma and the other was serum we decided to not use FI to build the scores. Although we can see (in the plot on the right in the figure below) that log10FI are correlated, there can be some metabolomic features for which that correlation may not apply. We thus use the default settings for the weights (W = [1, 1, 0]), to build the penalisation scores:

```
[penaltyScores] = M2S_defineW_getScores(refSet,targetSet,adjResiduals_X);
```

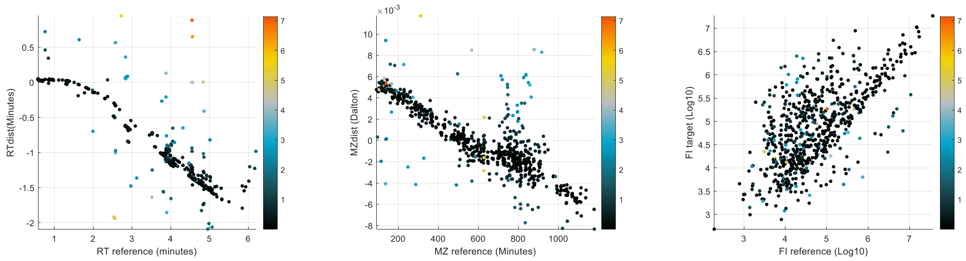

Figure SE5 8: All M2S matches coloured by penalisation scores.

The M2S algorithm successively selects the best matches from the clusters with multiple matches presented in the figure below, using the penalisation scores in the following function:

```
[eL,eL_INFO,CC_G1]= M2S_decideBestMatches(refSet, targetSet, Xr_connIdx,
Xt_connIdx, penaltyScores);
```

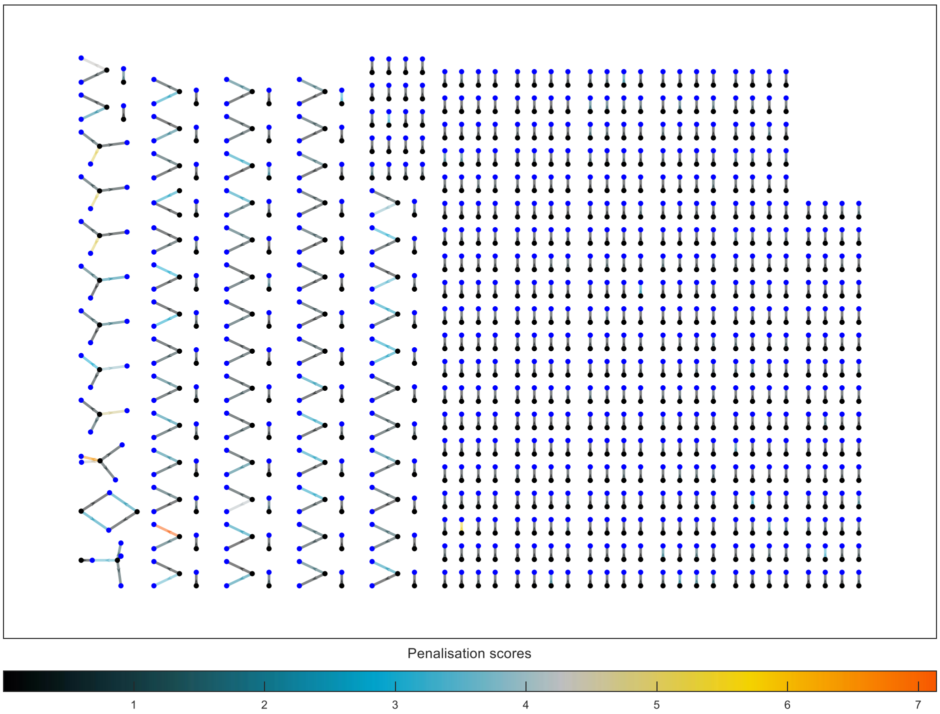

Figure SE5 9: Network with all matches (edges) coloured by penalty scores. Metabolomic features (nodes) of reference in black, target features in blue.

Finally, we adjust the thresholds or RT, MZ, log10FI, to delete possible matches that although not in clusters of multiple matches, were still found just by chance (and too generous threshold definition). In this case we used default settings, (meaning `methodType='residuals_mad'; nrMad=5; plotOrNot = 1;`)

```
[eL_final, eL_final_INFO] = M2S_findPoorMatches(eL,refSet,targetSet);
```

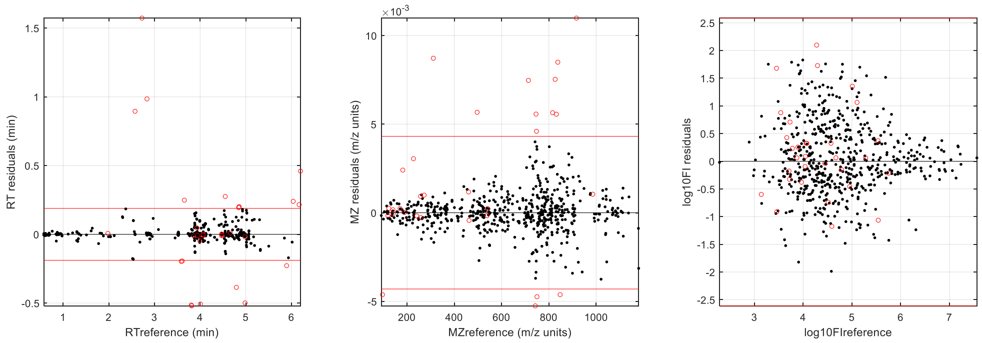

Figure SE5 10: Tightening of thresholds (red lines) used to define poor matches using the method "residuals_mad", with 5 MAD. Residuals plots showing good matches as black dots, poor matches as red circles. The limits are represented by red lines.

At this point, the matching using M2S is complete.

9.3. Comparison of results of metabCombiner and M2S

The metabCombiner software is designed for cases when the two datasets are acquired from the same biological fluid so the FI is comparable. In this case we are matching plasma and serum and both methods seem competent at aligning the RT, yielding very similar results for that dimension, as we can see below.

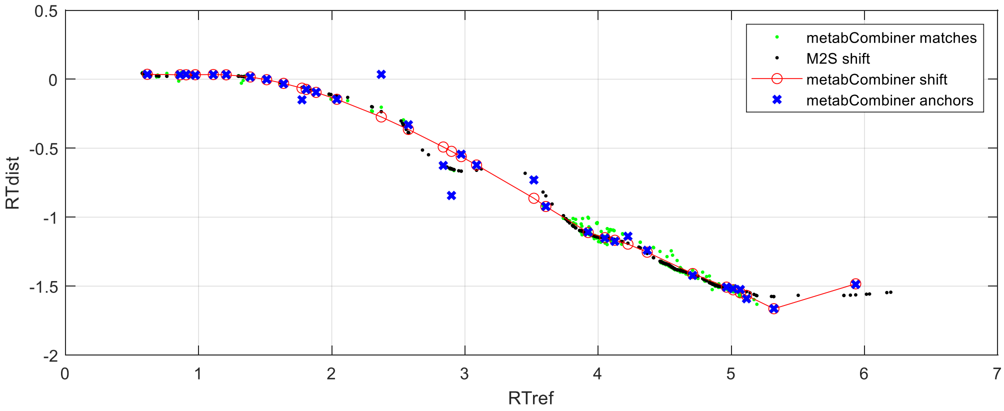

Figure SE5 11: Comparison of inter-dataset shift for the RT dimension obtained by metabCombiner and M2S. Both methods seem to perform appropriately.

As in the previous case, the lack of visualisation in the metabCombiner method made it difficult to define appropriate limits for MZ thresholds, thus by using the specified thresholds metabCombiner missed a number of matches outside of those (blue circle in the figure below). Another issue is the lack of alignment of MZ (and in some cases FI), which may show a systematic effect that needs modelling. As in this case there was a relevant systematic trend in MZ at low values of MZ, the lack of alignment of MZ had a negative impact on the choice of the best of the multiple matches. Fortunately, there were not many multiple matches at low MZ, thus it seems to only have affected two matches that happened to be closer to MZ=0 (red circle in figure below). In this case M2S selected the ones closer to the inter-dataset shift instead.

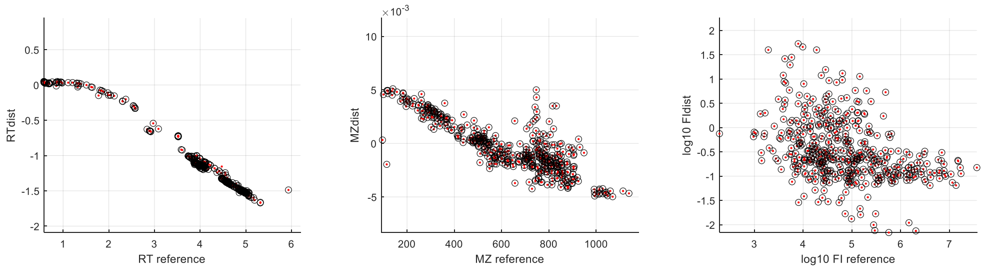

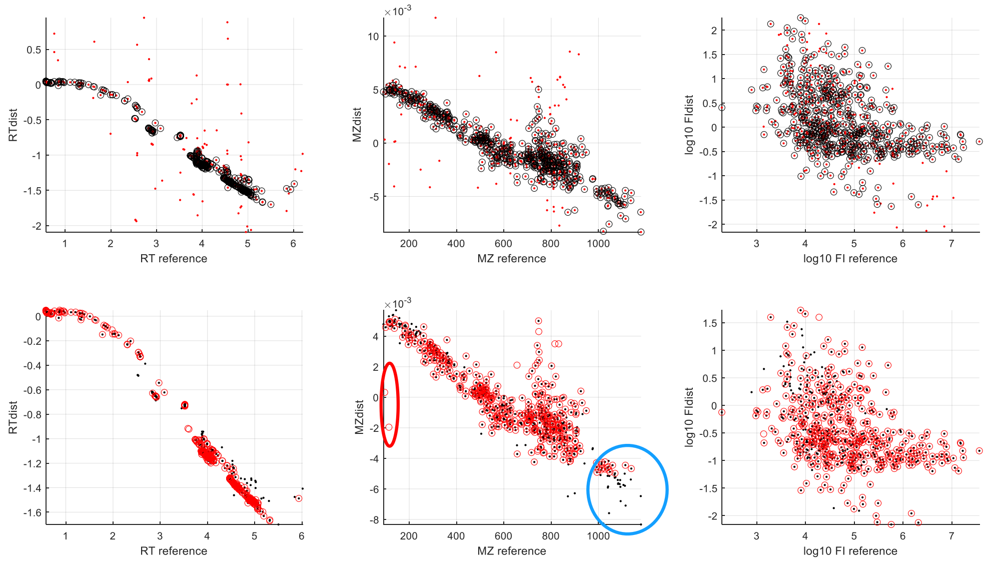

Figure SE5 12: Results using (top) metabCombiner, (middle) M2S, and (bottom) 1-to-1 matches comparison between M2S and metabCombiner. In the top and middle plots the red dots are multiple matches and the black circles are final 1-to-1 matches. In the bottom plot the black dots are M2S and the red circles are metabCombiner final 1-to-1 matches. The blue circles indicate matches that seem to not have been found in metabCombiner due to defective choice of thresholds. The red circles indicate areas with metabCombiner matches that seem to have been wrongly decided from clusters of multiple matches.

Table SE5 1: Number of features and matches found in the different phases of the matching.

Match type	M2S	metabCombiner
Features to match (ref/target)	784/5093	
Clusters of matches	680	461
1-to1 matches	571	460
Common matches	450	
Different matches	121	10
With different ref OR target	3	3
With different ref AND target	118	7

These matches were not annotated in the two datasets, so it is not possible to count a number of correctly matched annotated features. From an initial number of 784 reference and 5093 target features, M2S found 680 clusters which yielded 571 unique matches, while metabCombiner found 461 clusters, yielding 460 matches. From these, 450 matches were exactly the same in M2S and metabCombiner. M2S found 121 matches that were not found in metabCombiner, 3 of them containing a reference or target feature that was matched to something else in metabCombiner, while 118 contained both reference and target features that were never matched by metabCombiner. Similarly, metabCombiner found 10 (3 + 7) matches that were not reciprocated in M2S.

The difference in the final “1-to-1 matches” (571/460) reflects the fact that it was not easy to select the right parameters in metabCombiner because of the lack of visualisations. We think that with the right thresholds metabCombiner numbers would approach M2S numbers. As mentioned above, the lack of MZ modelling by metabCombiner didn’t have a strong detrimental effect only because of the small number of clusters of multiple matches. We think that M2S was superior on this matching of the two datasets.

10.Example S6: MESA serum HPOS vs Airwave urine HPOS

This example has three objectives:

- Show the applicability of the M2S method to datasets of **different biological fluids**

- Compare the M2S methodology and results with another method, metabCombiner, **without using annotations** to assist in the matching

- Add practical information about the use of some functions and strategy for matching using M2S

The two large datasets were acquired from **different biological fluids**, serum in MESA2 and urine in Airwave. Hydrophilic interaction (liquid) chromatography (HILIC) was used in both, with mass spectrometry detection using ESI in the positive ionisation mode, resulting in chromatograms with a small retention time difference (up to 0.25 minutes in a total of 6 minutes). The peaks in each sample were detected, integrated and assembled into a single table using **different software packages**, XCMS for MESA and Progenesis QI for Airwave. Note that Progenesis QI aggregates features detected for the same metabolite and selects one of them to represent the metabolite in the dataset. The selected feature may not be the same in both datasets, reducing the number of matches. For this article the median values RT, MZ, FI were then obtained/calculated for each of the datasets, and the datasets were matched.

10.1. Matching using metabCombiner

We tried to replicate the experience of a regular user, accessing both the article and an online tutorial for information. For the R session we followed the online tutorial posted at [12], and the function calls and discussion in this section can be better understood by following that webpage.

We used the 'binGapValue' of 0.01 m/z, to combine the features into groups, obtaining 1045 groups of features. Then we used the settings below to define the anchors. Anchors are the inter-dataset-matched highest intensity features in each dataset within retention time and m/z delimited windows. This methodology forces the feature intensity to be comparable in both sets, thus should only work in datasets of the same biological fluids.

p.combined.2 = selectAnchors(p.combined, windx = 0.02, windy = 0.03, tolQ = 1.1, tolmz = 0.01, useID = FALSE)

We modelled the inter-dataset retention time shift using a Generalized Additive Model with the following settings:

set.seed(100)

p.combined.3 = fit_gam(p.combined.2, useID = FALSE, k = seq(12,20,2), iterFilter = 2, coef = 2, prop = 0.5, bs = "bs", family = "gaussian", m = c(3,2))

The only plot supplied by the metabCombiner package is the following:

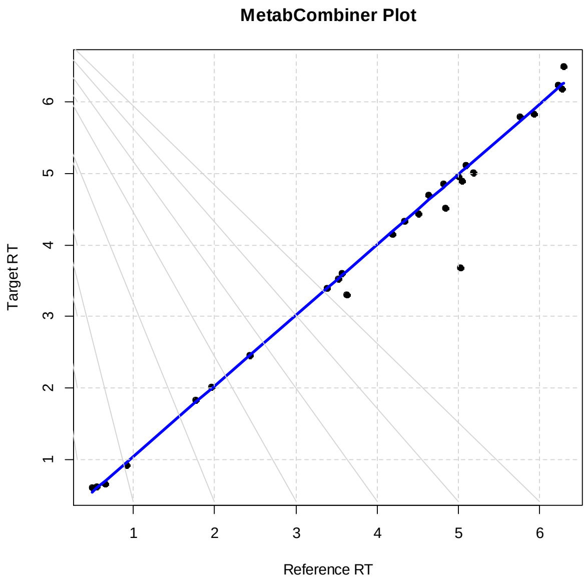

Figure SE6 1: metabCombiner scatter plot of RT of target vs reference showing the differences between both. The black dots are the anchor matches, and the blue line is the modelled inter-dataset shift.

We then calculated the scores by adjusting the weights as:

p.combined.4 = calcScores(p.combined.3, A = 70, B = 7, C = 0, usePPM = FALSE, useAdduct = FALSE, groups = NULL)

The final results of the matching were collected in two ways, one containing a table with all matches (including conflicts), and another forcing a decision on the conflicts, yielding only one-to-one matches.

The function calls to obtain all matches were:

combined.table.byMZRT = labelRows(combined.table, minScore = 0.5, maxRankX = 3, maxRankY = 3, method = "mzrt", balanced = TRUE, delta = c(0.005,0.5,0.005,0.5))

The instructions for obtaining only one-to-one matches were:

combined.table.finalReport = reduceTable(combined.table, minScore = 0.5, maxRankX = 3, maxRankY = 3)

After these steps the matching of the two datasets using metabCombiner was complete.

10.2. Matching using M2S

We loaded the data and created unique MZRT string identifiers representing each of the features.

```
[refFeatures] = importdata(refFilename);
[targetFeatures] = importdata(targetFilename);

[refMZRT_str] =
M2S_createLabelMZRT('ref',refFeatures(:,2),refFeatures(:,1));
[targetMZRT_str] =
M2S_createLabelMZRT('target',targetFeatures(:,2),targetFeatures(:,1));
```

The two datasets are presented in the figures below. As expected, the datasets look very different from each other, mostly because of a lack of features at *m/z* values higher than 500.

```
M2S_figureH(0.8,0.5)
subplot(1,2,1),
M2S_plotMZRT_featureSet(refFeatures,1,8,1);
subplot(1,2,2),
M2S_plotMZRT_featureSet(targetFeatures,1,8,1);
```

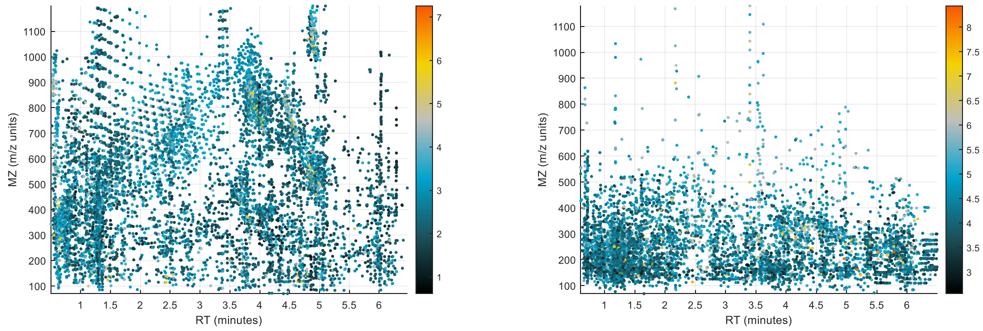

Figure SE6 2: MZ vs RT plots of positive mode LC-MS HILIC of experiments MESA serum (right), and Airwave urine (right), coloured by log10FI

After setting only MZ thresholds (0.02), although there is a massive number of matches, it is possible to see the trends representing the shifts between the datasets (using function "M2S_plotDelta_matchedSets.m"), especially in the RT dimension.

The code used was:

```
[refSet_i,targetSet_i,Xr_connIdx_i,Xt_connIdx_i,opt_i]=
M2S_matchAll(refFeatures,targetFeatures)
M2S_figureH(0.8,0.8)
M2S_plotDelta_matchedSets(refSet_i,targetSet_i,'.k')
```

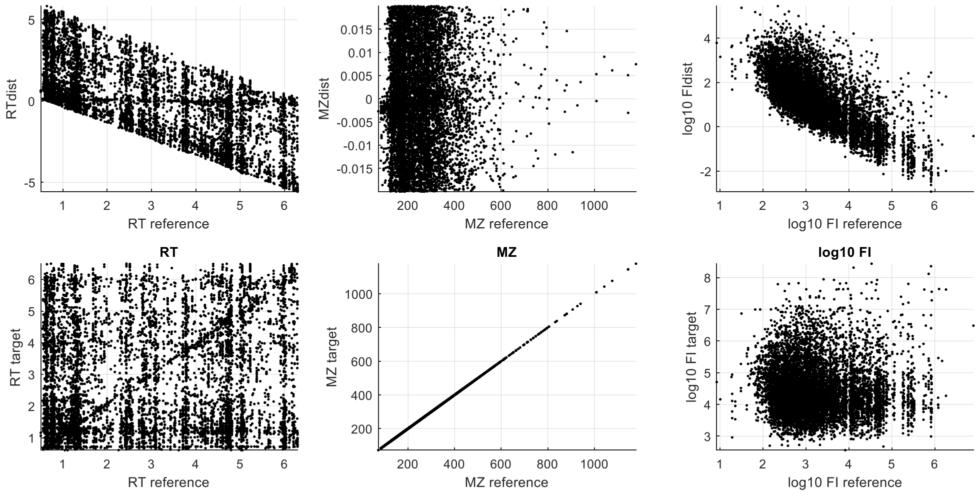

Figure SE6 3: Plot of matches using function "M2S_plotDelta_matchedSets.m". These functions help at visualising the inter-dataset shifts in more complex cases. Top row: differences vs reference values for each of the dimensions. Bottom row: target vs reference values for each of the dimensions.

We do not expect the FI to be highly correlated in these two different biofluids, so we do not adjust the FI at all. After experimenting with several threshold settings, these were defined as:

```
opt = struct;
opt.FIadjustMethod = 'none';
```

```
opt.multThresh.RT_intercept = [-0.167660910518054   0.247270821283979];
opt.multThresh.RT_slope = [-0.016180962050604 -0.013785753852324];
opt.multThresh.MZ_intercept = [-0.006 0.001231968031968  ];
opt.multThresh.MZ_slope = [0  0.000008097815277];
opt.multThresh.log10FI_intercept = [-10 10];
opt.multThresh.log10FI_slope = [0 0];
```

The MZ intercept and slope were defined using the function “M2S_calculateInterceptSlope.m”, which facilitates the choice of these parameters by clicking directly on the plot of choice.

```
[interceptSlope] = M2S_calculateInterceptSlope()
```

The matches found within thresholds are shown below.

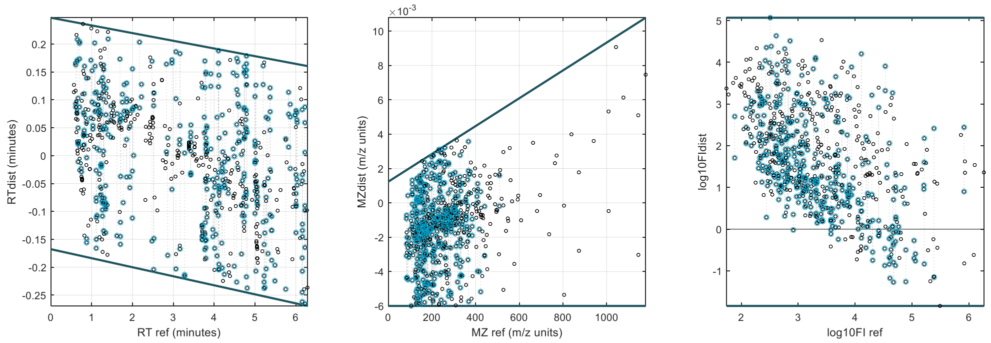

Figure SE6 4: Plot of all matches within thresholds in all three dimensions after using function “M2S_matchAll.m” with final settings. Black dots are 1-to-1 matches, and blue dots are matches that are part of clusters with multiple matches. Multiple matches of the same cluster are connected by dotted lines.

The inter-dataset shifts were modelled using the following settings:

```
opt.neighbours.nrNeighbors = 11;
opt.pctPointsLoess = 0.25;
opt.calculateResiduals.neighMethod = 'circle';
plotTypeResiduals = 1;
[Residuals_X,Residuals_trendline] =
M2S_calculateResiduals(refSet,targetSet, Xr_connIdx,Xt_connIdx,
opt.neighbours.nrNeighbors,  opt.calculateResiduals.neighMethod,
opt.pctPointsLoess, plotTypeResiduals)
```

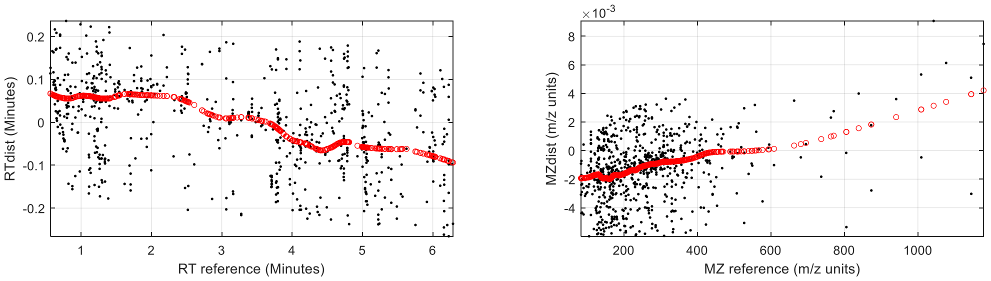

Figure SE6 5: All matches (black dots) and inter-dataset shifts for each match (red circles) in each dimension

The residuals are obtained in the same step, by subtraction of the inter-dataset shift in each dimension from each match.

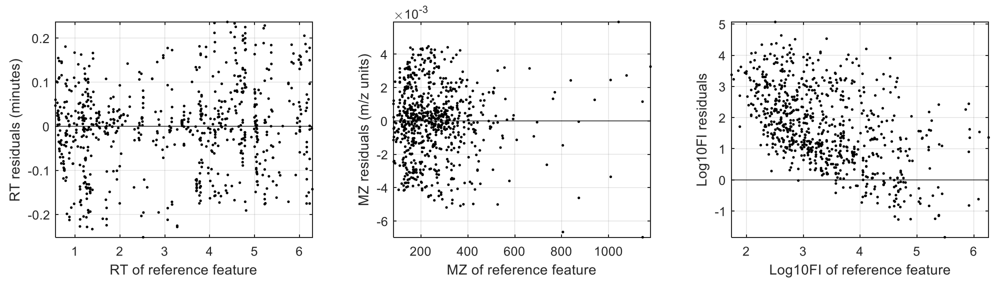

Figure SE6 6: Residuals in each dimension

The residuals are in different units (RT, MZ, log10FI). By visualising the plots above the user can harmonise them, selecting the value in each dimension that will be equal to 1 in the normalised residuals. We used the settings below, being careless about FI (we randomly set a value of 5), as later we will set the FI weight to zero.

```
opt.adjustResiduals.residPercentile = [0.1,0.002,5];
[adjResiduals_X,residPercentile] = M2S_adjustResiduals(refSet, targetSet,
Residuals_X,opt.adjustResiduals.residPercentile);
```

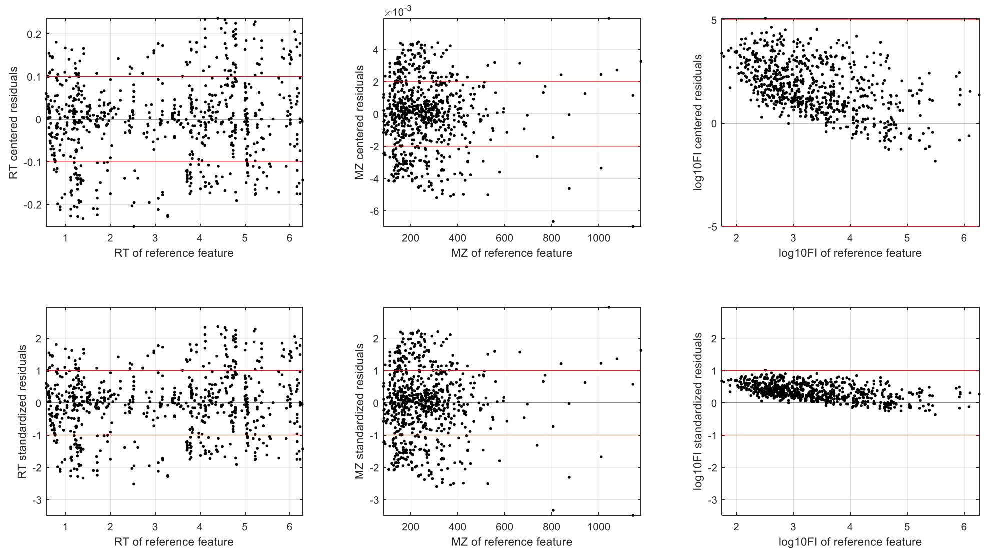

Figure SE6 7: (top) Selection of points (red lines) which become equal to 1 in the standardised residuals. (bottom) Standardised residuals obtained by dividing by the value defined for each dimension. In this case, both RT and MZ are the dimensions that affect the most the matches far from the inter-dataset shift.

As one of the datasets was serum and the other was urine we preferred to not use FI at all in the scores. We thus use the default settings for the weights (W = [1, 1, 0]), to build the penalisation scores:

```
W = [1,1,0]
[penaltyScores] = M2S_defineW_getScores(refSet, targetSet, adjResiduals_X,
W,1);
```

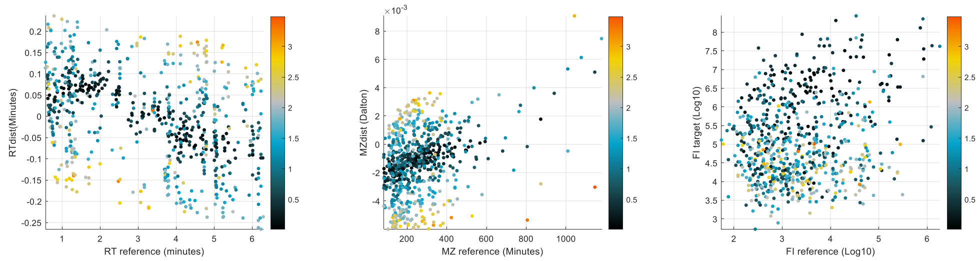

Figure SE6 8: All M2S matches coloured by penalisation scores.

The M2S algorithm successively selects the best matches from the clusters with multiple matches presented in the figure below, using the penalisation scores in the following function:

```
[eL,eL_INFO,CC_G1]= M2S_decideBestMatches(refSet, targetSet, Xr_connIdx,
Xt_connIdx, penaltyScores);
```

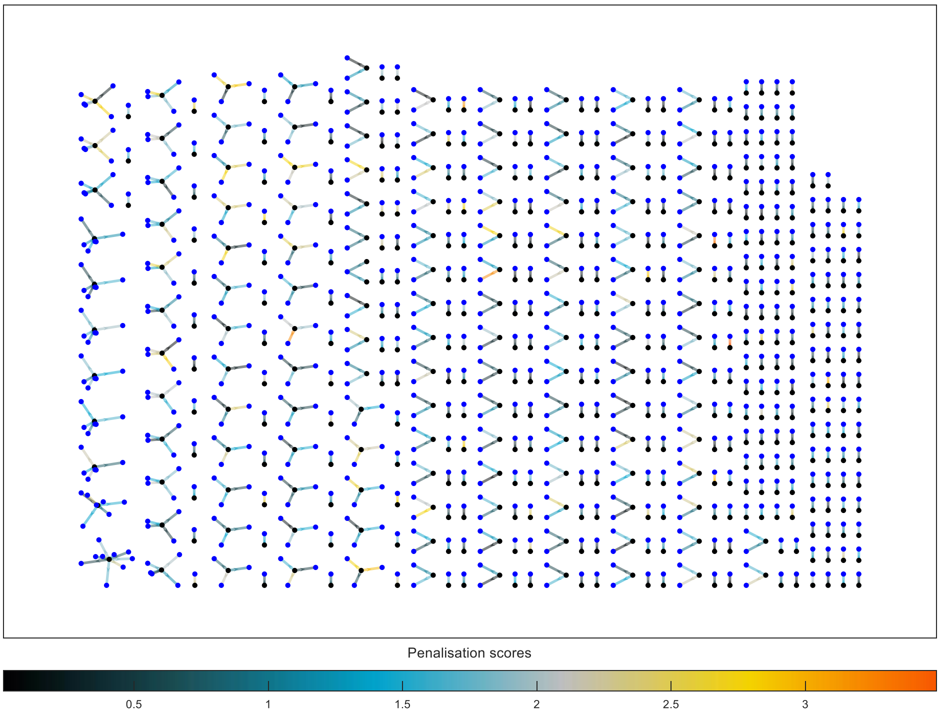

Figure SE6 9: Network with all matches (edges) coloured by penalty scores. Metabolomic features (nodes) of reference in black, target features in blue.

Finally, we adjust the thresholds or RT, MZ, log10FI, to delete possible matches that although not in clusters of multiple matches, were still found just by chance (and too generous threshold definition). In this case we used the "residuals_mad", keeping all matches with residuals within 3 MAD:

```
opt.falsePos.methodType = 'residuals_mad';
opt.falsePos.nrMad = 3;
plotOrNot = 1;
[eL_final, eL_final_INFO] =M2S_findPoorMatches(eL,refSet, targetSet,
opt.falsePos.methodType, opt.falsePos.nrMad, plotOrNot);
```

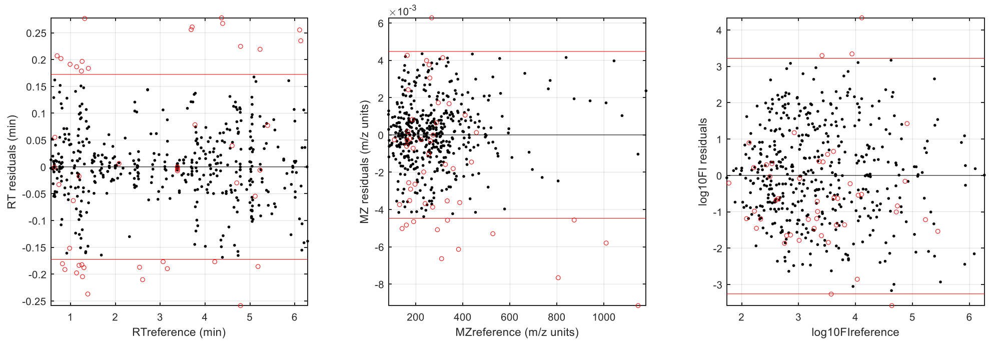

Figure SE6 10: Tightening of thresholds (red lines) used to define poor matches using the method "residuals_mad", with 3 MAD. Residuals plots showing good matches as black dots, poor matches as red circles. The limits are represented by red lines.

At this point, the matching using M2S is complete.

10.3. Comparison of results of metabCombiner and M2S

The metabCombiner software is designed for cases when the two datasets are acquired from the same biological fluid, so FI is comparable. On the other hand, for datasets from different biological samples with uncorrelated FI we expect metabCombiner to fail **if there are large RT shifts and many multiple matches**. In that case the metabCombiner anchors should end up being near to randomly selected multiple matches and it would not be possible to model RT correctly due to too many outliers and no good RT inter-dataset shift to model. In this example, because the inter-dataset RT shift is small, metabCombiner seems to be successful at matching the two datasets. Nevertheless, by looking at the figure below, it seems that M2S still does a better job at modelling the RT shift.

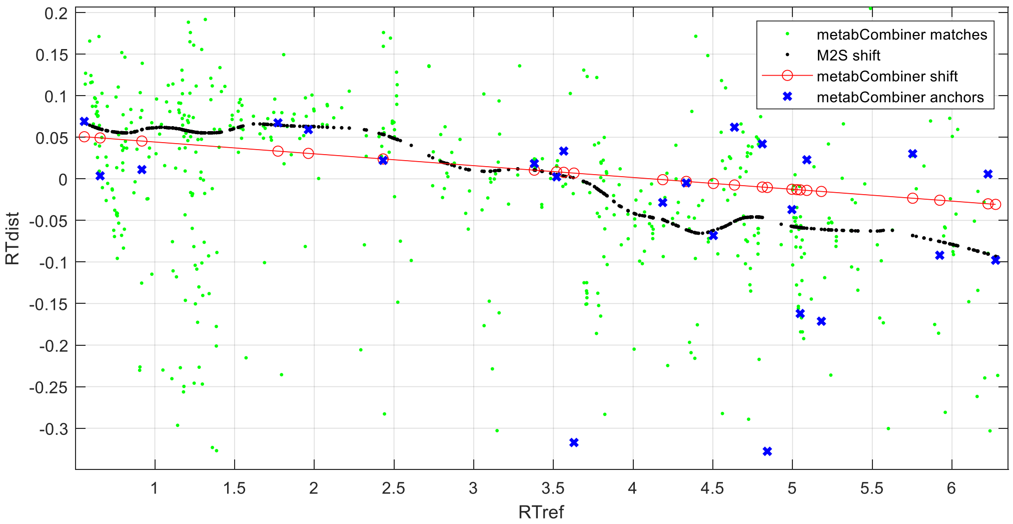

Figure SE6 11: Comparison of inter-dataset shift for the RT dimension obtained by metabCombiner and M2S. Both methods seem to perform appropriately.

As in the previous case, the lack of visualisation in the metabCombiner method made it difficult to define appropriate limits for both RT and MZ thresholds. We realised that the metabCombiner thresholds may have been too generous, thus finding extra matches that in the matching using M2S were left out on purpose by the first threshold definition.

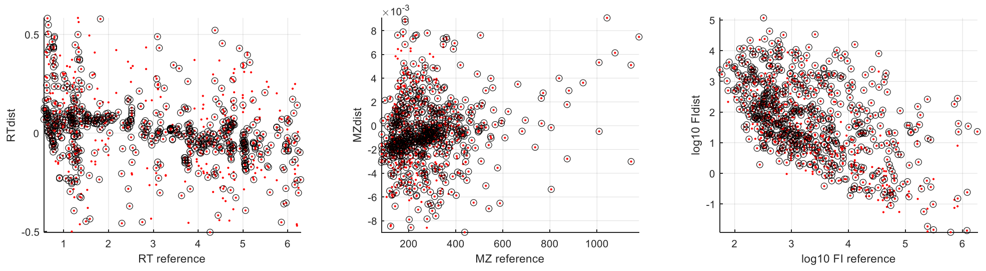

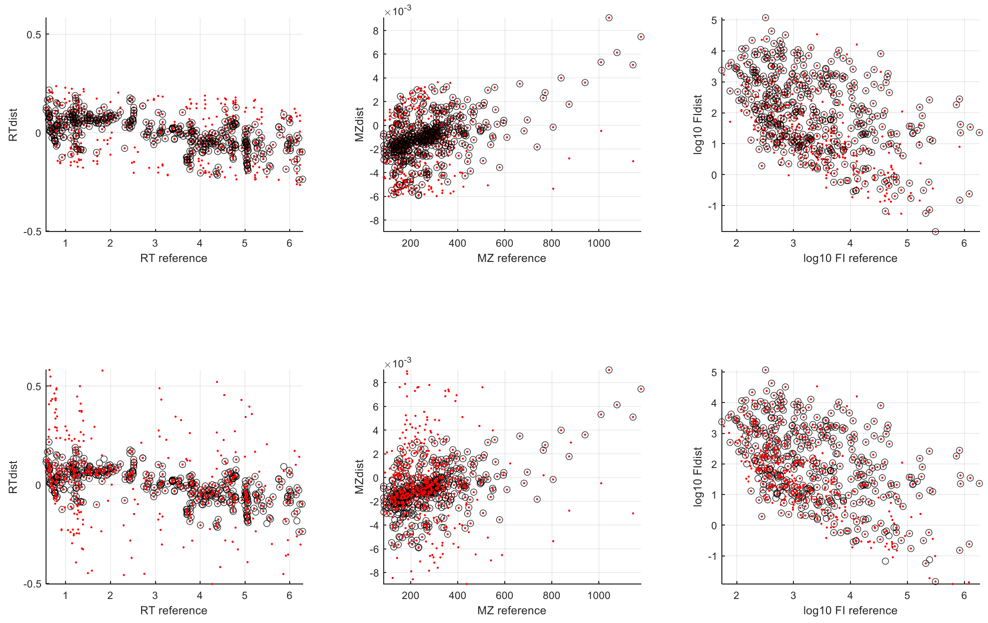

Figure SE6 12: Results using (top) metabCombiner, (middle) M2S, and (bottom) 1-to-1 matches comparison between M2S and metabCombiner. In the top and middle plots the red dots are multiple matches and the black circles are final 1-to-1 matches. In the bottom plot the black circles are M2S and the red dots are metabCombiner final 1-to-1 matches. By looking at the distance to the core of the inter-dataset shifts, it seems that the metabCombiner thresholds may have been too relaxed.

We tried to understand the choices made by metabCombiner and M2S when deciding the best match within a cluster. The plot below shows those results. The red and blue circles are matches in which the reference feature is the same in both metabCombiner and M2S, but the target feature differs. It seems apparent that the red circles representing M2S matches are closer to the trends both in the RT and MZ dimensions than the blue circles representing metabCombiner, which could indicate better results by M2S.

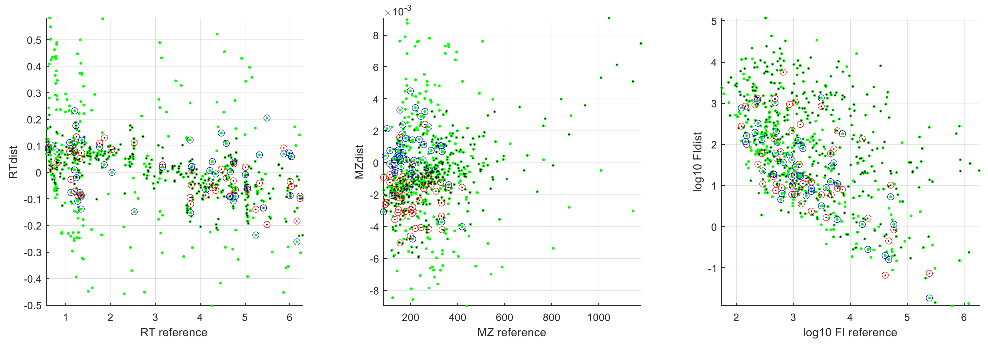

Figure SE6 13: Results from matching with metabCombiner and M2S. Comparison between selected best matches from clusters of multiple matches. These are the same plots as in the figure before, the RT dimension was zoomed in the y-axis for better visualisation. Green dots are metabCombiner matches, black dots are M2S matches (many are superimposed over metabCombiner matches). Red circles are M2S matches with same reference feature as metabCombiner, but different target feature. Blue circles are metabCombiner matches with same reference feature as M2S, but different target feature.

From an initial number of 10985 reference and 6924 target features, M2S found 741 clusters which yielded 451 unique matches, while metabCombiner found 906 clusters, yielding 643 matches. From these, 403 matches were exactly the same in M2S and metabCombiner. M2S found 48 matches that were not found in metabCombiner, 48 of them containing a reference or target feature that was matched to something else in metabCombiner, and zero matches with ref and target features both different from metabCombiner. Similarly, metabCombiner found 240 matches that were not

reciprocated in M2S, 48 of them containing a ref or target feature that was matched to some other in M2S, while it also contained 192 matches inexistent in M2S.

As mentioned previously, the difference in the final "1-to-1 matches" (643/451) reflects the fact that it was not easy to select the right parameters in metabCombiner because of the lack of visualisations, thus we may have been too generous when defining metabCombiner thresholds. In this example, the 48 different matches are (close to 100% of them) matches with the same reference but different target. By looking at their distances to the inter-dataset shifts, we suggest that those matches were better chosen by M2S than metabCombiner.

Table SE6 1: Number of features and matches found in the different phases of the matching.

Match type	M2S	metabCombiner
Features to match (ref/target)	10985 / 6924	
Clusters of matches	741	906
1-to1 matches	451	643
Common matches	403	
Different matches	48	240
With different ref OR target	48	48
With different ref AND target	0	192

Finally, the matches of M2S were investigated to check if the annotation of their reference and target features were the same. Annotation of some of the largest peaks in serum and corresponding matching features in the urine was performed. Features, including adducts and isotopologues, were annotated to confidence level 2 according to the Metabolomics Standards Initiative [13]. This was done matching accurate mass, isotopic distributions and fragmentation spectra (from MS^E all-ion fragmentation scans) to reference data from an in-house standards database and online databases METLIN [14], HMDB [15], GNPS [16] and MassBank [17]. From the 40 annotated features in both reference and target dataset, M2S found 38 correctly matched and 2 incorrectly matched. Similarly, metabCombiner also found 38 correctly matched and 2 incorrectly matched. The incorrectly matched were not the same in the two methods. The figures and table below show those results.

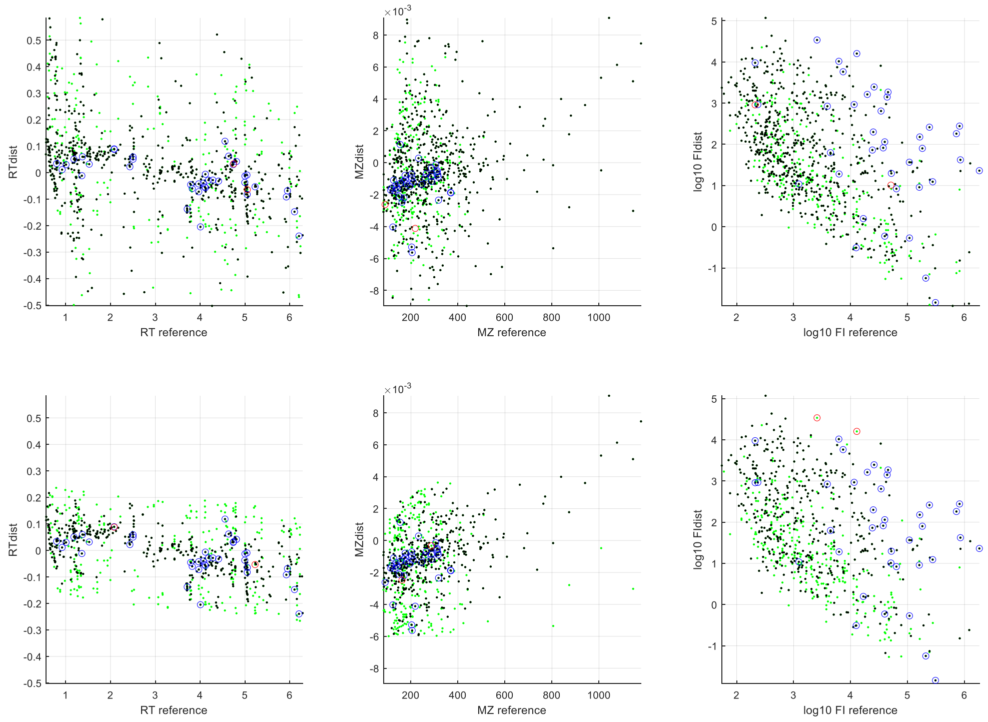

Figure SE6 14: (top) metabCombiner and (bottom) M2S matches results in all dimensions. The plots show matches not selected from multiple match clusters (green dots), final matches (black dots), correctly matched according to manual annotations (blue circles) and incorrectly matched according to annotations (red circles).

Table SE6 2: Results of matching according to annotation of features in 40 matches. According to the annotations, both methods found 38 matches in which both reference and target have the same annotation. Both methods failed at finding 2 correct matches, though those were different in the two methods

reference MZRTstr	target_MZRTstr	Metabolite annotation	M2S	mC
ref_195.0887_0.7977	target_195.0872_0.8319	Caffeine	TRUE	TRUE
ref_181.0727_0.9185	target_181.0714_0.9296	Theobromine and/or Paraxanthine	TRUE	TRUE
ref_123.0601_1.0075	target_123.0560_1.0377	Niacinamide	TRUE	TRUE
ref_209.0566_1.1823	target_209.0554_1.2332	Pseudouridine	TRUE	TRUE
ref_203.0534_1.3600	target_203.0481_1.3483	Hexoses (glucose, fructose, mannose)	TRUE	TRUE
ref_305.0860_1.3728	target_305.0859_1.4326	1-Methylinosine	TRUE	TRUE
ref_137.0465_1.5242	target_137.0451_1.5561	Hypoxanthine	TRUE	TRUE
ref_114.0673_2.4318	target_114.0656_2.4539	Creatinine	TRUE	TRUE
ref_126.0228_2.5027	target_126.0217_2.5534	Taurine	TRUE	TRUE
ref_169.9867_2.5031	target_169.9845_2.5622	Taurine	TRUE	TRUE
ref_205.0976_3.7103	target_205.0920_3.5727	L-Tryptophan	TRUE	TRUE
ref_370.2955_3.7878	target_370.2936_3.7424	Acylcarnitine (14:1) Myristoleoylcarnitine	TRUE	TRUE
ref_368.2799_3.8221	target_368.2781_3.7620	Acylcarnitine (14:2) - Tetradecadiencarnitine	TRUE	TRUE
ref_317.2522_3.9674	target_317.2498_3.9248	Acylcarnitine (10:0) - Decanoylcarnitine	TRUE	TRUE
ref_316.2490_3.9688	target_316.2481_3.8968	Acylcarnitine (10:0) - Decanoylcarnitine	TRUE	TRUE
ref_312.2172_4.0715	target_312.2167_4.0315	Acylcarnitine (10:2) Decadienoylcarnitine	TRUE	TRUE
ref_288.2178_4.1023	target_288.2166_4.0484	Acylcarnitine (8:0) - Octanoylcarnitine	TRUE	TRUE
ref_151.1450_4.1155	target_151.1428_4.1093	Trimethylamine-N-oxide (TMAO)	TRUE	TRUE
ref_310.2021_4.1167	target_310.2016_4.0596	Acylcarnitine (10:3) Decatrienoylcarnitine	TRUE	TRUE
ref_286.2023_4.1855	target_286.2014_4.1570	Acylcarnitine (8:1) Octenoylcarnitine	TRUE	TRUE
ref_260.1863_4.2836	target_260.1853_4.2552	Acylcarnitine (6:0) - Hexanoylcarnitine	TRUE	TRUE
ref_246.1706_4.4109	target_246.1693_4.3788	Acylcarnitine (5:0) - Isovalerylcarnitine	TRUE	TRUE
ref_232.1547_4.5543	target_232.1550_4.6726	Acylcarnitine (4:0) - Butyrylcarnitine	TRUE	TRUE
ref_118.0873_4.6357	target_118.0857_4.6978	Betaine	TRUE	TRUE
ref_138.0558_4.7378	target_138.0542_4.7680	Trigonelline	TRUE	TRUE
ref_218.1392_4.7464	target_218.1351_4.7792	Propionylcarnitine	TRUE	FALSE
ref_144.1028_4.8103	target_144.1012_4.8522	Proline betaine	TRUE	TRUE
ref_204.1240_4.9959	target_204.1230_4.9589	Acetylcarnitine	TRUE	TRUE
ref_132.0777_5.0000	target_132.0759_4.9870	Creatine	TRUE	TRUE
ref_154.0595_5.0440	target_154.0607_5.0347	Creatine	TRUE	TRUE
ref_176.0413_5.0497	target_176.0403_4.9673	Creatine	TRUE	TRUE
ref_162.1141_5.2248	target_162.1116_5.1714	Carnitine	FALSE	TRUE
ref_175.1193_5.9231	target_175.1186_5.8313	L-Arginine	TRUE	TRUE
ref_203.1505_5.9476	target_203.1498_5.8790	Asymmetric dimethylarginine	TRUE	TRUE
ref_170.0927_6.1054	target_170.0916_5.9576	3-Methylhistidine	TRUE	TRUE
ref_156.0769_6.2055	target_156.0756_5.9660	L-Histidine	TRUE	TRUE
ref_314.2334_4.0061	target_314.2327_3.8013	Acylcarnitine (10:1) Decenoylcarnitine	TRUE	TRUE
ref_90.0565_5.0517	target_90.0539_4.9870	Creatine	TRUE	FALSE
ref_265.1188_2.0807	target_265.1181_2.1701	Phenylacetylglutamine	TRUE	TRUE
ref_287.1007_2.0822	target_287.1003_2.1701	Phenylacetylglutamine	FALSE	TRUE

REFERENCES

(1) Bild, D. E.; Bluemke, D. A.; Burke, G. L.; Detrano, R.; Diez Roux, A. V.; Folsom, A. R.; Greenland, P.; Jacob, D. R., Jr.; Kronmal, R.; Liu, K.; et al. Multi-Ethnic Study of Atherosclerosis: objectives and design. *Am J Epidemiol* **2002**, *156* (9), 871-881. DOI: 10.1093/aje/kwf113.

(2) Hofman, A.; Darwish Murad, S.; van Duijn, C. M.; Franco, O. H.; Goedegebure, A.; Ikram, M. A.; Klaver, C. C.; Nijsten, T. E.; Peeters, R. P.; Stricker, B. H.; et al. The Rotterdam Study: 2014 objectives and design update. *Eur J Epidemiol* **2013**, *28* (11), 889-926. DOI: 10.1007/s10654-013-9866-z.

(3) Plumb, R. S.; Johnson, K. A.; Rainville, P.; Smith, B. W.; Wilson, I. D.; Castro-Perez, J. M.; Nicholson, J. K. UPLC/MSE; a new approach for generating molecular fragment information for biomarker structure elucidation. *Rapid Communications in Mass Spectrometry* **2006**, *20* (13), 1989-1994. DOI: 10.1002/rcm.2550.

(4) Smith, C. A.; Want, E. J.; O'Maille, G.; Abagyan, R.; Siuzdak, G. XCMS: processing mass spectrometry data for metabolite profiling using nonlinear peak alignment, matching, and identification. *Anal Chem* **2006**, *78* (3), 779-787. DOI: 10.1021/ac051437y.

(5) Murgia, A.; Hinz, C.; Liggi, S.; Denes, J.; Hall, Z.; West, J.; Santoru, M. L.; Piras, C.; Manis, C.; Usai, P.; et al. Italian cohort of patients affected by inflammatory bowel disease is characterised by variation in glycerophospholipid, free fatty acids and amino acid levels. *Metabolomics* **2018**, *14* (10), 140. DOI: 10.1007/s11306-018-1439-4.

(6) Liggi, S.; Hinz, C.; Hall, Z.; Santoru, M. L.; Poddighe, S.; Fjeldsted, J.; Atzori, L.; Griffin, J. L. KniMet: a pipeline for the processing of chromatography-mass spectrometry metabolomics data. *Metabolomics* **2018**, *14* (4), 52. DOI: 10.1007/s11306-018-1349-5.

(7) Elliott, P.; Vergnaud, A. C.; Singh, D.; Neasham, D.; Spear, J.; Heard, A. The Airwave Health Monitoring Study of police officers and staff in Great Britain: rationale, design and methods. *Environ Res* **2014**, *134*, 280-285. DOI: 10.1016/j.envres.2014.07.025.

(8) Izzi-Engbeaya, C.; Comninos, A. N.; Clarke, S. A.; Jomard, A.; Yang, L.; Jones, S.; Abbara, A.; Narayanaswamy, S.; Eng, P. C.; Papadopoulou, D.; et al. The effects of kisspeptin on beta-cell function, serum metabolites and appetite in humans. *Diabetes Obes Metab* **2018**, *20* (12), 2800-2810. DOI: 10.1111/dom.13460.

(9) Lewis, M. R.; Pearce, J. T.; Spagou, K.; Green, M.; Dona, A. C.; Yuen, A. H.; David, M.; Berry, D. J.; Chappell, K.; Horneffer-van der Sluis, V.; et al. Development and Application of Ultra-Performance Liquid Chromatography-TOF MS for Precision Large Scale Urinary Metabolic Phenotyping. *Anal Chem* **2016**, *88* (18), 9004-9013. DOI: 10.1021/acs.analchem.6b01481.

(10) Schiffman, C.; Petrick, L.; Perttula, K.; Yano, Y.; Carlsson, H.; Whitehead, T.; Metayer, C.; Hayes, J.; Rappaport, S.; Dudoit, S. Filtering procedures for untargeted LC-MS metabolomics data. *BMC Bioinformatics* **2019**, *20* (1), 334. DOI: 10.1186/s12859-019-2871-9.

(11) Habra, H.; Kachman, M.; Bullock, K.; Clish, C.; Evans, C. R.; Karnovsky, A. metabCombiner: Paired Untargeted LC-HRMS Metabolomics Feature Matching and Concatenation of Disparately Acquired Data Sets. *Analytical Chemistry* **2021**, *93* (12), 5028-5036. DOI: 10.1021/acs.analchem.0c03693.

(12) Habra, H. *Combine LC-MS Metabolomics Datasets with metabCombiner*. 2021. https://bioconductor.org/packages/release/bioc/vignettes/metabCombiner/inst/doc/metabCombiner_vignette.html (accessed 2022 3 January 2022).

(13) Sumner, L. W.; Amberg, A.; Barrett, D.; Beale, M. H.; Beger, R.; Daykin, C. A.; Fan, T. W.; Fiehn, O.; Goodacre, R.; Griffin, J. L.; et al. Proposed minimum reporting standards for chemical analysis Chemical Analysis Working Group (CAWG) Metabolomics Standards Initiative (MSI). *Metabolomics* **2007**, *3* (3), 211-221. DOI: 10.1007/s11306-007-0082-2.

(14) Guijas, C.; Montenegro-Burke, J. R.; Domingo-Almenara, X.; Palermo, A.; Warth, B.; Hermann, G.; Koellensperger, G.; Huan, T.; Uritboonthai, W.; Aisporna, A. E.; et al. METLIN: A Technology Platform for Identifying Knowns and Unknowns. *Anal Chem* **2018**, *90* (5), 3156-3164. DOI: 10.1021/acs.analchem.7b04424.

(15) Wishart, D. S.; Feunang, Y. D.; Marcu, A.; Guo, A. C.; Liang, K.; Vazquez-Fresno, R.; Sajed, T.; Johnson, D.; Li, C.; Karu, N.; et al. HMDB 4.0: the human metabolome database for 2018. *Nucleic Acids Res* **2018**, *46* (D1), D608-D617. DOI: 10.1093/nar/gkx1089.
(16) Wang, M. X.; Carver, J. J.; Phelan, V. V.; Sanchez, L. M.; Garg, N.; Peng, Y.; Nguyen, D. D.; Watrous, J.; Kapono, C. A.; Luzzatto-Knaan, T.; et al. Sharing and community curation of mass spectrometry data with Global Natural Products Social Molecular Networking. *Nature Biotechnology* **2016**, *34* (8), 828-837. DOI: 10.1038/nbt.3597.
(17) Horai, H.; Arita, M.; Kanaya, S.; Nihei, Y.; Ikeda, T.; Suwa, K.; Ojima, Y.; Tanaka, K.; Tanaka, S.; Aoshima, K.; et al. MassBank: a public repository for sharing mass spectral data for life sciences. *J Mass Spectrom* **2010**, *45* (7), 703-714. DOI: 10.1002/jms.1777.
